# Unlocking Nature’s Shield: The Promising Potential of CRISPRa in Amplifying Antimicrobial Peptide Expression in Common Bean (*Phaseolus vulgaris* L.)

**DOI:** 10.1021/acsomega.4c09817

**Published:** 2025-02-06

**Authors:** Mariana Rocha Maximiano, Lucas José de Sousa, Gabriel Cidade Feitosa, Maria Eduarda Melo Lopes, Brisa Ortega, Raquel dos Santos Madeiro, Fabiano Touzdjian Pinheiro Kohlrausch Távora, Bruna Medeiros Pereira, Osmundo Brilhante de Oliveira Neto, Cirano José Ulhôa, Ana Cristina Miranda Brasileiro, Francisco José Lima Aragão, Angela Mehta, Octávio Luiz Franco

**Affiliations:** † Universidade Católica de Brasília, Centro de Análises Proteômicas e Bioquímicas, Programa de Pós-Graduação em Ciências Genômicas e Biotecnologia, Brasília CEP: 71966-700, Distrito Federal, Brazil; ‡ Universidade Católica Dom Bosco, S-Inova Biotech, Pós-Graduação em Biotecnologia, Campo Grande CEP: 79117-900, Mato Grosso do Sul, Brazil; § Universidade de Brasília, Brasília CEP: 70910-900, Distrito Federal, Brazil; ∥ Embrapa Recursos Genéticos e Biotecnologia, Brasília CEP: 70770-917, Distrito Federal, Brazil; ⊥ Centro Universitário do Distrito Federal, Brasília CEP: 70390-030, Distrito Federal, Brazil; # Symbiomics, Florianópolis CEP: 88050-000, Santa Catarina, Brazil; ∇ Universidade Federal do Goiás, Goiânia 74690-900, Goiás, Brazil

## Abstract

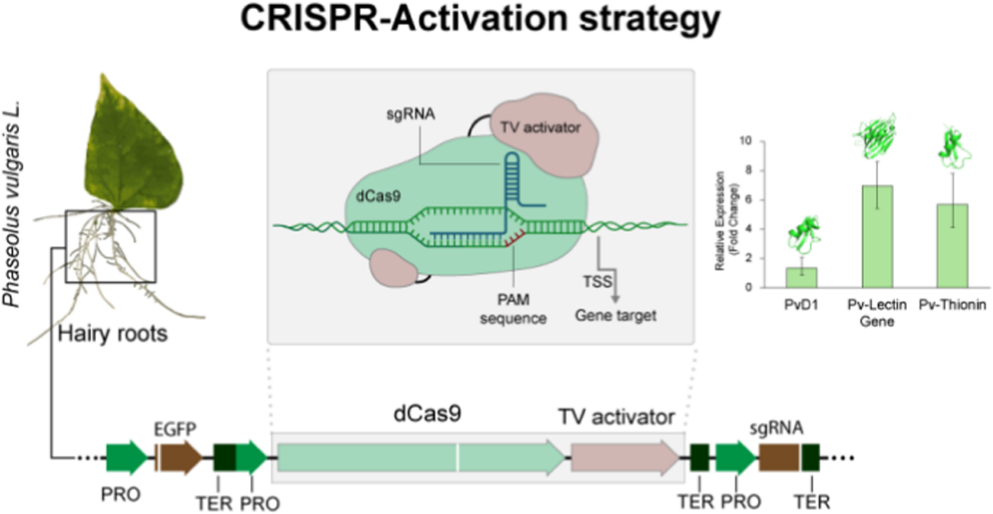

This study proposes using the CRISPR transcriptional activation strategy to modulate the expression of genes encoding defense proteins and antimicrobial peptides (AMPs) in *Phaseolus vulgaris*. Three genes (PvD1, Pv-thionin, and Pv-lectin) were selected and targeted by the CRISPR–dCas9–TV-mediated transcriptional activation complex in the *P. vulgaris* L. hairy root. RT-qPCR investigated their activation efficiency. The eGFP-positive transgenic hairy roots exhibit enhanced expression of targeted genes compared to that of control roots. A moderate increase of 1.37-fold in PvD1 gene expression was observed in transgenic hairy roots, while 6.97-fold (Pv-lectin) and 5.70-fold (Pv-thionin) increases were observed. Importantly, no off-target effects of sgRNAs were detected, ensuring the precision and safety of the CRISPR–dCas9–TV strategy. The present article is a proof-of-concept study, and it has succeeded in demonstrating the efficiency of the CRISPR–dCas9–TV strategy in modulating the expression of target genes in *P. vulgaris*, paving the way for an alternative approach to protecting such essential crop plants.

## 1 Introduction

Common bean (*Phaseolus vulgaris* L.) can source numerous human nutrients, including proteins, carbohydrates, and minerals.^1^ According to the Food and Agriculture Organization of the United Nations (FAO), global common bean production in the form of dry seeds was around 27.7 million tons in 2021.^2^ Nonetheless, diseases caused by phytopathogens have led to severe grain yield losses worldwide.^3^ Throughout evolution, plants have coevolved with a wide range of phytopathogens that are currently responsible for essential diseases in common beans. These include several bacteria, such as *Clavibacter michiganensis* (bacterial leaf yellowing of bean),^4^*Curtobacterium flaccumfaciens* (bacterial wilt),^5^*Erwinia chrysanthemi* (soft rot disease),^6,7^*Pseudomonas syringae* (halo blight disease),^8^ and *Xanthomonas* spp. (common bacterial blight),^9^ and fungi such as *Fusarium solani* (Fusarium root rot), *Macrophomina phaseolina* (charcoal rot of bean), *Rhizoctonia solani* (Rhizoctonia root rot), and *Sclerotinia sclerotiorum* (white mold).^10^

Plant–pathogen interactions may result in refined plant defense mechanisms, which include physical barriers, phytohormone signaling, and polypeptide compound synthesis. The physical barriers, such as waxy cuticular layers and trichomes, can make initial phytopathogen infection more difficult.^11^ The signaling of defense-related phytohormone pathways, including ethylene (ET), salicylic acid (SA), and jasmonic acid (JA), and the complex crosstalk between these pathways play a direct role in the regulation of pathogen resistance responses in plants.^12^ Moreover, the synthesis of polypeptide compounds can initiate immune response cascades through signal transduction and processes of pathogen recognition.^13^ Among such polypeptides synthesized in response to biotic stresses are the antimicrobial peptides (AMPs) and proteins, which may establish general chemical barriers, acting as the primary line of defense against phytopathogens.^4,14,15^

Recent studies have been reinforcing the importance of antimicrobial peptides (AMPs) in plant defense mechanisms.^4,16−19^ Studies also examined the structure, biological function, and transgenic applications of defensins and thionins, highlighting their utility in improving crop resistance to phytopathogens.^18−20^ These findings underscore the pivotal role of AMPs as valuable targets for biotechnological advancements in crop protection and improvement.

However, phytopathogens have also developed many adaptations to circumvent these host defense mechanisms. In general, plant diseases are mitigated through integrated pest management (IPM) strategies, which include conventional cultural practices,^21^ chemical methods, and biocontrol approaches.^22,23^ Moreover, contemporary agriculture has an increasing demand for increasingly effective and environmentally friendly solutions to safeguard crops against plant diseases, including biotechnological breakthroughs that have contributed significantly to advances in plant breeding.^24−26^ A recent milestone in CRISPR/Cas genome editing technology has provided potential and alternative strategies to improve plant crop resistance.^27−30^

Biotechnological approaches such as genome editing have revolutionized agricultural research, providing precise tools for enhancing plant defenses. The CRISPR/Cas system, in particular, allows for the targeted modulation of gene expression, including the activation of genes encoding defense-related peptides, which holds promise for addressing current agricultural challenges.

The CRISPR/Cas system has emerged as a potent biotechnological tool for improving agriculture, enabling researchers to precisely manipulate the eukaryote genome, including in plants.^27,30−32^ This programmable RNA-based genome editing system, which conventionally employs an endonuclease (e.g., Cas9) driven by a guide-RNA transcript to promote a double-strand break (DSB) in a target DNA sequence, offers many genome editing strategies, including gene knockout, knock-in of DNA sequences, conversion of DNA nucleobases, and gene expression modulation.^28,33,34^

Gene expression modulation includes the inhibition (CRISPRi) and activation (CRISPRa) of a target gene transcription.^30^ Particularly interesting, the CRISPRa strategy utilizes a mutated Cas9 enzyme named dead Cas9 (dCas9), without endonuclease activity, fused to transcriptional activator (TA) molecules, which makes it possible to enhance gene expression at manifold levels.^35,36^ The CRISPR strategy has been employed to boost the desirable crop traits. Recently, this strategy has been used to create three novel cotton germplasm materials.^37^ In tomatoes, CRISPRa was used to activate the SlPR-1 gene, aiming to increase resistance against biotic stress,^38^ and in maize to activate ZmBBM2, resulting in parthenogenesis induction.^39^

Despite significant progress in genome editing technologies, the application of CRISPR-based approaches to *P. vulgaris* remains underexplored. To address this gap, the present study evaluates the efficacy of the CRISPR–dCas9–TV transcriptional activation system in upregulating key defense-related genes in *P. vulgaris* by targeting defensins and lectins, which are two classes of antimicrobial peptides with proven antimicrobial activity. The selected lectin (∼30 kDa), isolated from *P. vulgaris* cv. “Anasazi Bean” seeds, shows broad-spectrum activity with antiviral properties.^40^ The selected AMPs are the defensin, PvD1 (∼5 kDa), isolated from *P. vulgaris* seeds, with remarkable antifungal activity;^41^ and Cp-thionin II (∼5 kDa), isolated from *Vigna unguiculata* (cowpea) seeds, with antibacterial activity.^42^ This research aims to advance our understanding of sustainable strategies for enhancing crop protection and resilience against phytopathogens.

## 2 Materials and Methods

### 2.1 Target Gene Selection

**Figure 1:**
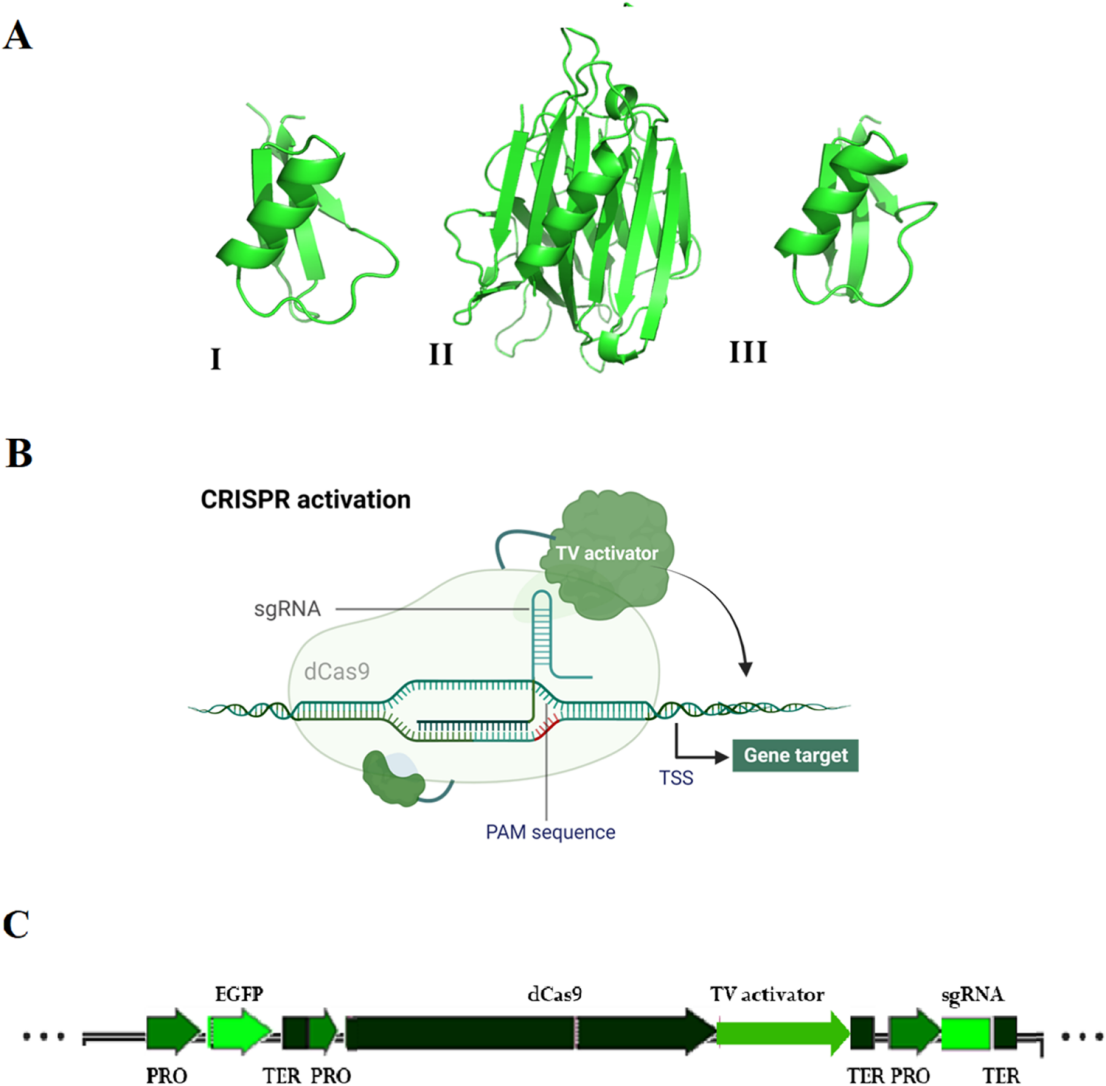
(A) Structure targets: PvD1 (I), Pv-lectin (II), and Pv-thionin (III). Panel (B) used the CRISPR activation strategy. TV activator, 6X TAL fused in tandem to VP128 activation; TSS, transcription start site. PAM Sequence, protospacer adjacent motif sequence. (C) Vector strategy. PRO, promoter; EGFP, GFP gene reporter; TER, terminator; dCas9, dCas9 gene; TV activator, 6X TAL fused in tandem to VP128 activation; sgRNA, gene-specific spacers. Created with Biorender (BioRender.com) support.

Three plant polypeptide defense compound genes were selected. Two of them were isolated from common bean (*P. vulgaris*) seeds: a lectin (PHAVU_004G158200g),^40^ here designated as Pv-lectin, and a PvD1 defensin (PHAVU_005G071300g).^41^ Additionally, one *P. vulgaris* gene with an 86% similarity with the Cp-thionin II^42^ isolated from *Vigna unguiculata* (cowpea) was selected for this study and here designated as Pv-thionin (PHAVU_002G278400g). The molecular modeling of these three structures (Figure 1A) was performed using the Alphafold2 tool.^43,44^

### 2.2 CRISPR GFP–dCas9–TV Vector and sgRNA Design

The CRISPR final constructs were formulated to express the enhanced green fluorescent protein (*GFP*) reporter gene, the bar gene for selecting resistance to glufosinate ammonium herbicide, and dCas9–TV, harboring six copies of the transcription activator-like (TAL) activation domain fused in tandem to eight copies of the viral protein 16 (VP128) gene activator, which has pPZP^45^ and pDGB3 alpha2^46,47^ plasmids as the backbone. Spacer sequences (20 nt sgRNA) targeting specific regions on the promoter region of selected genes were designed (Table S1) using a CRISPR-assisted website (available at http://crispor.tefor.net/).^48^ The CRISPR tool predicted potential off-target activation (available at http://crispor.tefor.net/)^48^ for each sgRNA designed. All CRISPR vectors used in the study were synthesized by Epoch Life Science and are named here as pPZP_CRISPRa_PvD1 (Figure S1), pPZP_CRISPRa_Pv-lectin (Figure S2), and pPZP_CRISPRa_Pv-thionin (Figure S3). An empty vector (without sgRNA sequence), pPZP_CRISPRa (Figure S4), was also synthesized and used as a negative control.

### 2.3 Detection of sgRNA Target Sites in *P. vulgaris* cv. Olathe Pinto

After CRISPR vector construction, specific primers (Table S2) were designed to amplify the genomic region that encoded the dCas9 coupling site (sgRNA target sites) performed by a polymerase chain reaction (PCR). Leaves of *P. vulgaris* cv. Olathe Pinto were collected, and DNA isolation was performed^49^ to detect the genomic region that encoded the sgRNA target sites. PCR was performed to verify the presence of sgRNAs in each transgenic line, using 10 ng of genomic DNA, 1 U of polymerase DNA (Taq DNA polymerase GE Healthcare Life Sciences), 1× PCR reaction buffer (GE Healthcare Life Sciences), 1 μM of each specific primer, 250 μM dNTPs, and 2.5 mM MgCl_2_. The reaction was conducted in a Veriti 96 Well Thermal Cycler (Applied Biosystems) with a program of 95 °C for 3 min, 30 cycles of 95 °C for 30 s, 55 °C for 30 s, and 72 °C for 40 s, and a last step of 72 °C for 5 min. Visualization of the PCR products was performed on a 2% agarose gel stained with ethidium bromide. The verification of PCR products was confirmed by Sanger’s sequencing.

### 2.4 *P. vulgaris* Hairy Roots Transformed for Gene Modulation

*P. vulgaris* cv. Olathe Pinto, at 25 days after germination, was used for the CRISPR-mediated gene expression modulation assay. Twenty healthy trifoliate leaves were collected from 10 different plants. Terminal leaflets were detached from the trifoliate leaves and disinfected, and each removed petiole was inoculated with the *Agrobacterium rhizogenes* “K599” strain harboring the binary vectors, using a sterile needle with the bacterial suspension, as described by Pereira et al.^50^ Subsequently, the inoculated leaves were kept under growth chamber conditions (25 ± 2 °C; 12 h photoperiod; 120 μmols/m^2^/s^1^ light intensity). Ten days after *A. rhizogenes* transformation, the developed hairy roots were assessed for GFP fluorescence under an M205 stereomicroscope (Leica Microsystem, Wetzlar, Germany). eGFP-positive hairy roots were individually collected, homogenized in liquid nitrogen, and stored at −80 °C for further analysis. To further confirm its transgenic status, DNA was extracted from each eGFP-positive hairy root, and the dCas9 fragment was amplified by PCR, followed by Sanger sequencing of the amplicon.

### 2.5 Evaluation of Gene Expression Modulation

Total RNAs of five GFP- and PCR-positive hairy roots obtained after transformation with each CRISPR binary vector (sgRNAs for PvD1, Pv-lectin, and Pv-thionin genes) were extracted and purified using concert plant reagent,^51^ according to the manufacturer’s instructions. Each GFP-positive hairy root originates from a single cell, representing a distinct transgenic event (biological replicate). The integrity of total RNA was validated through electrophoresis, and its concentration was determined using a NanoDrop ND-1000 spectrophotometer (Thermo Scientific, Waltham, MA). Following the manufacturer’s instructions, total RNA underwent treatment with 2 U of Turbo DNase (Applied Biosystems/Ambion, Foster City, CA) to eliminate potential genomic DNA contamination. According to the manufacturer’s instructions, cDNA synthesis was performed using 1 μg of total RNA and the kit Go Script Reverse Transcription System (Promega, Madison, WI).

The qRT-PCR reactions were carried out using specific primers (Table 1) on the thermal cycler 7300 real-time polymerase chain reaction (PCR) System (Applied Biosystems, Foster City, CA, USA) as described by Maximiano et al.^52^ All reactions were performed using three independent biological replicates and three technical replicates per sample (*n* = 9). Fluorescence raw data were imported to the Real-time PCR Miner software^53^ to determine the cycle quantification (Cq) values and the PCR efficiency. The relative gene expression and statistics analyses were performed using REST software, employing the genes Ef1α and actin as the internal control.^54^

**Table 1:** General Information about Specific qRT-PCR Primers

identification	forward primer (5′ → 3′)	*T*_m_ °C	reverse primer (5′ → 3′)	*T*_m_ °C	amplicon size
PvD1	GCAAAGACTTGCGAGAACCT	59.60	ACCTGCCACTCCTCAAGTGT	59.80	106
Pv-lectin	GTCATATTGGCATCGACGTG	60.00	AGAGCTTCGTGGAGGAGTCA	60.10	114
Pv-thionin	AGATGTGGCGGTGAAGAAAG	60.30	ATGAACGTGCACAGGTGAAA	60.20	95
off-target for PVD1 sgRNAs	GGAGAAGAAGGCACCATAGA	56.32	TCTGTTGAAGGGGTGGTAGT	57.89	117
off-target for Pv-lectin sgRNAs	GTTCGACACCTACTCCAACC	57.92	GACGCCGTTCTGATAGACTT	57.44	90
off-target for Pv-thionin sgRNAs	TGTCCAGCTACAACATACCG	57.33	TTTCCAGGGTTAACACGAAT	59.87	103
actin^a^	ACAGCCAGGACCAGTTCATC	60.10	TCATGGATGGTTGGAACAGA	59.90	115
Ef1α^a^	GAACTCGAGACAGCCAGGAC	60.00	CTGGACATCTGAAACGCTCA	60.00	100

a Reference gene.

### 2.6 Rich-Polypeptide Fraction Isolation

GPF- and PCR-positive hairy root events (five biological replicates of each pPZP_CRISPRa_PvD1, pPZP_CRISPRa_Pv-lectin, pPZP_CRISPRa_Pv-thionin, and pPZP_CRISPRa negative control) were submitted to rich-polypeptide fraction extraction according to Franco et al.^42^ with modifications. Briefly, the extraction was performed using TRIS buffer (0.05 M Tris–HCl pH 6.8 and 0.15 M NaCl) in a 1:3 proportion (w:v). The suspension was sonicated at a 90% amplitude for 30 s (3 cycles) and centrifuged at 8000*g* at 4 °C for 30 min. The supernatant, containing rich-polypeptide fraction extract, was collected and quantified using a Qubit fluorimeter (Thermo Fisher Scientific, Waltham, MA) by the manufacturer’s recommendations.

### 2.7 Antibacterial Bioassay

The antibacterial potential of obtained peptides was evaluated by estimating the minimum inhibitory concentration (MIC) over the following phytopathogen bacteria: *C. michiganensis* (817), *C. flaccumfaciens* (1376), *E. chrysanthemi* (336), *P. syringae* pv *tomato* (853), *P. syringae* pv *cenoura* (1329), *Ralstonia solanacearum* (VW363), *Xanthomonas phaseoli* pv *fuscans* (772), *Xanthomonas campestris* pv *campestris* (828), *X. campestris* pv *campestris* (51), and *Xanthomonas phaseoli* pv *phaseoli* (BRM25302) were from the Catholic University of Brasilia microorganism collection. All measurements were performed according to Maximiano et al.^55^ Briefly, all bacterial strains were cultured in nutrient broth, and MIC values were estimated using 1 × 10^6^ CFU mL^–1^ of bacterial cells and 8 peptide concentrations in serial dilution ranging from 2 to 256 μg mL^–1^. The microplates were incubated at 28 °C for 24 h, and the readings were performed in a Biotek spectrophotometer (PowerWaveTM HT Microplate Reader) at a wavelength of 595 nm. Antibacterial activity was calculated (in percentage) considering the values of the positive control (Kanamycin, 50 μg mL^–1^) as 100% of inhibition and the negative control (distilled sterile water) defined as 0% of inhibition. The assay was performed with five biological replicates.

### 2.8 Antifungal Bioassay

Antifungal activities of obtained peptides were evaluated by estimating the minimum inhibitory concentration (MIC) of the following phytopathogenic fungi: *S. sclerotiorum*, *M. phaseolina*, *R. solani*, and *F. solani*. These phytopathogenic fungi were obtained from the microorganism collection of Embrapa Rice and Beans (Brazil) and are known to cause diseases in common beans.^56^ All fungal strains were maintained on potato-dextrose-agar (PDA) slants at 4 °C for further use. A 5 mm disc of phytopathogenic fungi was placed in a Petri dish containing PDA medium and incubated at 27 °C for 1 day. Then, four 5 mm discs of sterilized filter paper impregnated with purified peptides (2 to 256 μg mL^–1^) were added next to the colony. The Petri dishes were incubated at 27 °C for 7 days with a 12 h photoperiod. The assay was performed with five biological replicates.

### 2.9 Statistical Analysis

Gene expression and statistics were analyzed using the Relative Expression Software Tool (REST) software.^54^ The antibacterial and antifungal assay analysis compared the differences in control and treated samples; a *t* test was used with four degrees of freedom, *n* = 9 (three biological and three technical replicates for each biological replicate) and 95% confidence.

## 3 Results and Discussion

This study selected three polypeptide defense compounds (Figure 1A) for gene modulation by CRISPR strategy (Figure 1B), including Pv-lectin,^40^ the AMPs PvD1,^41^ and Pv-thionin.^42^ The pPZP_CRISPRa vector (Figure 1C) was designed to direct the transcriptional activation of these genes in *P. vulgaris*, aiming to increase the expression of defense-related genes and, consequently, enhance the plant antimicrobial resistance. In this way, three different sgRNAs targeting the promoter region of PvD1, Pv-lectin, and Pv-thionin (Table S1) were designed, and the coupling sites of each sgRNA were confirmed by Sanger sequencing (Figure S4). Thus, the pPZP_CRISPRa_PvD1 (File S1), pPZP_CRISPRa_Pv-lectin (File S2), pPZP_CRISPRa_Pv-thionin (File S3), and pPZP_CRISPRa vectors (File S4) were generated.

**Figure 2:**
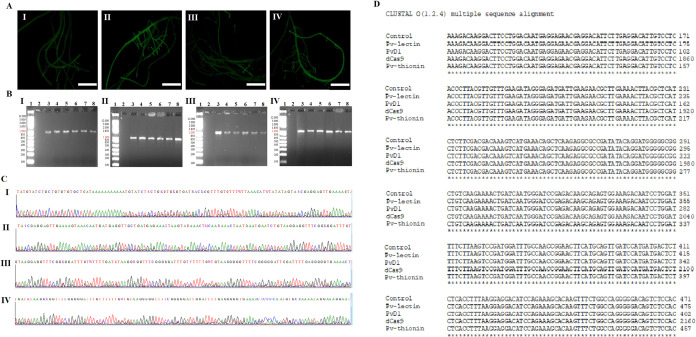
(A) Edited hairy roots expressing GFP. I. pPZP_CRISPRa under ultraviolet light. II. pPZP_CRISPRa_PvD1 under ultraviolet light. III. pPZP_CRISPRa_Pv-lectin under ultraviolet light. IV. pPZP_CRISPRa_Pv-thionin vector under ultraviolet light. Bars were 10 mm. (B) Confirmation of dCas9 presence in the edited hairy root by PCR. I. pPZP_CRISPRa II. pPZP_CRISPRa_PvD1 III. pPZP_CRISPRa_Pv-lectin IV. pPZP_CRISPRa_Pv-thionin. 1. Molecular marker (1Kb plus, Invitrogen). 2. PCR negative control (PCR reaction without a sample). 3–7. Amplification of the dCas9 fragment (∼948 pb) from DNA isolated of five biologic replicates of each edited hairy root group. 8. Positive control (PCR reaction with each correspondent vector as a template). (C) Sanger chromatograms of PCR products. I. pPZP_CRISPRa II. pPZP_CRISPRa_PvD1 III. pPZP_CRISPRa_Pv-lectin IV. pPZP_CRISPRa_Pv-thionin. (D) Alignment of the sequences, obtained by Sanger’s sequencing, with the dCas9 sequence present in each vector used in the edition of hairy roots.

Independent transgenic events were produced for each target gene to evaluate the gene-editing efficiency in *P. vulgaris* hairy roots (Figure S5). Hairy roots were individually examined for GFP fluorescence under UV light, with both nontransformed and hairy roots generated by pPZP_CRISPRa (vector without sgRNA) as negative controls. GFP fluorescence was observed in 59% of obtained hairy roots transformed with pPZP_CRISPRa_PvD1, 51% of obtained hairy roots transformed with pPZP_CRISPRa_Pv-lectin, 64% of obtained hairy roots transformed with pPZP_CRISPRa_Pv-thionin, and 61% of obtained hairy roots transformed with pPZP_CRISPRa (Figure 2A), which indicates that these tissues were transformed with the pPZP_CRISPRa vectors. The efficiency of *A. rhizogenes* transformation was estimated at 60%, the percentage of common bean detached leaves that presented at least one GFP-positive hairy root.

The transgenic status of the GFP-positive hairy roots was also confirmed by PCR, with the dCas9 gene detected in all evaluated samples (Figure 2B) and confirmed by Sanger’s sequencing (Figure 2C,D). These findings illustrate that detached leaves can serve as explants for successfully generating transgenic hairy roots in common beans under *ex vitro* conditions, building upon our prior investigations into peanut and soybean hairy roots.^50,57^ This approach has demonstrated its efficacy as a straightforward, rapid, cost-effective, and space-efficient method for validating CRISPR sgRNAs directly within the target crop plant. It could be further applied as a large-scale strategy for the in-root functional characterization and validation of candidate genes in common beans.

**Figure 3:**
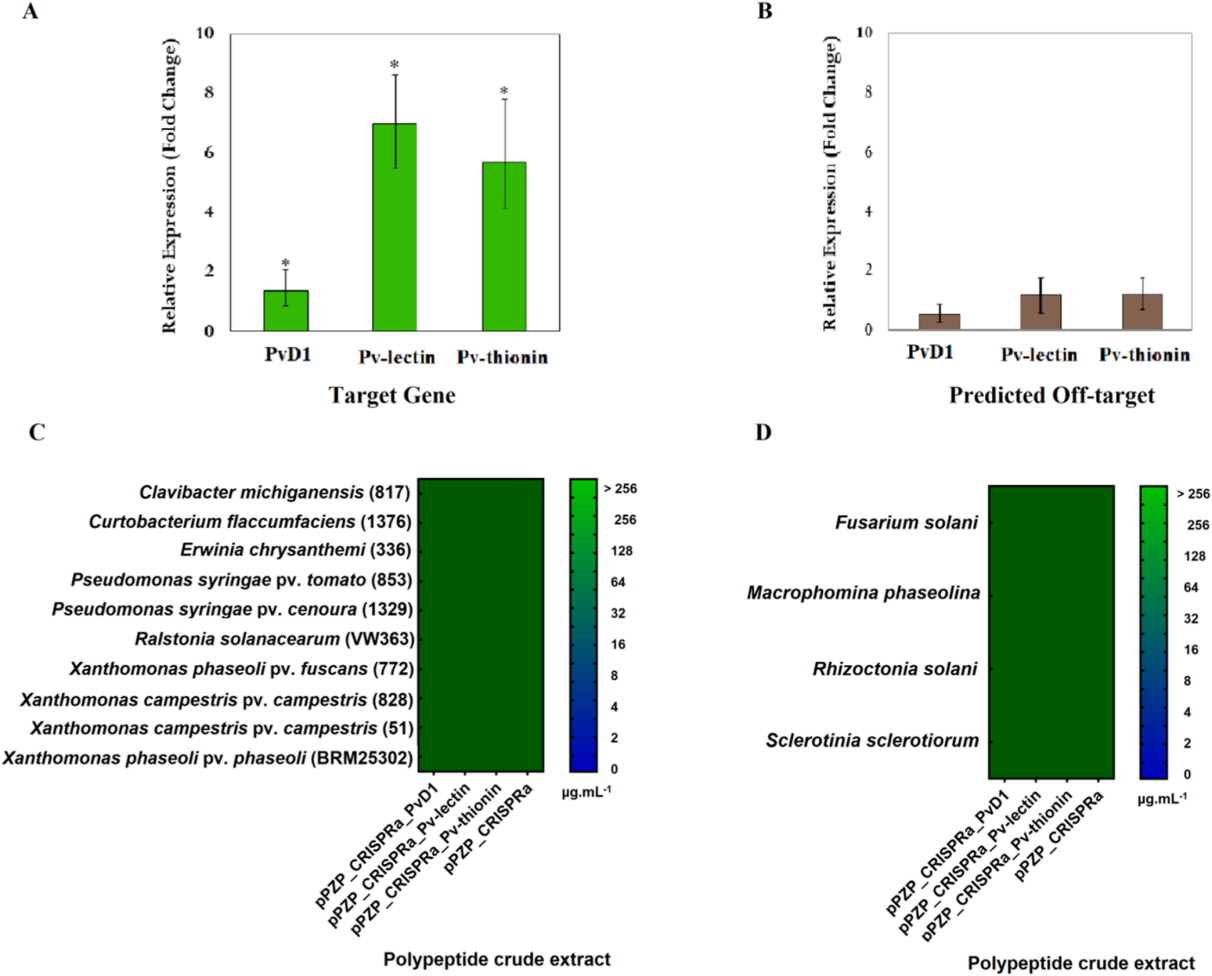
(A) Relative gene expression of PvD1, Pv-lectin, and Pv-thionin after gene modulation. Bars represent the mean ± Std error values. (*) *p* ≤ 0.05 in comparison with the control (hairy roots did not express sgRNAs (pPZP_CRISPRa)). (B) Gene expression evaluation of predicted off-target events for PvD1, Pv-lectin, and Pv-thionin sgRNAs. Bars represent the mean ± std error values. (*) *p* ≤ 0.05 in comparison with the control (hairy roots did not express sgRNAs (pPZP_CRISPRa)). (C) Antibacterial activity. (D) Antifungal activity. Polypeptide crude extract concentrations in serial dilution ranged from 2 to 256 μg mL^–1^.

GFP- and PCR-positive hairy hoot events were then used to evaluate target and off-target gene expressions. Events with activation systems targeting PvD1, Pv-lectin, and Pv-thionin showed an increased expression of all target genes when compared to the negative control (Figure 3A), which did not express sgRNAs (pPZP_CRISPRa). Results presented a moderate increase of 1.37-fold in PvD1 gene expression, while the other two target genes presented an increase of 6.97-fold for Pv-lectin and 5.70-fold for Pv-thionin. These transcriptional activation data showed that the CRISPR/dCas9 strategy significantly increased the number of three distinct *P. vulgaris* target genes using the *ex vitro* hairy root as a model system. Additionally, the off-targets predicted for all sgRNAs did not show modifications in gene expression compared with the control (Figure 3B), confirming the specificity of the sgRNAs designed, given that only target genes were modulated. dCas9–TV was the first CRISPRa strategy employed in plant cells and was initially proposed as a more robust gene activator for plant gene modulation.^46^

The dCas9–TV potential was demonstrated in *Arabidopsis thaliana* plant models (target gene expression was increased from 30- to 510-fold) and in *Oryza sativa* protoplasts (target gene expression was increased from 13- to 79-fold).^46^*O. sativa* plants were also edited employing dCas–TV, showing a target gene expression increase of 1000-fold.^58^ However, studies that edited grape plants employing these same strategies observed an increase in the target gene expression of 3.7- to 42.3-fold.^59^ Most recently, two genes were edited in cotton, employing several different sgRNAs for each target, where the results showed an increase in the target gene expression of 6.4- to 35.5-fold and 6- to 41.7-fold.^37^

In this context, it is essential to highlight the differences between the gene modulations observed, as the increase in gene expression may be related to the species edited or the target gene, sgRNAs, and their target position. The gene activation efficiency can be affected by the targeted sgRNA’s position. The sgRNAs that target the upstream region of the TATA box and transcript start site have a positive correlation with dCas9-mediated gene activation. In contrast, positioning dCas9 downstream or too close to the TATA box can negatively impact gene expression, probably because dCas9 physically blocks the transcription machinery.^60^ However, the optimal positions for sgRNA upstream of the TSS (transcription start site) for increasing gene activation are unclear.^32^

The total protein rich-fraction extract, obtained from edited hairy root events, did not show significant antimicrobial activity against 10 phytobacteria, including *C. michiganensis* (817), *C. flaccumfaciens* (1376), *E. chrysanthemi* (336), *P. syringae* pv *tomato* (853), *P. syringae* pv *cenoura* (1329), *R. solanacearum* (VW363), *X. phaseoli* pv *fuscans* (772), *X. campestris* pv *campestris* (828), *X. campestris* pv *campestris* (51), and *X. phaseoli* pv *phaseoli* (BRM25302) and four phytopathogenic fungi (*F. solani*, *M. phaseolina*, *R. solani*, and *S. sclerotiorum*) for any of the evaluated concentrations, ranging at 2–256 μg mL^–1^ (Figure 3C,D).

Although the system successfully activated the target genes, the experimental data indicate that the elevated gene expression did not translate to improved antimicrobial activity in the protein crude extracts from the transformed roots. In addition, previous studies support these findings; purified Pv-lectin extracts from *P. vulgaris* did not show antifungal or antibacterial activity.^40^ A similar result was detected in purified PvD1, which showed activity against yeast in a concentration of 100 μg mL^–1^ but was not evaluated against filamentous fungus or phytobacteria.^41^ Additionally, purified Cp-thionin II did not exhibit activity against phytopathogens *Ralstonia solanacearum*, *Rhataybacter sp.*, and *Erwinia* sp.,^42^ and a similar result was obtained in this study in the antimicrobial evaluation of Pv-thionin.

The absence of antimicrobial activity in the root extracts suggests that an increase in gene expression alone cannot induce an effective defense response. Other factors, such as the presence of post-translational modifications, the three-dimensional structure of the proteins, or the requirement for specific cofactors for biological activity, may play a critical role in the antimicrobial function of Pv-lectin, PvD1, and Pv-thionin. It is essential to highlight that the specificity of the sgRNAs was confirmed by the lack of off-target gene expression alterations, validating the precision of the employed gene activation strategy. However, in future studies, a more detailed analysis of protein levels and potential post-translational modifications in the transformed lines would be necessary to clarify the underlying reasons for the lack of antimicrobial activity, and it could help overcome these limitations and provide a better understanding of the application of the CRISPR/dCas9 strategy in modulating defense responses in *P. vulgaris*.

This study highlights several future opportunities, including the potential for expanding the CRISPR–dCas9–TV transcriptional activation approach to other major crop species.^61−63^ The success observed in *P. vulgaris* lays the foundation for applying this strategy to other crops, including maize, soybeans, and wheat, with future research focused on addressing the unique challenges associated with each species.^29^ Additionally, enhancing the efficiency of genetic activation represents a promising direction for future studies. Refining the CRISPR–dCas9–TV system, including adjusting dCas9 variants or investigating more potent transcriptional activators, could improve gene expression modulation.^63−65^ Moreover, validating the performance of genetically modified plants under field conditions is crucial to assessing the practical viability of the CRISPR–dCas9–TV strategy for boosting pathogen resistance in agricultural settings.^66^ Additionally, expanding the pathogen resistance testing to include a broader range of phytopathogens is essential for evaluating the full potential of this approach.

Nevertheless, there are significant limitations to the current study. While achieving target gene modulation was successful, the lack of significant antimicrobial activity in the crude protein extracts is a key limitation. This indicates that transcriptional activation of the genes did not yield detectable antimicrobial effects under the experimental conditions employed. Further studies are required to optimize the expression and functional activity of these peptides, possibly in more complex systems or under different environmental stress conditions. Another limitation lies in the narrow focus on a small set of defense-related genes. Future research could expand the scope to include genes involved in additional defense mechanisms.

## 4 Conclusions

The findings of this study demonstrate the successful modulation of gene expression for PvD1, Pv-lectin, and Pv-thionin using the CRISPR–dCas9–TV transcriptional activation system, resulting in significant upregulation in *P. vulgaris* hairy roots. The precision and absence of detectable off-target effects highlight the robustness and reliability of this approach. These results validate CRISPR–dCas9–TV as a powerful tool for targeted gene activation and offer a promising strategy for enhancing plant defense responses. Focusing on key defense-related genes, this study lays the groundwork for innovative and sustainable biotechnological solutions to improve crop resilience and combat phytopathogens. Future applications of this system could lead to broader adoption in agricultural settings, addressing global challenges in crop protection and food security.

## Supporting Information Available

pPZP_CRISPRa_PvD1 vector map (Supporting Figure 1); pPZP_CRISPRa_Pv-lectin vector map (Supporting Figure 2); pPZP_CRISPRa_Pv-thionin vector map (Supporting Figure 3); pPZP_CRISPRa vector map (Supporting Figure 4); development of *P. vulgaris* hairy roots at 5 and 10 days after induction, using different vectors (Supporting Figure 5); *in silico* characterization of target genes (Supporting Table 1); general information on primers for genomic regions encoding the dCas9 coupling site and sequences (Supporting Table 2); pPZP_CRISPRa_PvD1 sequence (Supporting File 1); pPZP_CRISPRa_Pv-lectin sequence (Supporting File 2); pPZP_CRISPRa_Pv-thionin sequence (Supporting File 3); and pPZP_CRISPRa sequence (Supporting File 4) (PDF)

## Notes

The CRISPR–dCas9–TV strategy has proven its efficacy in activating defense genes in *P. vulgaris* roots, marking a significant advancement in plant genomic engineering for crop protection.
