# Supplementary material for: Unlocking Nature’s Shield: The Promising Potential of CRISPRa in Amplifying Antimicrobial Peptide Expression in Common Bean (Phaseolus vulgaris L.)
Source: ACS Omega. 2025 Feb 6;10(6):5909–18. doi: 10.1021/acsomega.4c09817 (PMC11840611; doi:10.1021/acsomega.4c09817)
Supplement: Supplementary file 1 — ao4c09817_si_001.pdf [file ao4c09817_si_001.pdf]

Unlocking Nature's Shield: The Promising Potential of CRISPRa in Amplifying Antimicrobial Peptide Expression in Common Bean (*Phaseolus vulgaris* L.)

Mariana Rocha Maximiano[1, 2], Lucas José de Sousa[3,4], Gabriel Cidade Feitosa [1,2,3], Maria Eduarda Melo Lopes[1,5], Brisa Ortega[1,5], Raquel dos Santos Madeiro[1,5], Fabiano Touzdjian Pinheiro Kohlrausch Távora[4,6], Bruna Medeiros Pereira[4], Osmundo Brilhante de Oliveira Neto[4], Cirano José Ulhôa[7], Ana Cristina Miranda Brasileiro[4], Francisco José Lima Aragão[4], Angela Mehta*[4], Octávio Luiz Franco*[1,2]

1 - Universidade Católica de Brasília, Centro de Análises Proteômicas e Bioquímicas, Programa de Pós-Graduação em Ciências Genômicas e Biotecnologia, Brasília, CEP: 71966-700, Distrito Federal, Brazil.
2 - Universidade Católica Dom Bosco, S-Inova Biotech, Pós-Graduação em Biotecnologia, Campo Grande, CEP: 79117-900, Mato Grosso do Sul, Brazil.
3 - Universidade de Brasília, Brasília, CEP: 70910-900, Distrito Federal, Brazil.
4 - Embrapa Recursos Genéticos e Biotecnologia, Brasília, CEP: 70770-917, Distrito Federal, Brazil.
5- Centro Universitário do Distrito Federal, Brasília, CEP: 70390-030, Distrito Federal, Brazil.
6- Symbiomics, Florianópolis, CEP: 88050-000, Santa Catarina, Brazil.
7- Universidade Federal do Goiás, Goiânia, 74690-900, Goiás, Brazil.

*Corresponding author:

Octávio Luiz Franco
Universidade Católica de Brasília, Pós-graduação em Ciências Genômicas e Biotecnologia,
Campus Taguatinga QS 07, Lote 01, Sala G106 – EPCT – Taguatinga, Distrito Federal, Brasil.
CEP: 71966-700
Tel: +55 67 99854942
E-mail: ocfranco@gmail.com
Angela Mehta
Embrapa Recursos Genéticos e Biotecnologia, PBI, Av. W/5 Norte Final
CEP 70770-917, Brasília, DF, Brazil
e-mail: angela.mehta@embrapa.br

Supplementary figures and captions

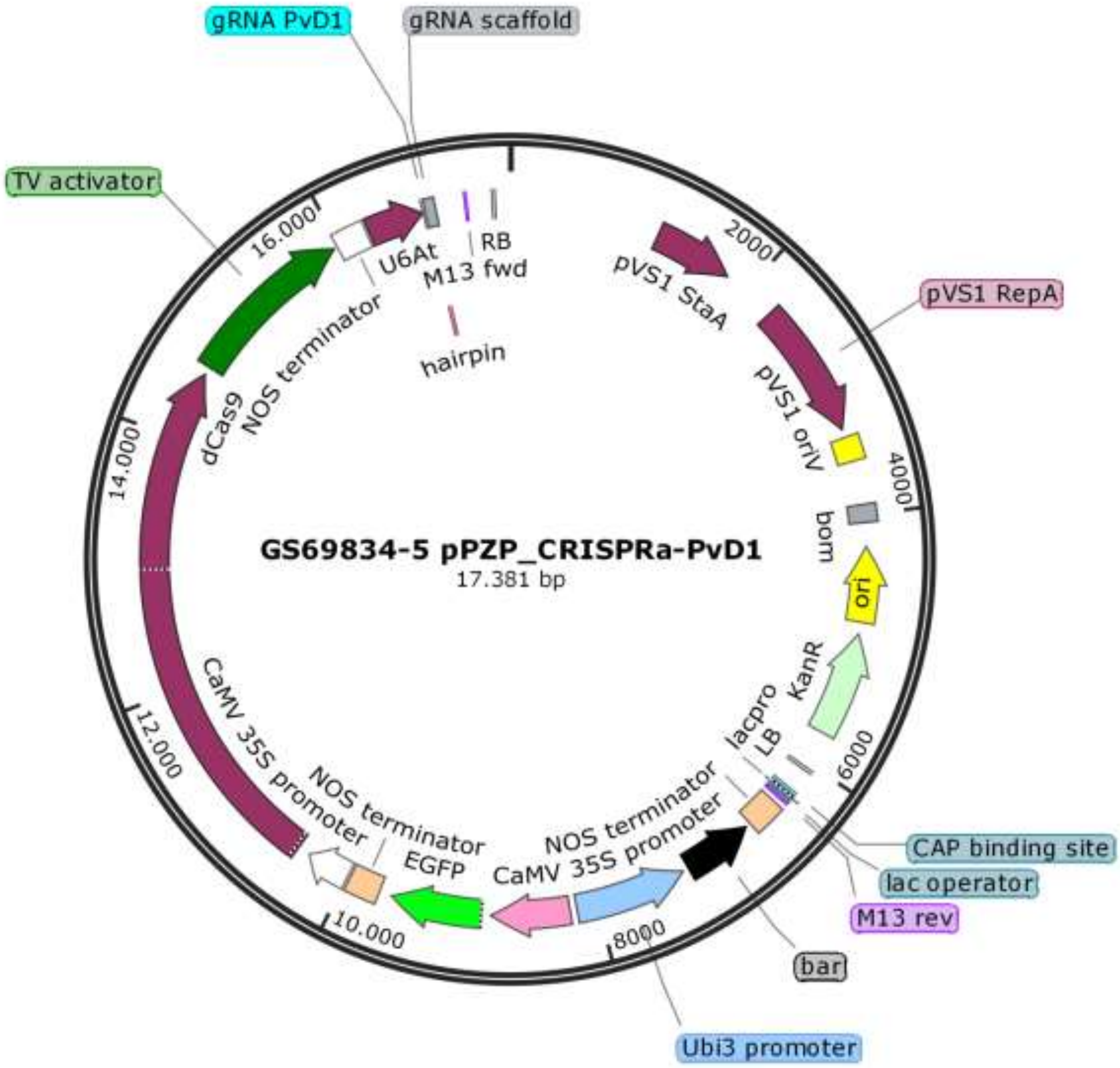

Supplementary Figure 1. pPZP_CRISPRa_PvD1 vector map. **EGFP -** GFP Gene reporter; **dCas9** – dCas9 gene; **TV activator –** 6X TAL fused in tandem to VP128 activation.

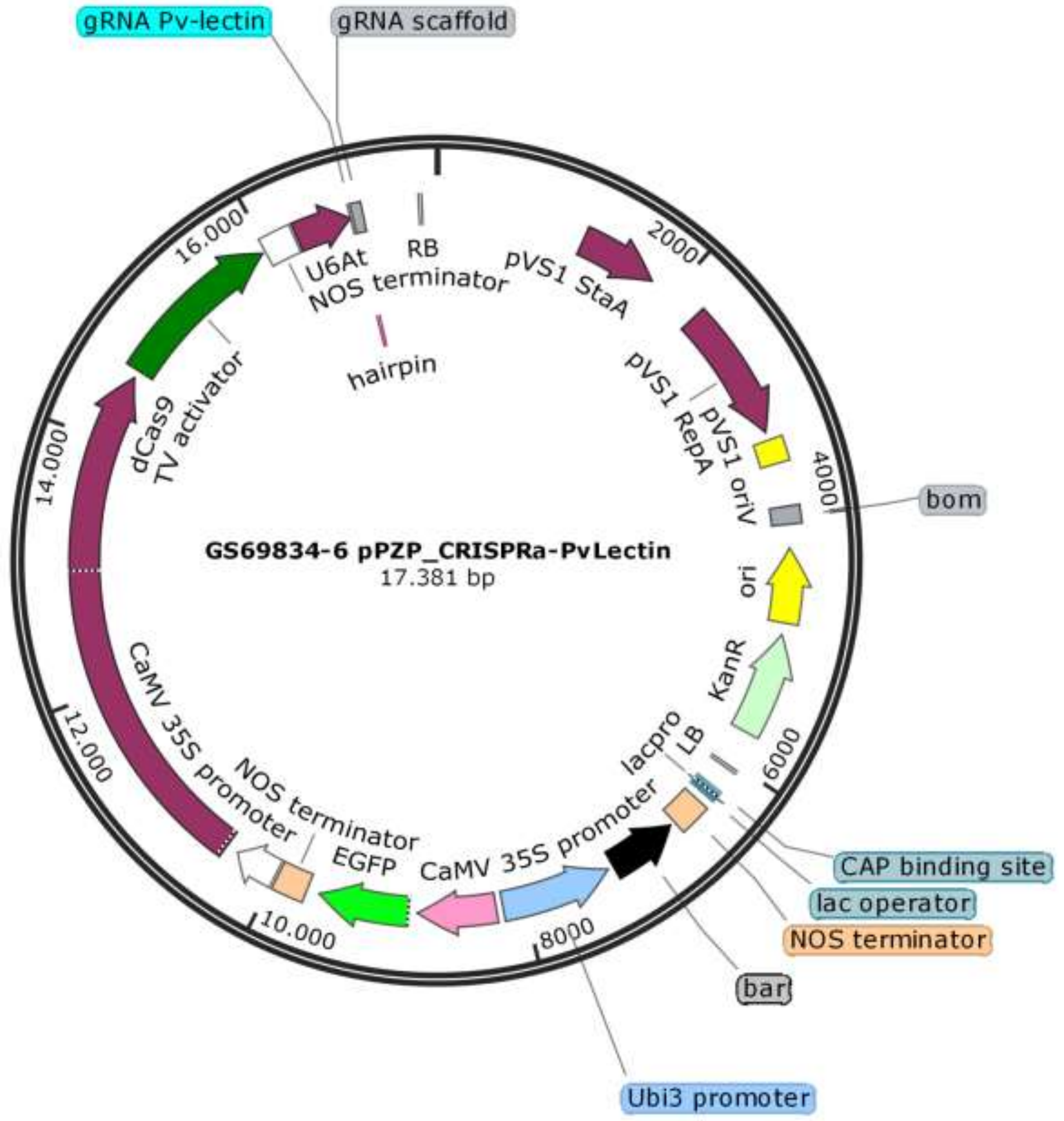

Supplementary Figure 2. pPZP_CRISPRa_Pv-lectin vector map. **EGFP** - GFP Gene reporter; **dCas9** – dCas9 gene; **TV activator –** 6X TAL fused in tandem to VP128 activation.

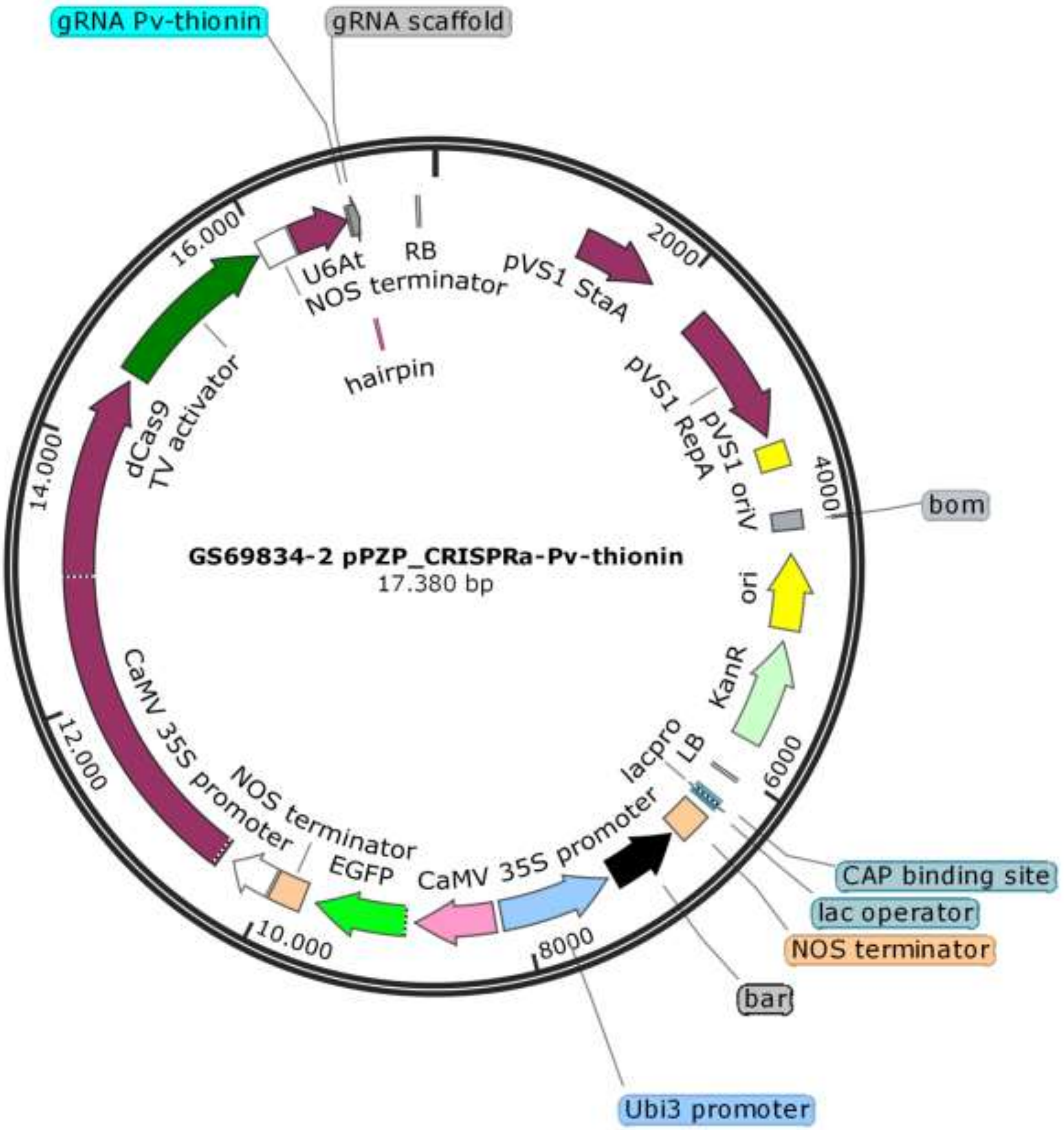

Supplementary Figure 3. pPZP_CRISPRa_Pv-thionin vector map. **EGFP** - GFP Gene reporter; **dCas9** – dCas9 gene; **TV activator –** 6X TAL fused in tandem to VP128 activation.

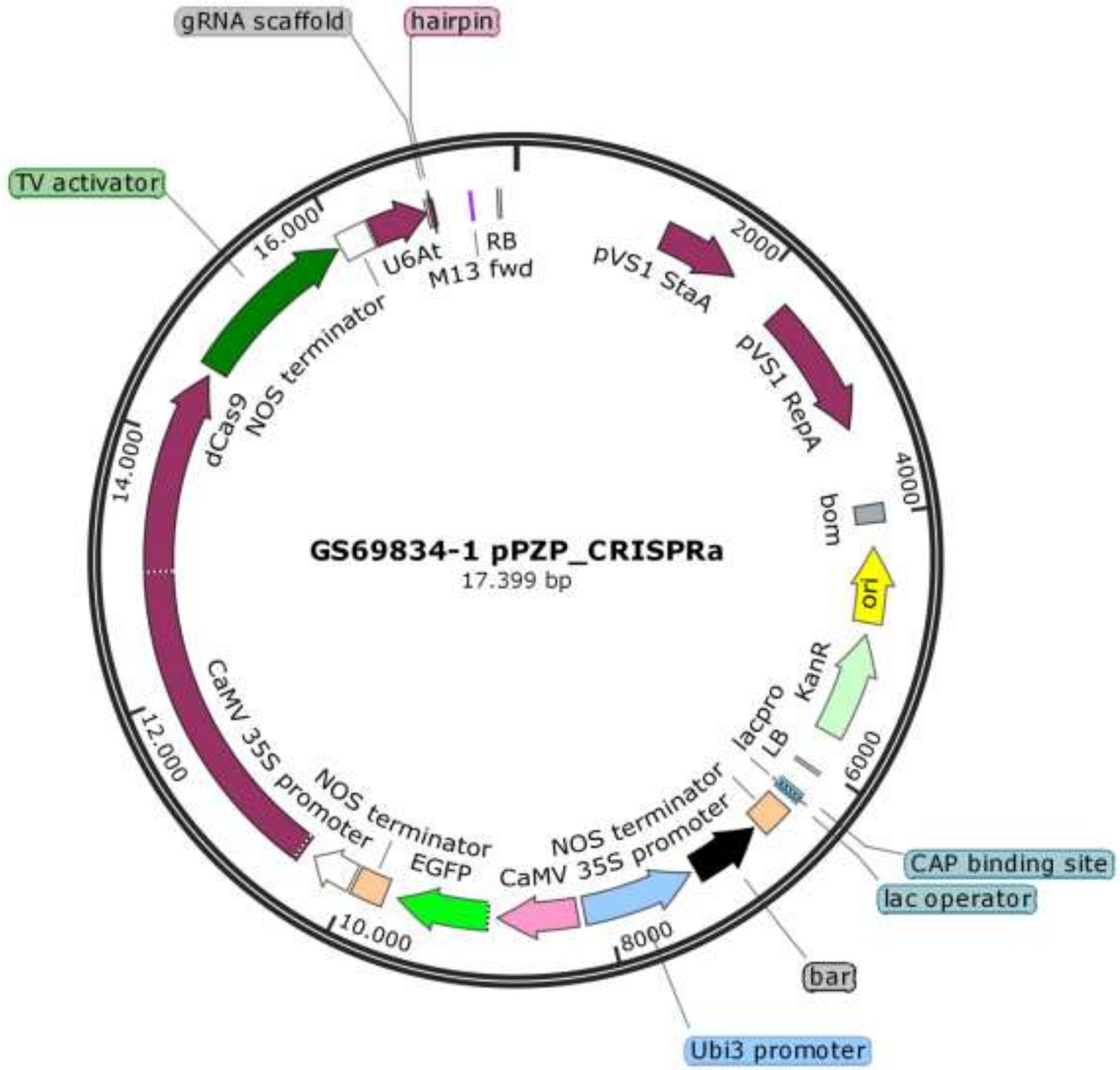

Supplementary Figure 4. pPZP_CRISPRa vector map. **EGFP** - GFP Gene reporter; **dCas9** – dCas9 gene; **TV activator –** 6X TAL fused in tandem to VP128 activation.

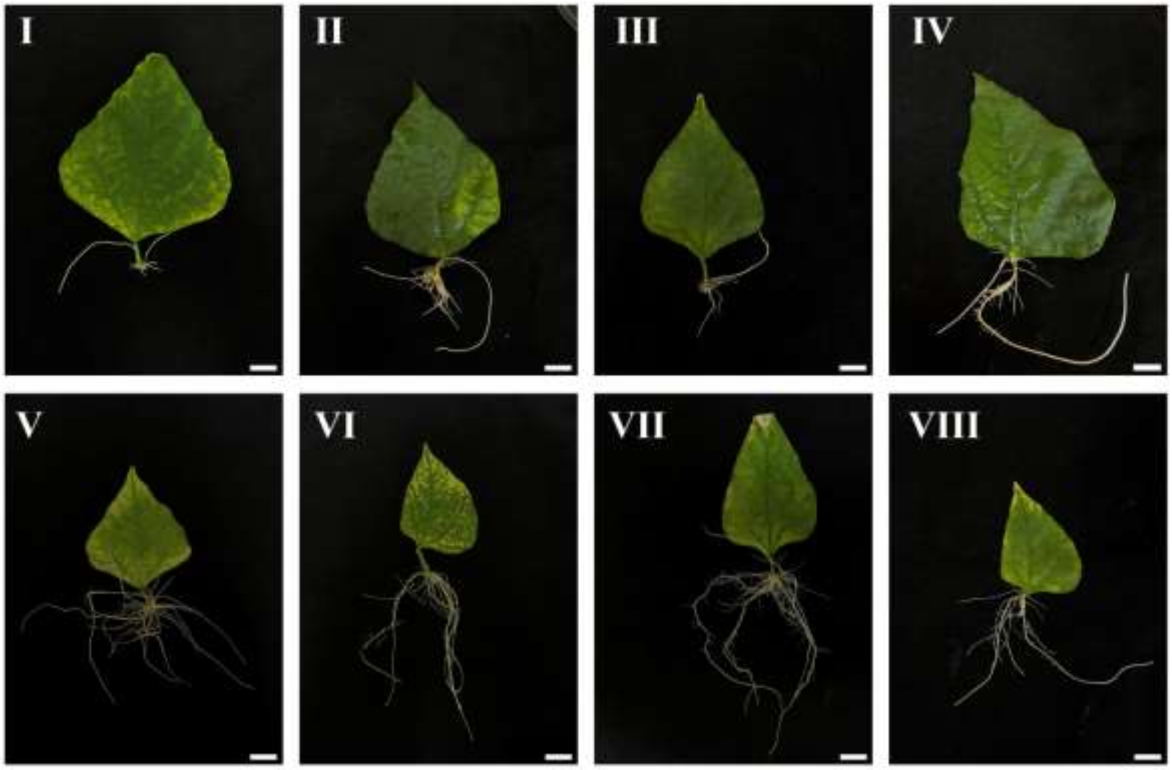

Supplementary Figure 5. *Phaseolus vulgaris* hairy roots development at 5 days (I, II, III, IV) and 10 days (V, VI, VII, VIII) after hairy roots induction. Bars represent 1 cm.

Supplementary Table 1. *In silico* characterization of target genes. Codon transcription initiation (yellow), TATA box region (green) guide RNA (blue).

Gene	Sequence ID	Gene ID	Chromosome localization	Sequence (5'→3')
**PvD1**	XM_007149386.1	PHAVU_005G071300g	PhaVulg1_0:5:11843212:11844487:1	AAATGTTAAAGCATATATAGTTATTCCGTCACAAATTCCGGCTAGAAGCCTAGCATTTAATATCATGATAAGTTCCTTTCCTATTTTGGCACATTGT<br>TGACTTTTCACACCATCAAAATCCAAACCACCGAAAACCTCCTTACATCAAAAGACAAATCCACCGAAACCTCCTTACATCAAAATACAAATCCACC<br>GAAAGCTCCTTACAGATTCATTCATTAGTTTCTTGTAGTTTCTACTTAGTTTCTCATCAGCAACCTCATCATTGTTTACTTTTCAACTCCTCGCTAC<br>TATATATATGTTTAATAAACACAAACCAGTCATCACACACACAGAGCAGATACATACAAGTGAATATTCACTAACTAAGTCATGGAGAAGAAATCAT<br>TAGCTGGGTTATGCTTCCTCTTCCTTGTTCTCTTTGTTACTCGTAAGTCACTACTTTTATGTTCATTCTTCTACATGTCTATAACTATATTCATTCT<br>CCTGAATACTTTATAATTCAATATGTCTGATATTTACTGTTGAATTTATTTGAACATGCAGAAGAAGTTGTGTTGCAGACTGAGGCAAAGACTTGCG<br>AGAACCTGGCTGATACATACAAGGGTCCATGCTTCACCACTGGCAGCTGCGATGATCACTGCAAGAACAAAGAACACTTGAGGAGTGGCAGGTGCAG<br>GGATGATTTCCGCTGTTGGTGCACCAAAAACTGTTAAGTGGATTCAGAACTTTTTATAAATAAAACAACTATTATATATATGGAATAAGACTAGCAC<br>TGCATCCACGAACTATATGTATAGTCTGTGGCTGTGCAGAACAGCTGAGTTATGTATTTACTTTATGAATAAAGTTCTTCTGTTGTAATACATGATC<br>TTATCCTTCTTCGTACCATCATTTAATCTTTTTCTCACATATTTTCTGTTGTTCTTCTGAAAGGAACTTGATGTATATATGAAAAGGAATTAGTTA<br>ATTGATTTAATACTTTGTTCATCAAAATAACTAATCTTTTTATGTACAAAACTTTTAAACGTCCTAAATGAGTCTCTATTGTTCACTGATAAAAAAA<br>AATAGTGAAAGCAAAGAAATAGTAATTTATAATACTTTTAAAAAAGAATATTACAGGTTTATAATAGGGTATTGGTTCACTACAATAATTATCGATA<br>TTTTATTTTAAATATAATAAAAAAGTTACTATTTTAATAAAATATTTTAATTTTGATAATTTTGTTTTAATATAAAAATATATTTTTAACAAAAAAA<br>ATTAATTTTAATAAT
**Pv-lectin**	XM_007152710.1	PHAVU_004G158200g	PhaVulg1_0:4:44022956:44024416:-1	TCTTCACCCGTGTTTTCCAATGCTACGTGCCAACCGCTTCTCTCTATAAATATCTCTTTAAATTTAAACTAATTATTTCATATTTTTTCAATGTATT<br>TGATGACGTGTATGCATTGCCATCGTTGCTTAATTGTTATTTCTATATTCTTATTACTCCCTCAAATAATATTATAAAAGAAAAAAGAATCCATAAA<br>GTAGAGAGAGAGAGTGCAGTTGTTGTTGTATAAATAGAGAAGAGAGTGATGGTTAATGCATGAATGCATACATGGCTTCCTCCAACTTACTCTCCCT<br>AGCCCTCTTCCTTGTGCTTCTCACCCACGCAAACTCAGCCAGCCAAACCTCCTTCAGCTTCCAAAGGTTCAACGAAACCAACCTTATCCTCCAACGC<br>GATGCCACCGTCTCATCCAAAGGCCAGTTACGACTAACCAATGTTAATGACAACGGAGAACCCACGTTGAGCTCTCTGGGCCGTGCCTTCTACTCCG<br>CCCCCATCCAAATCTGGGACAACACCACCGGCGCCGTGGCCAGCTTCGCCACCTCCTTCACATTCAATATCGACGTTCCCAACAATTCAGGACCCGC<br>CGATGGCCTTGCCTTTGTTCTCCTCCCCGTGGGCTCTCAGCCCAAAGACAAAGGCGGTCTTCTAGGTCTGTTCAACAACTACAAATACGACAGCAAT<br>GCCCATACTGTGGCTGTGGAGTTCGACACCCTCTACAACGTTCACTGGGACCCCAAACCGCGTCATATTGGCATCGACGTGAACTCCATCAAGTCTA<br>TCAAAACGACGACGTGGGATTTTGTCAAAGGAGAAAACGCGGAGGTTCTGATCACCTATGACTCCTCCACGAAGCTCTTGGTGGCTTCTCTGGTTTA<br>CCCTTCTCTGAAAACAAGCTTCATCGTCTCTGACACAGTGGACCTGAAGAGCGTTCTTCCCGAGTGGGTGATCGTTGGGTTCACTGCCACCACTGGG<br>ATTACTAAAGGGAACGTTGAAACGAACGACATCCTCTCTTGGTCTTTTGCTTCCAAGCTCTCCGATGGCACCACATCTGAAGCTTTGAATCTTGGCA<br>ACTTCGCCCTCAACCAAATCCTCTAGACTCAATCTCCACCTTCCCTGTGACACTAAATCACTGCTTACACTCAGTGTTCTTTCCTGCTAATAATGTT<br>TCTCTTTGTCTGTCACAGAAAATAAAATAAAATAGGGTGATGATAGCTTACACTCAGTGTTCTTTGCTCTACTTCCTTAATTCCTTAATGAAAATAT<br>AATAAAAAATAATAATTAAAATATTAATATTTATTGGGTTAAAGGTAAATACGAGTGTTAAAAAATCATTTTTAAATAAAATAAATAAAATGGAAGT<br>TCATATATTATTATATTATTAAAAGGAGCTGTCAAATGTTTGTTTTTTACTCCAAACTTTGGACTGTTAAAGAAAAGTTTTCCAAAATAATACCATC<br>ATGGAG
**Pv-thionin**	XM_007159854.1	PHAVU_002G278400g	PhaVulg1_0:2:44271869:44273036:1	AGCACACCTTACAATGGCACCGATGTGTGTGGTCATTCTGTTGTTCTCCCATTGGA**CCT**GCGTTTCCACAAACCTTATCGTGGCAGCTTACAACCCT<br>TCTCTACGCACACGTAGTTCCTTTGCATGTCACACTTATCTTCCACCTCTTACCCATTCTCTCCCACATTCACTCTATATAACAACATCTCAACACC<br>CACACTTTCTTCACTTCCACCTCAACAATTCATCACTC**ATG**GAGAGGAAAACACTCAGCTTCTTGTTCTTGTTCCTCCTTGTTTTAGCTTCTGGTAA<br>GATGCATATATAGTTCCTTGCAATATATACATGTGTGTGACGAACTCTTGATTAACCATTTGAGTGATCAATGTGAAATCAGATGTGGCGGTGAAGA<br>AAGCAGAAGCGAAAACTTGCTTCACAAAGAAGGAGGGGTGGGGAAGATGCATAGCTGATTTCACCTGTGCACGTTCATGCAGGAATGTAGGCTACAT<br>CGGAGGCAAGTGCCAAGGCCTCACTCGCCGCTGCTTTTGCGTACTGAACTGTTGAAGTTAACAACAAAACCCTTTTCATATATCATATTATGTGATC<br>ATAAATAAATATAGCACCACCACTACTAGTACTATGCCTATGTAGAATGGGGTTGGGGTGCTTAATTAGTGGCTAAGTAGTTATATTTATGGCCATC<br>ATATATATCTATATGGCCTGCATGTGTCTTTCATTAACGCAGTGTGGCGAGCAAGTATGATGTTGTGATCTAGTTATGGCTCCATCTTCTTGTTCTG<br>CTGATGGTGTAATCAGTTTTATCTGTGGTTTATATATGTAATGGAAATAAGTTTTGCATTGCGGTGCCTTTTTCTCCATGGATGAAGTGAGAAATGG<br>TGGGTTTTGCTGCCCTCCATTGATAAGCTTCTGAATTGTGTTGTACATGCAGACATGATCGAATACTTAGACTTCAAAATAGAGTAGTTTGTACAAA<br>AGAAAATGAATCTTCACTTTAAGTTCAGATGGAACAGCTATGGGGTTTCAAAATCGTGTCTTCTGAAGTTTAGACACAACAGACATATTCTCCGCTA<br>TATTATTGAATAAGTAATAGCATCAAATTTCTCACTTAAAGAGGAAATACTGATACTAAAATTAAAATTTTAAAATTAATAAGGGTAATTTGGTCTC<br>AAT

Supplementary table 2. General information on primers for the genomic region that encoded the dCas9 coupling site and dCas9 sequences.

Identification	Forward Primer (5' → 3')	Tm °C	Reverse Primer (5' → 3')	Tm °C	Amplicon
**Target site of PvD1**	AGCATATATAGTTATTCCGTCACAA	57.8	TTTGCCTCAGTCTGCAACAC	58.9	564
**The target site of Pv-lectin**	GGTTTGGCTGGCTGAGTTTG	57.1	CTCAGCTCGCTCCTCTTCAC	57.5	512
**The target site of Pv-thionin**	ACACAGTGTAGAACAAAGCTCCA	56.4	GCTTCTGCTTTCTTCACCGC	57.0	542
**dCas9**	TTACCGTGAAACAGCTCAAA	52.2	CTTCTTCTGAGGGGACGTTA	53.6	948

Supplementary File 1. pPZP_CRISPRa_PvD1 sequence.

5’AGTACTTTGATCCAACCCCTCCGCTGCTATAGTGCAGTCGGCTTCTGACGTTCAGTGCAGCCG
TCTTCTGAAAACGACATGTCGCACAAGTCCTAAGTTACGCGACAGGCTGCCGCCCTGCCCTTTTC
CTGGCGTTTTCTTGTCGCGTGTTTTAGTCGCATAAAGTAGAATACTTGCGACTAGAACCGGAGAC
ATTACGCCATGAACAAGAGCGCCGCCGCTGGCCTGCTGGGCTATGCCCGCGTCAGCACCGACGAC
CAGGACTTGACCAACCAACGGGCCGAACTGCACGCGGCCGGCTGCACCAAGCTGTTTTCCGAGAA
GATCACCGGCACCAGGCGCGACCGCCCGGAGCTGGCCAGGATGCTTGACCACCTACGCCCTGGCG
ACGTTGTGACAGTGACCAGGCTAGACCGCCTGGCCCGCAGCACCCGCGACCTACTGGACATTGCC
GAGCGCATCCAGGAGGCCGGCGCGGGCCTGCGTAGCCTGGCAGAGCCGTGGGCCGACACCACCAC
GCCGGCCGGCCGCATGGTGTTGACCGTGTTCGCCGGCATTGCCGAGTTCGAGCGTTCCCTAATCA
TCGACCGCACCCGGAGCGGGCGCGAGGCCGCCAAGGCCCGAGGCGTGAAGTTTGGCCCCCGCCCT
ACCCTCACCCCGGCACAGATCGCGCACGCCCGCGAGCTGATCGACCAGGAAGGCCGCACCGTGAA
AGAGGCGGCTGCACTGCTTGGCGTGCATCGCTCGACCCTGTACCGCGCACTTGAGCGCAGCGAGG
AAGTGACGCCCACCGAGGCCAGGCGGCGCGGTGCCTTCCGTGAGGACGCATTGACCGAGGCCGAC
GCCCTGGCGGCCGCCGAGAATGAACGCCAAGAGGAACAAGCATGAAACCGCACCAGGACGGCCAG
GACGAACCGTTTTTCATTACCGAAGAGATCGAGGCGGAGATGATCGCGGCCGGGTACGTGTTCGA
GCCGCCCGCGCACGTCTCAACCGTGCGGCTGCATGAAATCCTGGCCGGTTTGTCTGATGCCAAGC
TGGCGGCCTGGCCGGCCAGCTTGGCCGCTGAAGAAACCGAGCGCCGCCGTCTAAAAAGGTGATGT
GTATTTGAGTAAAACAGCTTGCGTCATGCGGTCGCTGCGTATATGATGCGATGAGTAAATAAACA
AATACGCAAGGGGAACGCATGAAGGTTATCGCTGTACTTAACCAGAAAGGCGGGTCAGGCAAGAC
GACCATCGCAACCCATCTAGCCCGCGCCCTGCAACTCGCCGGGGCCGATGTTCTGTTAGTCGATT
CCGATCCCCAGGGCAGTGCCCGCGATTGGGCGGCCGTGCGGGAAGATCAACCGCTAACCGTTGTC
GGCATCGACCGCCCGACGATTGACCGCGACGTGAAGGCCATCGGCCGGCGCGACTTCGTAGTGAT
CGACGGAGCGCCCCAGGCGGCGGACTTGGCTGTGTCCGCGATCAAGGCAGCCGACTTCGTGCTGA
TTCCGGTGCAGCCAAGCCCTTACGACATATGGGCCACCGCCGACCTGGTGGAGCTGGTTAAGCAG
CGCATTGAGGTCACGGATGGAAGGCTACAAGCGGCCTTTGTCGTGTCGCGGGCGATCAAAGGCAC
GCGCATCGGCGGTGAGGTTGCCGAGGCGCTGGCCGGGTACGAGCTGCCATTCTTGAGTCCCGTAT
CACGCAGCGCGTGAGCTACCCAGGCACTGCCGCCGCCGGCACAACCGTTCTTGAATCAGAACCCG
AGGGCGACGCTGCCCGCGAGGTCCAGGCGCTGGCCGCTGAAATTAAATCAAAACTCATTTGAGTT
AATGAGGTAAAGAGAAAATGAGCAAAAGCACAAACACGCTAAGTGCCGGCCGTCCGAGCGCACGC
AGCAGCAAGGCTGCAACGTTGGCCAGCCTGGCAGACACGCCAGCCATGAAGCGGGTCAACTTTCA
GTTGCCGGCGGAGGATCACACCAAGCTGAAGATGTACGCGGTACGCCAAGGCAAGACCATTACCG
AGCTGCTATCTGAATACATCGCGCAGCTACCAGAGTAAATGAGCAAATGAATAAATGAGTAGATG
AATTTTAGCGGCTAAAGGAGGCGGCATGGAAAATCAAGAACAACCAGGCACCGACGCCGTGGAAT
GCCCCATGTGTGGAGGAACGGGCGGTTGGCCAGGCGTAAGCGGCTGGGTTGTCTGCCGCCCTGCA
ATGGCACTGGAACCCCCAAGCCCGAGGAATCGGCGTGACGGTCGCAAACCATCCGGCCCGGTACA
AATCGGCGCGGCGCTGGGTGATGACCTGGTGGAGAAGTTGAAGGCCGCGCAGGCCGCCCAGCGGC
AACGCATCGAGGCAGAAGCACGCCCCGGTGAATCGTGGCAAGCGGCCGCTGATCGAATCCGCAAA
GAATCCCGGCAACCGCCGGCAGCCGGTGCGCCGTCGATTAGGAAGCCGCCCAAGGGCGACGAGCA

ACCAGATTTTTTCGTTCCGATGCTCTATGACGTGGGCACCCGCGATAGTCGCAGCATCATGGACG
TGGCCGTTTTCCGTCTGTCGAAGCGTGACCGACGAGCTGGCGAGGTGATCCGCTACGAGCTTCCA
GACGGGCACGTAGAGGTTTCCGCAGGGCCGGCCGGCATGGCCAGTGTGTGGGATTACGACCTGGT
ACTGATGGCGGTTTCCCATCTAACCGAATCCATGAACCGATACCGGGAAGGGAAGGGAGACAAGC
CCGGCCGCGTGTTCCGTCCACACGTTGCGGACGTACTCAAGTTCTGCCGGCGAGCCGATGGCGGA
AAGCAGAAAGACGACCTGGTAGAAACCTGCATTCGGTTAAACACCACGCACGTTGCCATGCAGCG
TACGAAGAAGGCCAAGAACGGCCGCCTGGTGACGGTATCCGAGGGTGAAGCCTTGATTAGCCGCT
ACAAGATCGTAAAGAGCGAAACCGGGCGGCCGGAGTACATCGAGATCGAGCTAGCTGATTGGATG
TACCGCGAGATCACAGAAGGCAAGAACCCGGACGTGCTGACGGTTCACCCCGATTACTTTTTGAT
CGATCCCGGCATCGGCCGTTTTCTCTACCGCCTGGCACGCCGCGCCGCAGGCAAGGCAGAGCCAG
ATGGTTGTTCAAGACGATCTACGAACGCAGTGGCAGCGCCGGAGAGTTCAAGAAGTTCTGTTTCA
CCGTGCGCAAGCTGATCGGGTCAAATGACCTGCCGGAGTACGATTTGAAGGAGGAGGCGGGGCAG
GCTGGCCCGATCCTAGTATGCGCTACCGCAACCTGATCGAGGGCGAAGCATCCGCCGGTTCCTAA
TGTACGGAGCAGATGCTAGGGCAAATTGCCTAGCAGGGGAAAAAGGTCGAAAAGGTCTCTTTCCT
GTGGATAGCACGTACATTGGGAACCCAAAGCCGTACATTGGAACCGGAACCCGTACATTGGGAAC
CCAAAGCCGTACATTGGGAACCGGTCACACATGTAAGTGACTGATATAAAGAGAAAAAAGGCGAT
TTTTCCGCCTAAAACTCTTTAAAACTTATTAAAACTCTTAAAACCCGCCTGGCCTGTGCATAACT
GTCTGGCCAGCGCACAGCCGAAGAGCTGCAAAAAGCGCCTACCCTTCGGTCGCTGCGCTCCCTAC
GCCCCGCGCTTCGCGTCGGCCTATCGCGGCCGCTGGCCGCTCAAAAATGGCTGGCCTACGGCCAG
GCAATCTACCAGGGCGCGACAAGCCGCGCCGTCGCCACTCGACCGCCGGCGCCCACATCAAGGCA
CCCTGCCTCGCGCGTTTCGGTGATGACGGTGAAAACCTCTGACACATGCAGCTCCCGGAGACGGT
CACAGCTTGTCTGTAAGCGGATGCCGGGAGCAGACAAGCCCGTCAGGGCGCGTCAGCGGGTGTTG
GCGGGTGTCGGGGCGCAGCCATGACCCAGTCACGTAGCGATAGCGGAGTGTATACTGGCTTAACT
ATGCGGCATCAGAGCAGATTGTACTGAGAGTGCACCATATGCGGTGTGAAATACCGCACAGATGC
GTAAGGAGAAAATACCGCATCAGGCGCTCTTCCGCTTCCTCGCTCACTGACTCGCTGCGCTCGGT
CGTTCGGCTGCGGCGAGCGGTATCAGCTCACTCAAAGGCGGTAATACGGTTATCCACAGAATCAG
GGGATAACGCAGGAAAGAACATGTGAGCAAAAGGCCAGCAAAAGGCCAGGAACCGTAAAAAGGCC
GCGTTGCTGGCGTTTTTCCATAGGCTCCGCCCCCCTGACGAGCATCACAAAAATCGACGCTCAAG
TCAGAGGTGGCGAAACCCGACAGGACTATAAAGATACCAGGCGTTTCCCCCTGGAAGCTCCCTCG
TGCGCTCTCCTGTTCCGACCCTGCCGCTTACCGGATACCTGTCCGCCTTTCTCCCTTCGGGAAGC
GTGGCGCTTTCTCATAGCTCACGCTGTAGGTATCTCAGTTCGGTGTAGGTCGTTCGCTCCAAGCT
GGGCTGTGTGCACGAACCCCCCGTTCAGCCCGACCGCTGCGCCTTATCCGGTAACTATCGTCTTG
AGTCCAACCCGGTAAGACACGACTTATCGCCACTGGCAGCAGCCACTGGTAACAGGATTAGCAGA
GCGAGGTATGTAGGCGGTGCTACAGAGTTCTTGAAGTGGTGGCCTAACTACGGCTACACTAGAAG
GACAGTATTTGGTATCTGCGCTCTGCTGAAGCCAGTTACCTTCGGAAAAAGAGTTGGTAGCTCTT
GATCCGGCAAACAAACCACCGCTGGTAGCGGTGGTTTTTTTGTTTGCAAGCAGCAGATTACGCGC
AGAAAAAAAGGATCTCAAGAAGATCCTTTGATCTTTTCTACGGGGTCTGACGCTCAGTGGAACGA
AAACTCACGTTAAGGGATTTTGGTCATGCAGGATCATGAATTAATTCTTAGAAAAACTCATCGAG
CATCAAATGAAACTGCAATTTATTCATATCAGGATTATCAATACCATATTTTTGAAAAAGCCGTT

TCTGTAATGAAGGAGAAAACTCACCGAGGCAGTTCCATAGGATGGCAAGATCCTGGTATCGGTCT
GCGATTCCGACTCGTCCAACATCAATACAACCTATTAATTTCCCCTCGTCAAAAATAAGGTTATC
AAGTGAGAAATCACCATGAGTGACGACTGAATCCGGTGAGAATGGCAAAAGTTTATGCATTTCTT
TCCAGACTTGTTCAACAGGCCAGCCATTACGCTCGTCATCAAAATCACTCGCATCAACCAAACCG
TTATTCATTCGTGATTGCGCCTGAGCGAGACGAAATACGCGATCGCTGTTAAAAGGACAATTACA
AACAGGAATCGAATGCAACCGGCGCAGGAACACTGCCAGCGCATCAACAATATTTTCACCTGAAT
CAGGATATTCTTCTAATACCTGGAATGCTGTTTTCCCGGGGATCGCAGTGGTGAGTAACCATGCA
TCATCAGGAGTACGGATAAAATGCTTGATGGTCGGAAGAGGCATAAATTCCGTCAGCCAGTTTAG
TCTGACCATCTCATCTGTAACATCATTGGCAACGCTACCTTTGCCATGTTTCAGAAACAACTCTG
GCGCATCGGGCTTCCCATACAATCGATAGATTGTCGCACCTGATTGCCCGACATTATCGCGAGCC
CATTTATACCCATATAAATCAGCATCCATGTTGGAATTTAATCGCGGCCTAGAGCAAGACGTTTC
CCGTTGAATATGGCTCATAACACCCCTTGTATTACTGTTTATGTAAGCAGACAGTTTTATTGTTC
ATGATCTGGATCACAGGCAGCAACGCTCTGTCATCGTTACAATCAACATGCTACCCTCCGCGAGA
TCATCCGTGTTTCAAACCCGGCAGCTTAGTTGCCGTTCTTCCGAATAGCATCGGTAACATGAGCA
AAGTCTGCCGCCTTACAACGGCTCTCCCGCTGACGCCGTCCCGGACTGATGGGCTGCCTGTATCG
AGTGGTGATTTTGTGCCGAGCTGCCGGTCGGGGAGCTGTTGGCTGGCTGGTGGCAGGATATATTG
TGGTGTAAACAAATTGACGCTTAGACAACTTAATAACACATTGCGGACGTTTTTAATGTACTGAA
TTAACGCCGAATTGCTCTAGCCAATACGCAAACCGCCTCTCCCCGCGCGTTGGCCGATTCATTAA
TGCAGCTGGCACGACAGGTTTCCCGACTGGAAAGCGGGCAGTGAGCGCAACGCAATTAATGTGAG
TTAGCTCACTCATTAGGCACCCCAGGCTTTACACTTTATGCTTCCGGCTCGTATGTTGTGTGGAA
TTGTGAGCGGATAACAATTTCACACAGGAAACAGCTATGACATGATTACGAATTCTTAATTAAGC
TACCCGGGGATCAATTCCCGATCTAGTAACATAGATGACACCGCGCGCGATAATTTATCCTAGTT
TGCGCGCTATATTTTGTTTTCTATCGCGTATTAAATGTATAATTGCGGGACTCTAATCATAAAAA
CCCATCTCATAAATAACGTCATGCATTACATGTTAATTATTACATGCTTAACGTAATTCAACAGA
AATTATATGATAATCATCGCAAGACCGGCAACAGGATTCAATCTTAAGAAACTTTATTGCCAAAT
GTTTGAACGATCGGGGAAATTCGAGCTTCGTGGATCCCGGTCGGCATCTACTTCAAATCTCGGTG
ACGGGCAGGACCGGACGGGGCGGTACCGGCAGGCTGAAGTCCAGCTGCCAGAAACCCACGTCATG
CCAGTTCCCGTGCTTGAAGCCGGCCGCCCGCAGCATGCCGCGGGGGGCATATCCGAGCGCCTCGT
GCATGCGCACGCTCGGGTCGTTGGGCAGCCCGATGACAGCGACCACGCTCTTGAAGCCCTGTGCC
TCCAGGGACTTCAGCAGGTGGGTGTAGAGCGTGGAGCCCAGTCCCGTCCGCTGGTGGCGGGGGA
GACGTACACGGTCGACTCGGCCGTCCAGTCGTAGGCGTTGCGTGCCTTCCAGGGGCCCGCGTAGG
CGATGCCGGCGACCTCGCCGTCCACCTCGGCGACGAGCCAGGGATAGCGCTCCCGCAGACGGACG
AGGTCGTCCGTCCACTCCTGCGGTTCCTGCGGCTCGGTACGGAAGTTGACCGTGCTTGTCTCGAT
GTAGTGGTTGACGATGGTGCAGACCGCCGGCATGTCCGCCTCGGTGGCACGGCGGATGTCGGCCG
GGCGTCGTTCTGGGCTCATATCTTATTGCCCCCCTAGAGTCGAGATCCTTCGCCTGGAGGAGAGA
AATCAGTGGCGCTGCGGCTTTTAGGGTTTCTTTGTTGATGGAATGAGAGTGTAAGCTCTGCCAGT
GCCACTTTATTAGGGTTTTACAAGCCCTTTTCTTCGTAATTGGGCCTGACATTTTGTGCCACTTG
GGCCTTTAGAGATGAAAATGTATATTGGGCTTAAGTTGACTTGAAGGATAAATTAGTTTAGGATA
TTACGTTTTTTATGAGAATTGGTGTGTCGGATACATGTATATGATGCATTCAAATATATGTATTC

TAGATACATTTAAGTTTAGATACAATCTAAAATGTGTCTTTAATTACAGGACTGTAACTAAAATA
CTTAATGTAAGAAGAATATTACTCCTTTAATAGCTTTTGAGTATATCTAGTCTAACATCTTTTAA
AAAAGTCTAATTTCTTTCATTTATTTTTCGAGCAATAGCAAAGTGCATAATTATTTTTTTCTTCT
AGAAATTCAGATTTGTTTCTCTAAATTTTGAGATTCTTTTCTCAATTTTGTATGTCTAGAGAACA
ATGTGTATTTTTCACTCTAGTTGGTTGTTGCTTTGTTGAATGTTCTGATAAAAGTATATTGTTAT
TTCTGAAGTAGATATAAACCTTCATTTGGAAATTATACATAAATCAAAATCGTTAATTATCTAGA
TCAAGATATATGCCCTTTTCCTAATGTATTTGATACATGCACCTAATTTCACTAGATGTATCTTT
TCTATTTTTTAAATTATGAATAGTTAATTTTTTCCATATGTGTATTTGATACATACTTCATGACT
TTAAAAAATTAATTATATACCAGATATATGTATTTAAAATTTGTTATGTATTTAAAGTATGTATA
TGATTATTCGATATTAATCTCTTCGATGAAATTTAAATCGATAACTATGTGCTTTGGATCTGCCC
ACTATCGAGCTCAAGCTTGCATGCCTGCAGGTCCGATCTGAGACTTTTCAACAAAGGGTAATATC
GGGAAACCTCCTCGGATTCCATTGCCCAGCTATCTGTCACTTCATCAAAAGGACAGTAGAAAAGG
AAGGTGGCACCTACAAATGCCATCATTGCGATAAAGGAAAGGCTATCGTTCAAGATGCCTCTGCC
GACAGTGGTCCCAAAGATGGACCCCCACCCACGAGGAGCATCGTGGAAAAAGAAGACGTTCCAAC
CACGTCTTCAAAGCAAGTGGATTGATGTGATGGTCCGATTGAGACTTTTCAACAAAGGGTAATAT
CGGGAAACCTCCTCGGATTCCATTGCCCAGCTATCTGTCACTTCATCAAAAGGACAGTAGAAAAG
GAAGGTGGCACCTACAAATGCCATCATTGCGATAAAGGAAAGGCTATCGTTCAAGATGCCTCTGC
CGACAGTGGTCCCAAAGATGGACCCCCACCCACGAGGAGCATCGTGGAAAAAGAAGACGTTCCAA
CCACGTCTTCAAAGCAAGTGGATTGATGTGATATCTCCACTGACGTAAGGGATGACGCACAATCC
CACTATCCTTCGCAAGACCCTTCCTCTATATAAGGAAGTTCATTTCATTTGGAGAGGAGATCTTT
TTATTTTTAATTTTCTTTCAAATACTTCCACCATGGCTCTAGAGGATCCACCGGTCGCCACCATG
GTGAGCAAGGGCGAGGAGCTGTTCACCGGGGTGGTGCCCATCCTGGTCGAGCTGGACGGCGACGT
AAACGGCCACAAGTTCAGCGTGTCCGGCGAGGGCGAGGGCGATGCCACCTACGGCAAGCTGACCC
TGAAGTTCATCTGCACCACCGGCAAGCTGCCCGTGCCCTGGCCCACCCTCGTGACCACCCTGACC
TACGGCGTGCAGTGCTTCAGCCGCTACCCCGACCACATGAAGCAGCACGACTTCTTCAAGTCCGC
CATGCCCGAAGGCTACGTCCAGGAGCGCACCATCTTCTTCAAGGACGACGGCAACTACAAGACCC
GCGCCGAGGTGAAGTTCGAGGGCGACACCCTGGTGAACCGCATCGAGCTGAAGGGCATCGACTTC
AAGGAGGACGGCAACATCCTGGGGCACAAGCTGGAGTACAACTACAACAGCCACAACGTCTATAT
CATGGCCGACAAGCAGAAGAACGGCATCAAGGTGAACTTCAAGATCCGCCACAACATCGAGGACG
GCAGCGTGCAGCTCGCCGACCACTACCAGCAGAACACCCCCATCGGCGACGGCCCCGTGCTGCTG
CCCGACAACCACTACCTGAGCACCCAGTCCGCCCTGAGCAAAGACCCCAACGAGAAGCGCGATCA
CATGGTCCTGCTGGAGTTCGTGACCGCCGCCGGGATCACTCTCGGCATGGACGAGCTGTACAAGT
AAAGCGGCCGCATGCTAGCCTAGGATCCGCGGATATCTGCAGAAGCTTCCCATGGTGACGTCACC
GGTTCTAGATACCTAGGTGAGCTCGAATTTCCCCGATCGTTCAAACATTTGGCAATAAAGTTTCT
TAAGATTGAATCCTGTTGCCGGTCTTGCGATGATTATCATATAATTTCTGTTGAATTACGTTAAG
CATGTAATAATTAACATGTAATGCATGACGTTATTTATGAGATGGGTTTTTATGATTAGAGTCCC
GCAATTATACATTTAATACGCGATAGAAAACAAAATATAGCGCGCAAACTAGGATAAATTATCGC
GCGCGGTGTCATCTATGTTACTAGATCGGGAATTGATCCCCGGGTAGGTGAGACTTTTCAACAAA
GGGTAATATCGGGAAACCTCCTCGGATTCCATTGCCCAGCTATCTGTCACTTCATCAAAAGGACA

GTAGAAAAGGAAGGTGGCACCTACAAATGCCATCATTGCGATAAAGGAAAGGCTATCGTTCAAGA
TGCCCCTGCCGACAGTGGTCCCAAAGATGGACCCCCACCCACGAGGAGCATCGTGGAAAAAGAAG
ACGTTCCAACCACGTCTTCAAAGCAAGTGGATTGATGTGATATCTCCACTGACGTAAGGGATGAC
GCACAATCCCACTATCCTTCGCAAGACCCTTCCTCTATATAAGGAAGTTCATTTCATTTGGAGAG
GACTCCGGTATTTTTACAACAATTACCACAACAAAACAAACAACAAACAACATTACAATTTACTA
TTCTAGTCGAAATGCCCAAGAAGAAGAGGAAGGTGGACAAGAAGTACTCCATTGGGCTCGCTATC
GGCACAAACAGCGTCGGCTGGGCCGTCATTACGGACGAGTACAAGGTGCCGAGCAAAAAATTCAA
AGTTCTGGGCAATACCGATCGCCACAGCATAAAGAAGAACCTCATTGGCGCCCTCCTGTTCGACT
CCGGGGAAACGGCCGAAGCCACGCGGCTCAAAAGAACAGCACGGCGCAGATATACCCGCAGAAAG
AATCGGATCTGCTACCTGCAGGAGATCTTTAGTAATGAGATGGCTAAGGTGGATGACTCTTTCTT
CCATAGGCTGGAGGAGTCCTTTTTGGTGGAGGAGGATAAAAAGCACGAGCGCCACCCAATCTTTG
GCAATATCGTGGACGAGGTGGCGTACCATGAAAAGTACCCAACCATATATCATCTGAGGAAGAAG
CTTGTAGACAGTACTGATAAGGCTGACTTGCGGTTGATCTATCTCGCGCTGGCGCATATGATCAA
ATTTCGGGGACACTTCCTCATCGAGGGGGACCTGAACCCAGACAACAGCGATGTCGACAAACTCT
TTATCCAACTGGTTCAGACTTACAATCAGCTTTTCGAAGAGAACCCGATCAACGCATCCGGAGTT
GACGCCAAAGCAATCCTGAGCGCTAGGCTGTCCAAATCCCGGCGGCTCGAAAACCTCATCGCACA
GCTCCCTGGGGAGAAGAAGAACGGCCTGTTTGGTAATCTTATCGCCCTGTCACTCGGGCTGACCC
CCAACTTTAAATCTAACTTCGACCTGGCCGAAGATGCCAAGCTTCAACTGAGCAAAGACACCTAC
GATGATGATCTCGACAATCTGCTGGCCCAGATCGGCGACCAGTACGCAGACCTTTTTTTGGCGGC
AAAGAACCTGTCAGACGCCATTCTGCTGAGTGATATTCTGCGAGTGAACACGGAGATCACCAAAG
CTCCGCTGAGCGCTAGTATGATCAAGCGCTATGATGAGCACCACCAAGACTTGACTTTGCTGAAG
GCCCTTGTCAGACAGCAACTGCCTGAGAAGTACAAGGAAATTTTCTTCGATCAGTCTAAAAATGG
CTACGCCGGATACATTGACGGCGGAGCAAGCCAGGAGGAATTTTACAAATTTATTAAGCCCATCT
TGGAAAAAATGGACGGCACCGAGGAGCTGCTGGTAAAGCTTAACAGAGAAGATCTGTTGCGCAAA
CAGCGCACTTTCGACAATGGAAGCATCCCCCACCAGATTCACCTGGGCGAACTGCACGCTATCCT
CAGGCGGCAAGAGGATTTCTACCCCTTTTTGAAAGATAACAGGGAAAAGATTGAGAAAATCCTCA
CATTTCGGATACCCTACTATGTAGGCCCCCTCGCCCGGGGAAATTCCAGATTCGCGTGGATGACT
CGCAAATCAGAAGAGACTATCACTCCCTGGAACTTCGAGGAAGTCGTGGATAAGGGGGCCTCTGC
CCAGTCCTTCATCGAAAGGATGACTAACTTTGATAAAAATCTGCCTAACGAAAAGGTGCTTCCTA
AACACTCTCTGCTGTACGAGTACTTCACAGTTTATAACGAGCTCACCAAGGTCAAATACGTCACA
GAAGGGATGAGAAAGCCAGCATTCCTGTCTGGAGAGCAGAAGAAAGCTATCGTGGACCTCCTCTT
CAAGACGAACCGGAAAGTTACCGTGAAACAGCTCAAAGAAGATTATTTCAAAAAGATTGAATGTT
TCGACTCTGTTGAAATCAGCGGAGTGGAGGATCGCTTCAACGCATCCCTGGGAACGTATCACGAT
CTCCTGAAAATCATTAAAGACAAGGACTTCCTGGACAATGAGGAGAACGAGGACATTCTTGAGGA
CATTGTCCTCACCCTTACGTTGTTTGAAGATAGGGAGATGATTGAAGAACGCTTGAAAACTTACG
CTCATCTCTTCGACGACAAAGTCATGAAACAGCTCAAGAGGCGCCGATATACAGGATGGGGGCGG
CTGTCAAGAAAACTGATCAATGGGATCCGAGACAAGCAGAGTGGAAAGACAATCCTGGATTTTCT
TAAGTCCGATGGATTTGCCAACCGGAACTTCATGCAGTTGATCCATGATGACTCTCTCACCTTTA
AGGAGGACATCCAGAAAGCACAAGTTTCTGGCCAGGGGGACAGTCTCCACGAGCACATCGCTAAT

CTTGCAGGTAGCCCAGCTATCAAAAAGGGAATACTGCAGACCGTTAAGGTCGTGGATGAACTCGT
CAAAGTAATGGGAAGGCATAAGCCCGAGAATATCGTTATCGAGATGGCCCGAGAGAACCAAACTA
CCCAGAAGGGACAGAAGAACAGTAGGGAAAGGATGAAGAGGATTGAAGAGGGTATAAAAGAACTG
GGGTCCCAAATCCTTAAGGAACACCCAGTTGAAAACACCCAGCTTCAGAATGAGAAGCTCTACCT
GTACTACCTGCAGAACGGCAGGGACATGTACGTGGATCAGGAACTGGACATCAATCGGCTCTCCG
ACTACGACGTGGATGCCATCGTGCCCCAGTCTTTTCTCAAAGATGATTCTATTGATAATAAAGTG
TTGACAAGATCCGATAAAAATAGAGGGAAGAGTGATAACGTCCCCTCAGAAGAAGTTGTCAAGAA
AATGAAAAATTATTGGCGGCAGCTGCTGAACGCCAAACTGATCACACAACGGAAGTTCGATAATC
TGACTAAGGCTGAACGAGGTGGCCTGTCTGAGTTGGATAAAGCCGGCTTCATCAAAAGGCAGCTT
GTTGAGACACGCCAGATCACCAAGCACGTGGCCCAAATTCTCGATTCACGCATGAACACCAAGTA
CGATGAAAATGACAAACTGATTCGAGAGGTGAAAGTTATTACTCTGAAGTCTAAGCTGGTTTCAG
ATTTCAGAAAGGACTTTCAGTTTTATAAGGTGAGAGAGATCAACAATTACCACCATGCGCATGAT
GCCTACCTGAATGCAGTGGTAGGCACTGCACTTATCAAAAAATATCCCAAGCTTGAATCTGAATT
TGTTTACGGAGACTATAAAGTGTACGATGTTAGGAAAATGATCGCAAAGTCTGAGCAGGAAATAG
GCAAGGCCACCGCTAAGTACTTCTTTTACAGCAATATTATGAATTTTTTCAAGACCGAGATTACA
CTGGCCAATGGAGAGATTCGGAAGCGACCACTTATCGAAACAAACGGAGAAACAGGAGAAATCGT
GTGGGACAAGGGTAGGGATTTCGCGACAGTCCGGAAGGTCCTGTCCATGCCGCAGGTGAACATCG
TTAAAAAGACCGAAGTACAGACCGGAGGCTTCTCCAAGGAAAGTATCCTCCCGAAAAGGAACAGC
GACAAGCTGATCGCACGCAAAAAAGATTGGGACCCCAAGAAATACGGCGGATTCGATTCTCCTAC
AGTCGCTTACAGTGTACTGGTTGTGGCCAAAGTGGAGAAAGGGAAGTCTAAAAAACTCAAAAGCG
TCAAGGAACTGCTGGGCATCACAATCATGGAGCGATCAAGCTTCGAAAAAAACCCCATCGACTTT
CTCGAGGCGAAAGGATATAAAGAGGTCAAAAAAGACCTCATCATTAAGCTTCCCAAGTACTCTCT
CTTTGAGCTTGAAAACGGCCGGAAACGAATGCTCGCTAGTGCGGGCGAGCTGCAGAAAGGTAACG
AGCTGGCACTGCCCTCTAAATACGTTAATTTCTTGTATCTGGCCAGCCACTATGAAAAGCTCAAA
GGATCTCCCGAAGATAATGAGCAGAAGCAGCTGTTCGTGGAACAACACAAACACTACCTTGATGA
GATCATCGAGCAAATAAGCGAATTCTCCAAAAGAGTGATCCTCGCCGACGCTAACCTCGATAAGG
TGCTTTCTGCTTACAATAAGCACAGGGATAAGCCCATCAGGGAGCAGGCAGAAAACATTATCCAC
TTGTTTACTCTGACCAACTTGGGCGCGCCTGCAGCCTTCAAGTACTTCGACACCACCATAGACAG
AAAGCGGTACACCTCTACAAAGGAGGTCCTGGACGCCACACTGATTCATCAGTCAATTACGGGGC
TCTATGAAACAAGAATCGACCTCTCTCAGCTCGGTGGAGACAGCAGGGCTGATTCGGACCCGAAG
AAAAAGAGGAAGGTAGATCCTAAGAAGAAGAGAAAGGTACTGTTGGATCCGGGAACACCTATGGA
CGCTGATTTGGTAGCTTCTTCTACAGTAGTGTGGGAACAAGATGCAGATCCTTTTGCTGGAACAG
CCGACGACTTCCCCGCCTTCAATGAAGAGGAGCTTGCATGGTTGATGGAGCTGTTACCTCAAGGA
GGATCAGGGGGTCTTTTAGATCCAGGTACTCCCATGGATGCAGACCTGGTCGCTTCAAGTACAGT
CGTTTGGGAGCAGGATGCTGATCCATTCGCAGGTACAGCAGATGACTTCCCGGCTTTCAATGAGG
AGGAACTTGCTTGGCTCATGGAATTGCTCCCGCAGGCAAGAGGTGGATCTGGTGGTCTGCTCGAC
CCAGGAACGCCGATGGACGCTGATCTGGTAGCATCTAGTACAGTCTGGGAGCAGGACGCTGACCC
ATTCGCTGGTACGGCTGACGATTTTCCTGCCTTTAATGAGGAAGAGTTAGCCTGGCTGATGGAGT
TATTACCCCAGGGCGGTAGTGGCGGATTATTGGACCCTGGTACGCCGATGGATGCAGACCTTGTG

GCTTCATCAACGGTCGTATGGGAGCAGGATGCCGATCCTTTCGCAGGAACCGCAGACGACTTCCC
AGCCTTTAACGAAGAAGAGCTCGCATGGCTTATGGAACTTTTACCTCAGGCACGTGGAGGAAGTG
GTGGGCTCCTGGATCCAGGCACTCCGATGGACGCAGACTTAGTAGCTAGTTCCACCGTTGTCTGG
GAGCAGGATGCAGATCCTTTTGCTGGTACCGCAGACGACTTCCCTGCTTTCAATGAAGAAGAATT
GGCCTGGCTTATGGAATTACTTCCGCAGGGTGGGTCTGGTGGGCTGCTCGATCCCGGTACGCCGA
TGGATGCTGACTTGGTTGCTAGCAGTACTGTGGTCTGGGAACAGGATGCTGACCCCTTCGCTGGG
ACTGCCGACGACTTCCCAGCCTTCAACGAAGAGGAACTCGCATGGTTGATGGAACTGCTTCCTCA
AGCACGTGGGGGGTCAGGCGGAGGCGGATCTGGCGGTGACGCACTGGACGACTTTGATCTCGACA
TGCTTGGGTCCGATGCTCTCGACGATTTTGACCTCGATATGCTTGGGTCTGACGCATTAGATGAC
TTTGATTTAGACATGTTGGGGTCAGACGCTTTGGACGATTTCGACTTAGATATGTTGGCCAGAGG
TAGTGATGCCCTTGACGACTTCGATTTAGATATGTTGGGCTCTGACGCCCTCGATGACTTCGATC
TGGATATGCTCGGAAGTGACGCCTTGGACGATTTCGACTTGGACATGCTTGGGTCGGACGCCCTC
GACGACTTTGATCTGGATATGTTATGAGCTTGGAATGGATCTTCGATCCCGATCGTTCAAACATT
TGGCAATAAAGTTTCTTAAGATTGAATCCTGTTGCCGGTCTTGCGACGATTATCATATAATTTCT
GTTGAATTACGTTAAGCATGTAATAATTAACATGTAATGCATGACGTTATTTATGAGATGGGTTT
TTATGATTAGAGTCCCGCAATTATACATTTAATACGCGATAGAAAACAAAATATAGCGCGCAAAC
TAGGATAAATTATCGCGCGCGGTGTCATCTATGTTACTAGATCGGGAATTGCCAAGCTAATTCCT
TCGTTGAACAACGGAAACTCGACTTGCCTTCCGCACAATACATCATTTCTTCTTAGCTTTTTTTC
TTCTTCTTCGTTCATACAGTTTTTTTTTGTTTATCAGCTTACATTTTCTTGAACCGTAGCTTTCG
TTTTCTTCTTTTTAACTTTCCATTCGGAGTTTTTGTATCTTGTTTCATAGTTTGTCCCAGGATTA
GAATGATTAGGCATCGAACCTTCAAGAATTTGATTGAATAAAACATCTTCATTCTTAAGATATGA
AGATAATCTTCAAAAGGCCCCTGGGAATCTGAAAGAAGAGAAGCAGGCCCATTTATATGGGAAAG
AACAATAGTATTTCTTATATAGGCCCATTTAAGTTGAAAACAATCTTCAAAAGTCCCACATCGCT
TAGATAAGAAAACGAAGCTGAGTTTATATACAGCTAGAGTCGAAGTAGTGATTGAATCTGTAAGG
AGCTTTCGGGTTTTAGAGCTAGAAATAGCAAGTTAAAATAAGGCTAGTCCGTTATCAACTTGAAA
AAGTGGCACCGAGTCGGTGCTTTTTTTTTGAAGACGAAAGGGCCTCGTGATACGCCTATTTTTAT
AGGTTAATGTCATGATAATAATGGTTTCTTAGACGTCAGGTGGCACTTTTCGGGGAAATGTGCGC
GGAACCCCTATTTGTTTATTTTTCTAAATACATTCAAATATGTATCCGCTCATGAGACAATAACC
CTGATAAATGCTTCAATAATGGGACCGACTCGCGCTTGAGACGAAGCTCCAAGCTTGGCACTGGC
CGTCGTTTTACAACGTCGTGACTGGGAAAACCCTGGCGTTACCCAACTTAATCGCCTTGCAGCAC
ATCCCCCTTTCGCCAGCTGGCGTAATAGCGAAGAGGCCCGCACCGATCGCCCTTCCCAACAGTTG
CGCAGCCTGAATGGCGAATGAGCTTGAGCTTGGATCAGATTGTCGTTTCCCGCCTTCAGTTTAAA
CTATCAGTGTTTGACAGGATATATTGGCGGGTAAACCTAAGAGAAAAGAGCGTTTATTAGAATAA
CGGATATTTAAAAGGGCGTGAAAAGGTTTATCCGTTCGTCCATTTGTATGTGCATGCCAACCACA
GGGTTCCCCTCGGGATCAA3’

Supplementary File2. pPZP_CRISPRa_Pv-lectin sequence.

5’AGTACTTTGATCCAACCCCTCCGCTGCTATAGTGCAGTCGGCTTCTGACGTTCAGTGCAGCCG
TCTTCTGAAAACGACATGTCGCACAAGTCCTAAGTTACGCGACAGGCTGCCGCCCTGCCCTTTTC
CTGGCGTTTTCTTGTCGCGTGTTTTAGTCGCATAAAGTAGAATACTTGCGACTAGAACCGGAGAC
ATTACGCCATGAACAAGAGCGCCGCCGCTGGCCTGCTGGGCTATGCCCGCGTCAGCACCGACGAC
CAGGACTTGACCAACCAACGGGCCGAACTGCACGCGGCCGGCTGCACCAAGCTGTTTTCCGAGAA
GATCACCGGCACCAGGCGCGACCGCCCGGAGCTGGCCAGGATGCTTGACCACCTACGCCCTGGCG
ACGTTGTGACAGTGACCAGGCTAGACCGCCTGGCCCGCAGCACCCGCGACCTACTGGACATTGCC
GAGCGCATCCAGGAGGCCGGCGCGGGCCTGCGTAGCCTGGCAGAGCCGTGGGCCGACACCACCAC
GCCGGCCGGCCGCATGGTGTTGACCGTGTTCGCCGGCATTGCCGAGTTCGAGCGTTCCCTAATCA
TCGACCGCACCCGGAGCGGGCGCGAGGCCGCCAAGGCCCGAGGCGTGAAGTTTGGCCCCCGCCCT
ACCCTCACCCCGGCACAGATCGCGCACGCCCGCGAGCTGATCGACCAGGAAGGCCGCACCGTGAA
AGAGGCGGCTGCACTGCTTGGCGTGCATCGCTCGACCCTGTACCGCGCACTTGAGCGCAGCGAGG
AAGTGACGCCCACCGAGGCCAGGCGGCGCGGTGCCTTCCGTGAGGACGCATTGACCGAGGCCGAC
GCCCTGGCGGCCGCCGAGAATGAACGCCAAGAGGAACAAGCATGAAACCGCACCAGGACGGCCAG
GACGAACCGTTTTTCATTACCGAAGAGATCGAGGCGGAGATGATCGCGGCCGGGTACGTGTTCGA
GCCGCCCGCGCACGTCTCAACCGTGCGGCTGCATGAAATCCTGGCCGGTTTGTCTGATGCCAAGC
TGGCGGCCTGGCCGGCCAGCTTGGCCGCTGAAGAAACCGAGCGCCGCCGTCTAAAAAGGTGATGT
GTATTTGAGTAAAACAGCTTGCGTCATGCGGTCGCTGCGTATATGATGCGATGAGTAAATAAACA
AATACGCAAGGGGAACGCATGAAGGTTATCGCTGTACTTAACCAGAAAGGCGGGTCAGGCAAGAC
GACCATCGCAACCCATCTAGCCCGCGCCCTGCAACTCGCCGGGGCCGATGTTCTGTTAGTCGATT
CCGATCCCCAGGGCAGTGCCCGCGATTGGGCGGCCGTGCGGGAAGATCAACCGCTAACCGTTGTC
GGCATCGACCGCCCGACGATTGACCGCGACGTGAAGGCCATCGGCCGGCGCGACTTCGTAGTGAT
CGACGGAGCGCCCCAGGCGGCGGACTTGGCTGTGTCCGCGATCAAGGCAGCCGACTTCGTGCTGA
TTCCGGTGCAGCCAAGCCCTTACGACATATGGGCCACCGCCGACCTGGTGGAGCTGGTTAAGCAG
CGCATTGAGGTCACGGATGGAAGGCTACAAGCGGCCTTTGTCGTGTCGCGGGCGATCAAAGGCAC
GCGCATCGGCGGTGAGGTTGCCGAGGCGCTGGCCGGGTACGAGCTGCCCATTCTTGAGTCCCGTA
TCACGCAGCGCGTGAGCTACCCAGGCACTGCCGCCGCCGGCACAACCGTTCTTGAATCAGAACCC
GAGGGCGACGCTGCCCGCGAGGTCCAGGCGCTGGCCGCTGAAATTAAATCAAAACTCATTTGAGT
TAATGAGGTAAAGAGAAAATGAGCAAAAGCACAAACACGCTAAGTGCCGGCCGTCCGAGCGCACG
CAGCAGCAAGGCTGCAACGTTGGCCAGCCTGGCAGACACGCCAGCCATGAAGCGGGTCAACTTTC
AGTTGCCGGCGGAGGATCACACCAAGCTGAAGATGTACGCGGTACGCCAAGGCAAGACCATTACC
GAGCTGCTATCTGAATACATCGCGCAGCTACCAGAGTAAATGAGCAAATGAATAAATGAGTAGAT
GAATTTTAGCGGCTAAAGGAGGCGGCATGGAAAATCAAGAACAACCAGGCACCGACGCCGTGGAA
TGCCCCATGTGTGGAGGAACGGGCGGTTGGCCAGGCGTAAGCGGCTGGGTTGTCTGCCGGCCCTG
CAATGGCACTGGAACCCCCAAGCCCGAGGAATCGGCGTGACGGTCGCAAACCATCCGGCCCGGTA
CAAATCGGCGCGGCGCTGGGTGATGACCTGGTGGAGAAGTTGAAGGCCGCGCAGGCCGCCCAGCG
GCAACGCATCGAGGCAGAAGCACGCCCCGGTGAATCGTGGCAAGCGGCCGCTGATCGAATCCGCA
AAGAATCCCGGCAACCGCCGGCAGCCGGTGCGCCGTCGATTAGGAAGCCGCCCAAGGGCGACGAG

CAACCAGATTTTTTCGTTCCGATGCTCTATGACGTGGGCACCCGCGATAGTCGCAGCATCATGGA
CGTGGCCGTTTTCCGTCTGTCGAAGCGTGACCGACGAGCTGGCGAGGTGATCCGCTACGAGCTTC
CAGACGGGCACGTAGAGGTTTCCGCAGGGCCGGCCGGCATGGCCAGTGTGTGGGATTACGACCTG
GTACTGATGGCGGTTTCCCATCTAACCGAATCCATGAACCGATACCGGGAAGGGAAGGGAGACAA
GCCCGGCCGCGTGTTCCGTCCACACGTTGCGGACGTACTCAAGTTCTGCCGGCGAGCCGATGGCG
GAAAGCAGAAAGACGACCTGGTAGAAACCTGCATTCGGTTAAACACCACGCACGTTGCCATGCAG
CGTACGAAGAAGGCCAAGAACGGCCGCCTGGTGACGGTATCCGAGGGTGAAGCCTTGATTAGCCG
CTACAAGATCGTAAAGAGCGAAACCGGGCGGCCGGAGTACATCGAGATCGAGCTAGCTGATTGGA
TGTACCGCGAGATCACAGAAGGCAAGAACCCGGACGTGCTGACGGTTCACCCCGATTACTTTTTG
ATCGATCCCGGCATCGGCCGTTTTCTCTACCGCCTGGCACGCCGCGCCGCAGGCAAGGCAGAAGC
CAGATGGTTGTTCAAGACGATCTACGAACGCAGTGGCAGCGCCGGAGAGTTCAAGAAGTTCTGTT
TCACCGTGCGCAAGCTGATCGGGTCAAATGACCTGCCGGAGTACGATTTGAAGGAGGAGGCGGGG
CAGGCTGGCCCGATCCTAGTCATGCGCTACCGCAACCTGATCGAGGGCGAAGCATCCGCCGGTTC
CTAATGTACGGAGCAGATGCTAGGGCAAATTGCCCTAGCAGGGGAAAAAGGTCGAAAAGGTCTCT
TTCCTGTGGATAGCACGTACATTGGGAACCCAAAGCCGTACATTGGGAACCGGAACCCGTACATT
GGGAACCCAAAGCCGTACATTGGGAACCGGTCACACATGTAAGTGACTGATATAAAAGAGAAAAA
AGGCGATTTTTCCGCCTAAAACTCTTTAAAACTTATTAAAACTCTTAAAACCCGCCTGGCCTGTG
CATAACTGTCTGGCCAGCGCACAGCCGAAGAGCTGCAAAAAGCGCCTACCCTTCGGTCGCTGCGC
TCCCTACGCCCCGCCGCTTCGCGTCGGCCTATCGCGGCCGCTGGCCGCTCAAAAATGGCTGGCCT
ACGGCCAGGCAATCTACCAGGGCGCGGACAAGCCGCGCCGTCGCCACTCGACCGCCGGCGCCCAC
ATCAAGGCACCCTGCCTCGCGCGTTTCGGTGATGACGGTGAAAACCTCTGACACATGCAGCTCCC
GGAGACGGTCACAGCTTGTCTGTAAGCGGATGCCGGGAGCAGACAAGCCCGTCAGGGCGCGTCAG
CGGGTGTTGGCGGGTGTCGGGGCGCAGCCATGACCCAGTCACGTAGCGATAGCGGAGTGTATACT
GGCTTAACTATGCGGCATCAGAGCAGATTGTACTGAGAGTGCACCATATGCGGTGTGAAATACCG
CACAGATGCGTAAGGAGAAAATACCGCATCAGGCGCTCTTCCGCTTCCTCGCTCACTGACTCGCT
GCGCTCGGTCGTTCGGCTGCGGCGAGCGGTATCAGCTCACTCAAAGGCGGTAATACGGTTATCCA
CAGAATCAGGGGATAACGCAGGAAAGAACATGTGAGCAAAAGGCCAGCAAAAGGCCAGGAACCGT
AAAAAGGCCGCGTTGCTGGCGTTTTTCCATAGGCTCCGCCCCCCTGACGAGCATCACAAAAATCG
ACGCTCAAGTCAGAGGTGGCGAAACCCGACAGGACTATAAAGATACCAGGCGTTTCCCCCTGGAA
GCTCCCTCGTGCGCTCTCCTGTTCCGACCCTGCCGCTTACCGGATACCTGTCCGCCTTTCTCCCT
TCGGGAAGCGTGGCGCTTTCTCATAGCTCACGCTGTAGGTATCTCAGTTCGGTGTAGGTCGTTCG
CTCCAAGCTGGGCTGTGTGCACGAACCCCCCGTTCAGCCCGACCGCTGCGCCTTATCCGGTAACT
ATCGTCTTGAGTCCAACCCGGTAAGACACGACTTATCGCCACTGGCAGCAGCCACTGGTAACAGG
ATTAGCAGAGCGAGGTATGTAGGCGGTGCTACAGAGTTCTTGAAGTGGTGGCCTAACTACGGCTA
CACTAGAAGGACAGTATTTGGTATCTGCGCTCTGCTGAAGCCAGTTACCTTCGGAAAAAGAGTTG
GTAGCTCTTGATCCGGCAAACAAACCACCGCTGGTAGCGGTGGTTTTTTTGTTTGCAAGCAGCAG
ATTACGCGCAGAAAAAAAGGATCTCAAGAAGATCCTTTGATCTTTTCTACGGGGTCTGACGCTCA
GTGGAACGAAAACTCACGTTAAGGGATTTTGGTCATGCAGGATCATGAATTAATTCTTAGAAAAA
CTCATCGAGCATCAAATGAAACTGCAATTTATTCATATCAGGATTATCAATACCATATTTTTGAA

AAAGCCGTTTCTGTAATGAAGGAGAAAACTCACCGAGGCAGTTCCATAGGATGGCAAGATCCTGG
TATCGGTCTGCGATTCCGACTCGTCCAACATCAATACAACCTATTAATTTCCCCTCGTCAAAAAT
AAGGTTATCAAGTGAGAAATCACCATGAGTGACGACTGAATCCGGTGAGAATGGCAAAAGTTTAT
GCATTTCTTTCCAGACTTGTTCAACAGGCCAGCCATTACGCTCGTCATCAAAATCACTCGCATCA
ACCAAACCGTTATTCATTCGTGATTGCGCCTGAGCGAGACGAAATACGCGATCGCTGTTAAAAGG
ACAATTACAAACAGGAATCGAATGCAACCGGCGCAGGAACACTGCCAGCGCATCAACAATATTTT
CACCTGAATCAGGATATTCTTCTAATACCTGGAATGCTGTTTTCCCGGGGATCGCAGTGGTGAGT
AACCATGCATCATCAGGAGTACGGATAAAATGCTTGATGGTCGGAAGAGGCATAAATTCCGTCAG
CCAGTTTAGTCTGACCATCTCATCTGTAACATCATTGGCAACGCTACCTTTGCCATGTTTCAGAA
ACAACTCTGGCGCATCGGGCTTCCCATACAATCGATAGATTGTCGCACCTGATTGCCCGACATTA
TCGCGAGCCCATTTATACCCATATAAATCAGCATCCATGTTGGAATTTAATCGCGGCCTAGAGCA
AGACGTTTCCCGTTGAATATGGCTCATAACACCCCTTGTATTACTGTTTATGTAAGCAGACAGTT
TTATTGTTCATGATCTGGATCACAGGCAGCAACGCTCTGTCATCGTTACAATCAACATGCTACCC
TCCGCGAGATCATCCGTGTTTCAAACCCGGCAGCTTAGTTGCCGTTCTTCCGAATAGCATCGGTA
ACATGAGCAAAGTCTGCCGCCTTACAACGGCTCTCCCGCTGACGCCGTCCCGGACTGATGGGCTG
CCTGTATCGAGTGGTGATTTTGTGCCGAGCTGCCGGTCGGGGAGCTGTTGGCTGGCTGGTGGCAG
GATATATTGTGGTGTAAACAAATTGACGCTTAGACAACTTAATAACACATTGCGGACGTTTTTAA
TGTACTGAATTAACGCCGAATTGCTCTAGCCAATACGCAAACCGCCTCTCCCCGCGCGTTGGCCG
ATTCATTAATGCAGCTGGCACGACAGGTTTCCCGACTGGAAAGCGGGCAGTGAGCGCAACGCAAT
TAATGTGAGTTAGCTCACTCATTAGGCACCCCAGGCTTTACACTTTATGCTTCCGGCTCGTATGT
TGTGTGGAATTGTGAGCGGATAACAATTTCACACAGGAAACAGCTATGACATGATTACGAATTCT
TAATTAAGCTACCCGGGGATCAATTCCCGATCTAGTAACATAGATGACACCGCGCGCGATAATTT
ATCCTAGTTTGCGCGCTATATTTTGTTTTCTATCGCGTATTAAATGTATAATTGCGGGACTCTAA
TCATAAAAACCCATCTCATAAATAACGTCATGCATTACATGTTAATTATTACATGCTTAACGTAA
TTCAACAGAAATTATATGATAATCATCGCAAGACCGGCAACAGGATTCAATCTTAAGAAACTTTA
TTGCCAAATGTTTGAACGATCGGGGAAATTCGAGCTTCGTGGATCCCGGTCGGCATCTACTTCAA
ATCTCGGTGACGGGCAGGACCGGACGGGGCGGTACCGGCAGGCTGAAGTCCAGCTGCCAGAAACC
CACGTCATGCCAGTTCCCGTGCTTGAAGCCGGCCGCCCGCAGCATGCCGCGGGGGGCATATCCGA
GCGCCTCGTGCATGCGCACGCTCGGGTCGTTGGGCAGCCCGATGACAGCGACCACGCTCTTGAAG
CCCTGTGCCTCCAGGGACTTCAGCAGGTGGGTGTAGAGCGTGGAGCCCAGTCCCGTCCGCTGGTG
GCGGGGGGAGACGTACACGGTCGACTCGGCCGTCCAGTCGTAGGCGTTGCGTGCCTTCCAGGGGC
CCGCGTAGGCGATGCCGGCGACCTCGCCGTCCACCTCGGCGACGAGCCAGGGATAGCGCTCCCGC
AGACGGACGAGGTCGTCCGTCCACTCCTGCGGTTCCTGCGGCTCGGTACGGAAGTTGACCGTGCT
TGTCTCGATGTAGTGGTTGACGATGGTGCAGACCGCCGGCATGTCCGCCTCGGTGGCACGGCGGA
TGTCGGCCGGGCGTCGTTCTGGGCTCATATCTTATTGCCCCCCTAGAGTCGAGATCCTTCGCCTG
GAGGAGAGAAATCAGTGGCGCTGCGGCTTTTAGGGTTTCTTTGTTGATGGAATGAGAGTGTAAGC
TCTGCCAGTGCCACTTTATTAGGGTTTTACAAGCCCTTTTCTTCGTAATTGGGCCTGACATTTTG
TGCCACTTGGGCCTTTAGAGATGAAAATGTATATTGGGCTTAAGTTGACTTGAAGGATAAATTAG
TTTAGGATATTACGTTTTTTATGAGAATTGGTGTGTCGGATACATGTATATGATGCATTCAAATA

TATGTATTCTAGATACATTTAAGTTTAGATACAATCTAAAATGTGTCTTTAATTACAGGACTGTA
ACTAAAATACTTAATGTAAGAAGAATATTACTCCTTTAATAGCTTTTGAGTATATCTAGTCTAAC
ATCTTTTAAAAAAGTCTAATTTCTTTCATTTATTTTTCGAGCAATAGCAAAGTGCATAATTATTT
TTTTCTTCTAGAAATTCAGATTTGTTTCTCTAAATTTTGAGATTCTTTTCTCAATTTTGTATGTC
TAGAGAACAATGTGTATTTTTCACTCTAGTTGGTTGTTGCTTTGTTGAATGTTCTGATAAAAGTA
TATTGTTATTTCTGAAGTAGATATAAACCTTCATTTGGAAATTATACATAAATCAAAATCGTTAA
TTATCTAGATCAAGATATATGCCCTTTTCCTAATGTATTTGATACATGCACCTAATTTCACTAGA
TGTATCTTTTCTATTTTTTAAATTATGAATAGTTAATTTTTTCCATATGTGTATTTGATACATAC
TTCATGACTTTAAAAAATTAATTATATACCAGATATATGTATTTAAAATTTGTTATGTATTTAAA
GTATGTATATGATTATTCGATATTAATCTCTTCGATGAAATTTAAATCGATAACTATGTGCTTTG
GATCTGCCCACTATCGAGCTCAAGCTTGCATGCCTGCAGGTCCGATCTGAGACTTTTCAACAAAG
GGTAATATCGGGAAACCTCCTCGGATTCCATTGCCCAGCTATCTGTCACTTCATCAAAAGGACAG
TAGAAAAGGAAGGTGGCACCTACAAATGCCATCATTGCGATAAAGGAAAGGCTATCGTTCAAGAT
GCCTCTGCCGACAGTGGTCCCAAAGATGGACCCCCACCCACGAGGAGCATCGTGGAAAAAGAAGA
CGTTCCAACCACGTCTTCAAAGCAAGTGGATTGATGTGATGGTCCGATTGAGACTTTTCAACAAA
GGGTAATATCGGGAAACCTCCTCGGATTCCATTGCCCAGCTATCTGTCACTTCATCAAAAGGACA
GTAGAAAAGGAAGGTGGCACCTACAAATGCCATCATTGCGATAAAGGAAAGGCTATCGTTCAAGA
TGCCTCTGCCGACAGTGGTCCCAAAGATGGACCCCCACCCACGAGGAGCATCGTGGAAAAAGAAG
ACGTTCCAACCACGTCTTCAAAGCAAGTGGATTGATGTGATATCTCCACTGACGTAAGGGATGAC
GCACAATCCCACTATCCTTCGCAAGACCCTTCCTCTATATAAGGAAGTTCATTTCATTTGGAGAG
GAGATCTTTTTATTTTTAATTTTCTTTCAAATACTTCCACCATGGCTCTAGAGGATCCACCGGTC
GCCACCATGGTGAGCAAGGGCGAGGAGCTGTTCACCGGGGTGGTGCCCATCCTGGTCGAGCTGGA
CGGCGACGTAAACGGCCACAAGTTCAGCGTGTCCGGCGAGGGCGAGGGCGATGCCACCTACGGCA
AGCTGACCCTGAAGTTCATCTGCACCACCGGCAAGCTGCCCGTGCCCTGGCCCACCCTCGTGACC
ACCCTGACCTACGGCGTGCAGTGCTTCAGCCGCTACCCCGACCACATGAAGCAGCACGACTTCTT
CAAGTCCGCCATGCCCGAAGGCTACGTCCAGGAGCGCACCATCTTCTTCAAGGACGACGGCAACT
ACAAGACCCGCGCCGAGGTGAAGTTCGAGGGCGACACCCTGGTGAACCGCATCGAGCTGAAGGGC
ATCGACTTCAAGGAGGACGGCAACATCCTGGGGCACAAGCTGGAGTACAACTACAACAGCCACAA
CGTCTATATCATGGCCGACAAGCAGAAGAACGGCATCAAGGTGAACTTCAAGATCCGCCACAACA
TCGAGGACGGCAGCGTGCAGCTCGCCGACCACTACCAGCAGAACACCCCCATCGGCGACGGCCCC
GTGCTGCTGCCCGACAACCACTACCTGAGCACCCAGTCCGCCCTGAGCAAAGACCCCAACGAGAA
GCGCGATCACATGGTCCTGCTGGAGTTCGTGACCGCCGCCGGGATCACTCTCGGCATGGACGAGC
TGTACAAGTAAAGCGGCCGCATGCTAGCCTAGGATCCGCGGATATCTGCAGAAGCTTCCCATGGT
GACGTCACCGGTTCTAGATACCTAGGTGAGCTCGAATTTCCCCGATCGTTCAAACATTTGGCAAT
AAAGTTTCTTAAGATTGAATCCTGTTGCCGGTCTTGCGATGATTATCATATAATTTCTGTTGAAT
TACGTTAAGCATGTAATAATTAACATGTAATGCATGACGTTATTTATGAGATGGGTTTTTATGAT
TAGAGTCCCGCAATTATACATTTAATACGCGATAGAAAACAAAATATAGCGCGCAAACTAGGATA
AATTATCGCGCGCGGTGTCATCTATGTTACTAGATCGGGAATTGATCCCCGGGTAGGTGAGACTT
TTCAACAAAGGGTAATATCGGGAAACCTCCTCGGATTCCATTGCCCAGCTATCTGTCACTTCATC

AAAAGGACAGTAGAAAAGGAAGGTGGCACCTACAAATGCCATCATTGCGATAAAGGAAAGGCTAT
CGTTCAAGATGCCCCTGCCGACAGTGGTCCCAAAGATGGACCCCCACCCACGAGGAGCATCGTGG
AAAAAGAAGACGTTCCAACCACGTCTTCAAAGCAAGTGGATTGATGTGATATCTCCACTGACGTA
AGGGATGACGCACAATCCCACTATCCTTCGCAAGACCCTTCCTCTATATAAGGAAGTTCATTTCA
TTTGGAGAGGACTCCGGTATTTTTACAACAATTACCACAACAAAACAAACAACAAACAACATTAC
AATTTACTATTCTAGTCGAAATGCCCAAGAAGAAGAGGAAGGTGGACAAGAAGTACTCCATTGGG
CTCGCTATCGGCACAAACAGCGTCGGCTGGGCCGTCATTACGGACGAGTACAAGGTGCCGAGCAA
AAAATTCAAAGTTCTGGGCAATACCGATCGCCACAGCATAAAGAAGAACCTCATTGGCGCCCTCC
TGTTCGACTCCGGGGAAACGGCCGAAGCCACGCGGCTCAAAAGAACAGCACGGCGCAGATATACC
CGCAGAAAGAATCGGATCTGCTACCTGCAGGAGATCTTTAGTAATGAGATGGCTAAGGTGGATGA
CTCTTTCTTCCATAGGCTGGAGGAGTCCTTTTTGGTGGAGGAGGATAAAAAGCACGAGCGCCACC
CAATCTTTGGCAATATCGTGGACGAGGTGGCGTACCATGAAAAGTACCCAACCATATATCATCTG
AGGAAGAAGCTTGTAGACAGTACTGATAAGGCTGACTTGCGGTTGATCTATCTCGCGCTGGCGCA
TATGATCAAATTTCGGGGACACTTCCTCATCGAGGGGGACCTGAACCCAGACAACAGCGATGTCG
ACAAACTCTTTATCCAACTGGTTCAGACTTACAATCAGCTTTTCGAAGAGAACCCGATCAACGCA
TCCGGAGTTGACGCCAAAGCAATCCTGAGCGCTAGGCTGTCCAAATCCCGGCGGCTCGAAAACCT
CATCGCACAGCTCCCTGGGGAGAAGAAGAACGGCCTGTTTGGTAATCTTATCGCCCTGTCACTCG
GGCTGACCCCCAACTTTAAATCTAACTTCGACCTGGCCGAAGATGCCAAGCTTCAACTGAGCAAA
GACACCTACGATGATGATCTCGACAATCTGCTGGCCCAGATCGGCGACCAGTACGCAGACCTTTT
TTTGGCGGCAAAGAACCTGTCAGACGCCATTCTGCTGAGTGATATTCTGCGAGTGAACACGGAGA
TCACCAAAGCTCCGCTGAGCGCTAGTATGATCAAGCGCTATGATGAGCACCACCAAGACTTGACT
TTGCTGAAGGCCCTTGTCAGACAGCAACTGCCTGAGAAGTACAAGGAAATTTTCTTCGATCAGTC
TAAAAATGGCTACGCCGGATACATTGACGGCGGAGCAAGCCAGGAGGAATTTTACAAATTTATTA
AGCCCATCTTGGAAAAAATGGACGGCACCGAGGAGCTGCTGGTAAAGCTTAACAGAGAAGATCTG
TTGCGCAAACAGCGCACTTTCGACAATGGAAGCATCCCCCACCAGATTCACCTGGGCGAACTGCA
CGCTATCCTCAGGCGGCAAGAGGATTTCTACCCCTTTTTGAAAGATAACAGGGAAAAGATTGAGA
AAATCCTCACATTTCGGATACCCTACTATGTAGGCCCCCTCGCCCGGGGAAATTCCAGATTCGCG
TGGATGACTCGCAAATCAGAAGAGACTATCACTCCCTGGAACTTCGAGGAAGTCGTGGATAAGGG
GGCCTCTGCCCAGTCCTTCATCGAAAGGATGACTAACTTTGATAAAAATCTGCCTAACGAAAAGG
TGCTTCCTAAACACTCTCTGCTGTACGAGTACTTCACAGTTTATAACGAGCTCACCAAGGTCAAA
TACGTCACAGAAGGGATGAGAAAGCCAGCATTCCTGTCTGGAGAGCAGAAGAAAGCTATCGTGGA
CCTCCTCTTCAAGACGAACCGGAAAGTTACCGTGAAACAGCTCAAAGAAGATTATTTCAAAAAGA
TTGAATGTTTCGACTCTGTTGAAATCAGCGGAGTGGAGGATCGCTTCAACGCATCCCTGGGAACG
TATCACGATCTCCTGAAAATCATTAAAGACAAGGACTTCCTGGACAATGAGGAGAACGAGGACAT
TCTTGAGGACATTGTCCTCACCCTTACGTTGTTTGAAGATAGGGAGATGATTGAAGAACGCTTGA
AAACTTACGCTCATCTCTTCGACGACAAAGTCATGAAACAGCTCAAGAGGCGCCGATATACAGGA
TGGGGGCGGCTGTCAAGAAAACTGATCAATGGGATCCGAGACAAGCAGAGTGGAAAGACAATCCT
GGATTTTCTTAAGTCCGATGGATTTGCCAACCGGAACTTCATGCAGTTGATCCATGATGACTCTC
TCACCTTTAAGGAGGACATCCAGAAAGCACAAGTTTCTGGCCAGGGGGACAGTCTCCACGAGCAC

ATCGCTAATCTTGCAGGTAGCCCAGCTATCAAAAAGGGAATACTGCAGACCGTTAAGGTCGTGGA
TGAACTCGTCAAAGTAATGGGAAGGCATAAGCCCGAGAATATCGTTATCGAGATGGCCCGAGAGA
ACCAAACTACCCAGAAGGGACAGAAGAACAGTAGGGAAAGGATGAAGAGGATTGAAGAGGGTATA
AAAGAACTGGGGTCCCAAATCCTTAAGGAACACCCAGTTGAAAACACCCAGCTTCAGAATGAGAA
GCTCTACCTGTACTACCTGCAGAACGGCAGGGACATGTACGTGGATCAGGAACTGGACATCAATC
GGCTCTCCGACTACGACGTGGATGCCATCGTGCCCCAGTCTTTTCTCAAAGATGATTCTATTGAT
AATAAAGTGTTGACAAGATCCGATAAAAATAGAGGGAAGAGTGATAACGTCCCCTCAGAAGAAGT
TGTCAAGAAAATGAAAAATTATTGGCGGCAGCTGCTGAACGCCAAACTGATCACACAACGGAAGT
TCGATAATCTGACTAAGGCTGAACGAGGTGGCCTGTCTGAGTTGGATAAAGCCGGCTTCATCAAA
AGGCAGCTTGTTGAGACACGCCAGATCACCAAGCACGTGGCCCAAATTCTCGATTCACGCATGAA
CACCAAGTACGATGAAAATGACAAACTGATTCGAGAGGTGAAAGTTATTACTCTGAAGTCTAAGC
TGGTTTCAGATTTCAGAAAGGACTTTCAGTTTTATAAGGTGAGAGAGATCAACAATTACCACCAT
GCGCATGATGCCTACCTGAATGCAGTGGTAGGCACTGCACTTATCAAAAAATATCCCAAGCTTGA
ATCTGAATTTGTTTACGGAGACTATAAAGTGTACGATGTTAGGAAAATGATCGCAAAGTCTGAGC
AGGAAATAGGCAAGGCCACCGCTAAGTACTTCTTTTACAGCAATATTATGAATTTTTTCAAGACC
GAGATTACACTGGCCAATGGAGAGATTCGGAAGCGACCACTTATCGAAACAAACGGAGAAACAGG
AGAAATCGTGTGGGACAAGGGTAGGGATTTCGCGACAGTCCGGAAGGTCCTGTCCATGCCGCAGG
TGAACATCGTTAAAAAGACCGAAGTACAGACCGGAGGCTTCTCCAAGGAAAGTATCCTCCCGAAA
AGGAACAGCGACAAGCTGATCGCACGCAAAAAAGATTGGGACCCCAAGAAATACGGCGGATTCGA
TTCTCCTACAGTCGCTTACAGTGTACTGGTTGTGGCCAAAGTGGAGAAAGGGAAGTCTAAAAAAC
TCAAAAGCGTCAAGGAACTGCTGGGCATCACAATCATGGAGCGATCAAGCTTCGAAAAAAACCCC
ATCGACTTTCTCGAGGCGAAAGGATATAAAGAGGTCAAAAAAGACCTCATCATTAAGCTTCCCAA
GTACTCTCTCTTTGAGCTTGAAAACGGCCGGAAACGAATGCTCGCTAGTGCGGGCGAGCTGCAGA
AAGGTAACGAGCTGGCACTGCCCTCTAAATACGTTAATTTCTTGTATCTGGCCAGCCACTATGAA
AAGCTCAAAGGATCTCCCGAAGATAATGAGCAGAAGCAGCTGTTCGTGGAACAACACAAACACTA
CCTTGATGAGATCATCGAGCAAATAAGCGAATTCTCCAAAAGAGTGATCCTCGCCGACGCTAACC
TCGATAAGGTGCTTTCTGCTTACAATAAGCACAGGGATAAGCCCATCAGGGAGCAGGCAGAAAAC
ATTATCCACTTGTTTACTCTGACCAACTTGGGCGCGCCTGCAGCCTTCAAGTACTTCGACACCAC
CATAGACAGAAAGCGGTACACCTCTACAAAGGAGGTCCTGGACGCCACACTGATTCATCAGTCAA
TTACGGGGCTCTATGAAACAAGAATCGACCTCTCTCAGCTCGGTGGAGACAGCAGGGCTGATTCG
GACCCGAAGAAAAAGAGGAAGGTAGATCCTAAGAAGAAGAGAAAGGTACTGTTGGATCCGGGAAC
ACCTATGGACGCTGATTTGGTAGCTTCTTCTACAGTAGTGTGGGAACAAGATGCAGATCCTTTTG
CTGGAACAGCCGACGACTTCCCCGCCTTCAATGAAGAGGAGCTTGCATGGTTGATGGAGCTGTTA
CCTCAAGGAGGATCAGGGGGTCTTTTAGATCCAGGTACTCCCATGGATGCAGACCTGGTCGCTTC
AAGTACAGTCGTTTGGGAGCAGGATGCTGATCCATTCGCAGGTACAGCAGATGACTTCCCGGCTT
TCAATGAGGAGGAACTTGCTTGGCTCATGGAATTGCTCCCGCAGGCAAGAGGTGGATCTGGTGGT
CTGCTCGACCCAGGAACGCCGATGGACGCTGATCTGGTAGCATCTAGTACAGTCTGGGAGCAGGA
CGCTGACCCATTCGCTGGTACGGCTGACGATTTTCCTGCCTTTAATGAGGAAGAGTTAGCCTGGC
TGATGGAGTTATTACCCCAGGGCGGTAGTGGCGGATTATTGGACCCTGGTACGCCGATGGATGCA

GACCTTGTGGCTTCATCAACGGTCGTATGGGAGCAGGATGCCGATCCTTTCGCAGGAACCGCAGA
CGACTTCCCAGCCTTTAACGAAGAAGAGCTCGCATGGCTTATGGAACTTTTACCTCAGGCACGTG
GAGGAAGTGGTGGGCTCCTGGATCCAGGCACTCCGATGGACGCAGACTTAGTAGCTAGTTCCACC
GTTGTCTGGGAGCAGGATGCAGATCCTTTTGCTGGTACCGCAGACGACTTCCCTGCTTTCAATGA
AGAAGAATTGGCCTGGCTTATGGAATTACTTCCGCAGGGTGGGTCTGGTGGGCTGCTCGATCCCG
GTACGCCGATGGATGCTGACTTGGTTGCTAGCAGTACTGTGGTCTGGGAACAGGATGCTGACCCC
TTCGCTGGGACTGCCGACGACTTCCCAGCCTTCAACGAAGAGGAACTCGCATGGTTGATGGAACT
GCTTCCTCAAGCACGTGGGGGGTCAGGCGGAGGCGGATCTGGCGGTGACGCACTGGACGACTTTG
ATCTCGACATGCTTGGGTCCGATGCTCTCGACGATTTTGACCTCGATATGCTTGGGTCTGACGCA
TTAGATGACTTTGATTTAGACATGTTGGGGTCAGACGCTTTGGACGATTTCGACTTAGATATGTT
GGCCAGAGGTAGTGATGCCCTTGACGACTTCGATTTAGATATGTTGGGCTCTGACGCCCTCGATG
ACTTCGATCTGGATATGCTCGGAAGTGACGCCTTGGACGATTTCGACTTGGACATGCTTGGGTCG
GACGCCCTCGACGACTTTGATCTGGATATGTTATGAGCTTGGAATGGATCTTCGATCCCGATCGT
TCAAACATTTGGCAATAAAGTTTCTTAAGATTGAATCCTGTTGCCGGTCTTGCGACGATTATCAT
ATAATTTCTGTTGAATTACGTTAAGCATGTAATAATTAACATGTAATGCATGACGTTATTTATGA
GATGGGTTTTTATGATTAGAGTCCCGCAATTATACATTTAATACGCGATAGAAAACAAAATATAG
CGCGCAAACTAGGATAAATTATCGCGCGCGGTGTCATCTATGTTACTAGATCGGGAATTGCCAAG
CTAATTCCTTCGTTGAACAACGGAAACTCGACTTGCCTTCCGCACAATACATCATTTCTTCTTAG
CTTTTTTTCTTCTTCTTCGTTCATACAGTTTTTTTTTGTTTATCAGCTTACATTTTCTTGAACCG
TAGCTTTCGTTTTCTTCTTTTTAACTTTCCATTCGGAGTTTTTGTATCTTGTTTCATAGTTTGTC
CCAGGATTAGAATGATTAGGCATCGAACCTTCAAGAATTTGATTGAATAAAACATCTTCATTCTT
AAGATATGAAGATAATCTTCAAAAGGCCCCTGGGAATCTGAAAGAAGAGAAGCAGGCCCATTTAT
ATGGGAAAGAACAATAGTATTTCTTATATAGGCCCATTTAAGTTGAAAACAATCTTCAAAAGTCC
CACATCGCTTAGATAAGAAAACGAAGCTGAGTTTATATACAGCTAGAGTCGAAGTAGTGATTGAA
GCGGTTGGCACGTAGCATGTTTTAGAGCTAGAAATAGCAAGTTAAAATAAGGCTAGTCCGTTATC
AACTTGAAAAAGTGGCACCGAGTCGGTGCTTTTTTTTTGAAGACGAAAGGGCCTCGTGATACGCC
TATTTTTATAGGTTAATGTCATGATAATAATGGTTTCTTAGACGTCAGGTGGCACTTTTCGGGGA
AATGTGCGCGGAACCCCTATTTGTTTATTTTTCTAAATACATTCAAATATGTATCCGCTCATGAG
ACAATAACCCTGATAAATGCTTCAATAATGGGACCGACTCGCGCTTGAGACGAAGCTCCAAGCTT
GGCACTGGCCGTCGTTTTACAACGTCGTGACTGGGAAAACCCTGGCGTTACCCAACTTAATCGCC
TTGCAGCACATCCCCCTTTCGCCAGCTGGCGTAATAGCGAAGAGGCCCGCACCGATCGCCCTTCC
CAACAGTTGCGCAGCCTGAATGGCGAATGAGCTTGAGCTTGGATCAGATTGTCGTTTCCCGCCTT
CAGTTTAAACTATCAGTGTTTGACAGGATATATTGGCGGGTAAACCTAAGAGAAAAGAGCGTTTA
TTAGAATAACGGATATTTAAAAGGGCGTGAAAAGGTTTATCCGTTCGTCCATTTGTATGTGCATG
CCAACCACAGGGTTCCCCTCGGGATCAA3’

Supplementary File 3.pPZP_CRISPRa_Pv-thionin sequence.

5′AGTACTTTGATCCAACCCCTCCGCTGCTATAGTGCAGTCGGCTTCTGACGTTCAGTGCAGCCG
TCTTCTGAAAACGACATGTCGCACAAGTCCTAAGTTACGCGACAGGCTGCCGCCCTGCCCTTTTC
CTGGCGTTTTCTTGTCGCGTGTTTTAGTCGCATAAAGTAGAATACTTGCGACTAGAACCGGAGAC
ATTACGCCATGAACAAGAGCGCCGCCGCTGGCCTGCTGGGCTATGCCCGCGTCAGCACCGACGAC
CAGGACTTGACCAACCAACGGGCCGAACTGCACGCGGCCGGCTGCACCAAGCTGTTTTCCGAGAA
GATCACCGGCACCAGGCGCGACCGCCCGGAGCTGGCCAGGATGCTTGACCACCTACGCCCTGGCG
ACGTTGTGACAGTGACCAGGCTAGACCGCCTGGCCCGCAGCACCCGCGACCTACTGGACATTGCC
GAGCGCATCCAGGAGGCCGGCGCGGGCCTGCGTAGCCTGGCAGAGCCGTGGGCCGACACCACCAC
GCCGGCCGGCCGCATGGTGTTGACCGTGTTCGCCGGCATTGCCGAGTTCGAGCGTTCCCTAATCA
TCGACCGCACCCGGAGCGGGCGCGAGGCCGCCAAGGCCCGAGGCGTGAAGTTTGGCCCCCGCCCT
ACCCTCACCCCGGCACAGATCGCGCACGCCCGCGAGCTGATCGACCAGGAAGGCCGCACCGTGAA
AGAGGCGGCTGCACTGCTTGGCGTGCATCGCTCGACCCTGTACCGCGCACTTGAGCGCAGCGAGG
AAGTGACGCCCACCGAGGCCAGGCGGCGCGGTGCCTTCCGTGAGGACGCATTGACCGAGGCCGAC
GCCCTGGCGGCCGCCGAGAATGAACGCCAAGAGGAACAAGCATGAAACCGCACCAGGACGGCCAG
GACGAACCGTTTTTCATTACCGAAGAGATCGAGGCGGAGATGATCGCGGCCGGGTACGTGTTCGA
GCCGCCCGCGCACGTCTCAACCGTGCGGCTGCATGAAATCCTGGCCGGTTTGTCTGATGCCAAGC
TGGCGGCCTGGCCGGCCAGCTTGGCCGCTGAAGAAACCGAGCGCCGCCGTCTAAAAAGGTGATGT
GTATTTGAGTAAAACAGCTTGCGTCATGCGGTCGCTGCGTATATGATGCGATGAGTAAATAAACA
AATACGCAAGGGGAACGCATGAAGGTTATCGCTGTACTTAACCAGAAAGGCGGGTCAGGCAAGAC
GACCATCGCAACCCATCTAGCCCGCGCCCTGCAACTCGCCGGGGCCGATGTTCTGTTAGTCGATT
CCGATCCCCAGGGCAGTGCCCGCGATTGGGCGGCCGTGCGGGAAGATCAACCGCTAACCGTTGTC
GGCATCGACCGCCCGACGATTGACCGCGACGTGAAGGCCATCGGCCGGCGCGACTTCGTAGTGAT
CGACGGAGCGCCCCAGGCGGCGGACTTGGCTGTGTCCGCGATCAAGGCAGCCGACTTCGTGCTGA
TTCCGGTGCAGCCAAGCCCTTACGACATATGGGCCACCGCCGACCTGGTGGAGCTGGTTAAGCAG
CGCATTGAGGTCACGGATGGAAGGCTACAAGCGGCCTTTGTCGTGTCGCGGGCGATCAAAGGCAC
GCGCATCGGCGGTGAGGTTGCCGAGGCGCTGGCCGGGTACGAGCTGCCCATTCTTGAGTCCCGTA
TCACGCAGCGCGTGAGCTACCCAGGCACTGCCGCCGCCGGCACAACCGTTCTTGAATCAGAACCC
GAGGGCGACGCTGCCCGCGAGGTCCAGGCGCTGGCCGCTGAAATTAAATCAAAACTCATTTGAGT
TAATGAGGTAAAGAGAAAATGAGCAAAAGCACAAACACGCTAAGTGCCGGCCGTCCGAGCGCACG
CAGCAGCAAGGCTGCAACGTTGGCCAGCCTGGCAGACACGCCAGCCATGAAGCGGGTCAACTTTC
AGTTGCCGGCGGAGGATCACACCAAGCTGAAGATGTACGCGGTACGCCAAGGCAAGACCATTACC
GAGCTGCTATCTGAATACATCGCGCAGCTACCAGAGTAAATGAGCAAATGAATAAATGAGTAGAT
GAATTTTAGCGGCTAAAGGAGGCGGCATGGAAAATCAAGAACAACCAGGCACCGACGCCGTGGAA
TGCCCCATGTGTGGAGGAACGGGCGGTTGGCCAGGCGTAAGCGGCTGGGTTGTCTGCCGGCCCTG
CAATGGCACTGGAACCCCCAAGCCCGAGGAATCGGCGTGACGGTCGCAAACCATCCGGCCCGGTA
CAAATCGGCGCGGCGCTGGGTGATGACCTGGTGGAGAAGTTGAAGGCCGCGCAGGCCGCCCAGCG
GCAACGCATCGAGGCAGAAGCACGCCCCGGTGAATCGTGGCAAGCGGCCGCTGATCGAATCCGCA
AAGAATCCCGGCAACCGCCGGCAGCCGGTGCGCCGTCGATTAGGAAGCCGCCCAAGGGCGACGAG

CAACCAGATTTTTTCGTTCCGATGCTCTATGACGTGGGCACCCGCGATAGTCGCAGCATCATGGA
CGTGGCCGTTTTCCGTCTGTCGAAGCGTGACCGACGAGCTGGCGAGGTGATCCGCTACGAGCTTC
CAGACGGGCACGTAGAGGTTTCCGCAGGGCCGGCCGGCATGGCCAGTGTGTGGGATTACGACCTG
GTACTGATGGCGGTTTCCCATCTAACCGAATCCATGAACCGATACCGGGAAGGGAAGGGAGACAA
GCCCGGCCGCGTGTTCCGTCCACACGTTGCGGACGTACTCAAGTTCTGCCGGCGAGCCGATGGCG
GAAAGCAGAAAGACGACCTGGTAGAAACCTGCATTCGGTTAAACACCACGCACGTTGCCATGCAG
CGTACGAAGAAGGCCAAGAACGGCCGCCTGGTGACGGTATCCGAGGGTGAAGCCTTGATTAGCCG
CTACAAGATCGTAAAGAGCGAAACCGGGCGGCCGGAGTACATCGAGATCGAGCTAGCTGATTGGA
TGTACCGCGAGATCACAGAAGGCAAGAACCCGGACGTGCTGACGGTTCACCCCGATTACTTTTTG
ATCGATCCCGGCATCGGCCGTTTTCTCTACCGCCTGGCACGCCGCGCCGCAGGCAAGGCAGAAGC
CAGATGGTTGTTCAAGACGATCTACGAACGCAGTGGCAGCGCCGGAGAGTTCAAGAAGTTCTGTT
TCACCGTGCGCAAGCTGATCGGGTCAAATGACCTGCCGGAGTACGATTTGAAGGAGGAGGCGGGG
CAGGCTGGCCCGATCCTAGTCATGCGCTACCGCAACCTGATCGAGGGCGAAGCATCCGCCGGTTC
CTAATGTACGGAGCAGATGCTAGGGCAAATTGCCCTAGCAGGGGAAAAAGGTCGAAAAGGTCTCT
TTCCTGTGGATAGCACGTACATTGGGAACCCAAAGCCGTACATTGGGAACCGGAACCCGTACATT
GGGAACCCAAAGCCGTACATTGGGAACCGGTCACACATGTAAGTGACTGATATAAAAGAGAAAAA
AGGCGATTTTTCCGCCTAAAACTCTTTAAAACTTATTAAAACTCTTAAAACCCGCCTGGCCTGTG
CATAACTGTCTGGCCAGCGCACAGCCGAAGAGCTGCAAAAAGCGCCTACCCTTCGGTCGCTGCGC
TCCCTACGCCCCGCCGCTTCGCGTCGGCCTATCGCGGCCGCTGGCCGCTCAAAAATGGCTGGCCT
ACGGCCAGGCAATCTACCAGGGCGCGGACAAGCCGCGCCGTCGCCACTCGACCGCCGGCGCCCAC
ATCAAGGCACCCTGCCTCGCGCGTTTCGGTGATGACGGTGAAAACCTCTGACACATGCAGCTCCC
GGAGACGGTCACAGCTTGTCTGTAAGCGGATGCCGGGAGCAGACAAGCCCGTCAGGGCGCGTCAG
CGGGTGTTGGCGGGTGTCGGGGCGCAGCCATGACCCAGTCACGTAGCGATAGCGGAGTGTATACT
GGCTTAACTATGCGGCATCAGAGCAGATTGTACTGAGAGTGCACCATATGCGGTGTGAAATACCG
CACAGATGCGTAAGGAGAAAATACCGCATCAGGCGCTCTTCCGCTTCCTCGCTCACTGACTCGCT
GCGCTCGGTCGTTCGGCTGCGGCGAGCGGTATCAGCTCACTCAAAGGCGGTAATACGGTTATCCA
CAGAATCAGGGGATAACGCAGGAAAGAACATGTGAGCAAAAGGCCAGCAAAAGGCCAGGAACCGT
AAAAAGGCCGCGTTGCTGGCGTTTTTCCATAGGCTCCGCCCCCCTGACGAGCATCACAAAAATCG
ACGCTCAAGTCAGAGGTGGCGAAACCCGACAGGACTATAAAGATACCAGGCGTTTCCCCCTGGAA
GCTCCCTCGTGCGCTCTCCTGTTCCGACCCTGCCGCTTACCGGATACCTGTCCGCCTTTCTCCCT
TCGGGAAGCGTGGCGCTTTCTCATAGCTCACGCTGTAGGTATCTCAGTTCGGTGTAGGTCGTTCG
CTCCAAGCTGGGCTGTGTGCACGAACCCCCCGTTCAGCCCGACCGCTGCGCCTTATCCGGTAACT
ATCGTCTTGAGTCCAACCCGGTAAGACACGACTTATCGCCACTGGCAGCAGCCACTGGTAACAGG
ATTAGCAGAGCGAGGTATGTAGGCGGTGCTACAGAGTTCTTGAAGTGGTGGCCTAACTACGGCTA
CACTAGAAGGACAGTATTTGGTATCTGCGCTCTGCTGAAGCCAGTTACCTTCGGAAAAAGAGTTG
GTAGCTCTTGATCCGGCAAACAAACCACCGCTGGTAGCGGTGGTTTTTTTGTTTGCAAGCAGCAG
ATTACGCGCAGAAAAAAAGGATCTCAAGAAGATCCTTTGATCTTTTCTACGGGGTCTGACGCTCA
GTGGAACGAAAACTCACGTTAAGGGATTTTGGTCATGCAGGATCATGAATTAATTCTTAGAAAAA
CTCATCGAGCATCAAATGAAACTGCAATTTATTCATATCAGGATTATCAATACCATATTTTTGAA

AAAGCCGTTTCTGTAATGAAGGAGAAAACTCACCGAGGCAGTTCCATAGGATGGCAAGATCCTGG
TATCGGTCTGCGATTCCGACTCGTCCAACATCAATACAACCTATTAATTTCCCCTCGTCAAAAAT
AAGGTTATCAAGTGAGAAATCACCATGAGTGACGACTGAATCCGGTGAGAATGGCAAAAGTTTAT
GCATTTCTTTCCAGACTTGTTCAACAGGCCAGCCATTACGCTCGTCATCAAAATCACTCGCATCA
ACCAAACCGTTATTCATTCGTGATTGCGCCTGAGCGAGACGAAATACGCGATCGCTGTTAAAAGG
ACAATTACAAACAGGAATCGAATGCAACCGGCGCAGGAACACTGCCAGCGCATCAACAATATTTT
CACCTGAATCAGGATATTCTTCTAATACCTGGAATGCTGTTTTCCCGGGGATCGCAGTGGTGAGT
AACCATGCATCATCAGGAGTACGGATAAAATGCTTGATGGTCGGAAGAGGCATAAATTCCGTCAG
CCAGTTTAGTCTGACCATCTCATCTGTAACATCATTGGCAACGCTACCTTTGCCATGTTTCAGAA
ACAACTCTGGCGCATCGGGCTTCCCATACAATCGATAGATTGTCGCACCTGATTGCCCGACATTA
TCGCGAGCCCATTTATACCCATATAAATCAGCATCCATGTTGGAATTTAATCGCGGCCTAGAGCA
AGACGTTTCCCGTTGAATATGGCTCATAACACCCCTTGTATTACTGTTTATGTAAGCAGACAGTT
TTATTGTTCATGATCTGGATCACAGGCAGCAACGCTCTGTCATCGTTACAATCAACATGCTACCC
TCCGCGAGATCATCCGTGTTTCAAACCCGGCAGCTTAGTTGCCGTTCTTCCGAATAGCATCGGTA
ACATGAGCAAAGTCTGCCGCCTTACAACGGCTCTCCCGCTGACGCCGTCCCGGACTGATGGGCTG
CCTGTATCGAGTGGTGATTTTGTGCCGAGCTGCCGGTCGGGGAGCTGTTGGCTGGCTGGTGGCAG
GATATATTGTGGTGTAAACAAATTGACGCTTAGACAACTTAATAACACATTGCGGACGTTTTTAA
TGTACTGAATTAACGCCGAATTGCTCTAGCCAATACGCAAACCGCCTCTCCCCGCGCGTTGGCCG
ATTCATTAATGCAGCTGGCACGACAGGTTTCCCGACTGGAAAGCGGGCAGTGAGCGCAACGCAAT
TAATGTGAGTTAGCTCACTCATTAGGCACCCCAGGCTTTACACTTTATGCTTCCGGCTCGTATGT
TGTGTGGAATTGTGAGCGGATAACAATTTCACACAGGAAACAGCTATGACATGATTACGAATTCT
TAATTAAGCTACCCGGGGATCAATTCCCGATCTAGTAACATAGATGACACCGCGCGCGATAATTT
ATCCTAGTTTGCGCGCTATATTTTGTTTTCTATCGCGTATTAAATGTATAATTGCGGGACTCTAA
TCATAAAAACCCATCTCATAAATAACGTCATGCATTACATGTTAATTATTACATGCTTAACGTAA
TTCAACAGAAATTATATGATAATCATCGCAAGACCGGCAACAGGATTCAATCTTAAGAAACTTTA
TTGCCAAATGTTTGAACGATCGGGGAAATTCGAGCTTCGTGGATCCCGGTCGGCATCTACTTCAA
ATCTCGGTGACGGGCAGGACCGGACGGGGCGGTACCGGCAGGCTGAAGTCCAGCTGCCAGAAACC
CACGTCATGCCAGTTCCCGTGCTTGAAGCCGGCCGCCCGCAGCATGCCGCGGGGGGCATATCCGA
GCGCCTCGTGCATGCGCACGCTCGGGTCGTTGGGCAGCCCGATGACAGCGACCACGCTCTTGAAG
CCCTGTGCCTCCAGGGACTTCAGCAGGTGGGTGTAGAGCGTGGAGCCCAGTCCCGTCCGCTGGTG
GCGGGGGGAGACGTACACGGTCGACTCGGCCGTCCAGTCGTAGGCGTTGCGTGCCTTCCAGGGGC
CCGCGTAGGCGATGCCGGCGACCTCGCCGTCCACCTCGGCGACGAGCCAGGGATAGCGCTCCCGC
AGACGGACGAGGTCGTCCGTCCACTCCTGCGGTTCCTGCGGCTCGGTACGGAAGTTGACCGTGCT
TGTCTCGATGTAGTGGTTGACGATGGTGCAGACCGCCGGCATGTCCGCCTCGGTGGCACGGCGGA
TGTCGGCCGGGCGTCGTTCTGGGCTCATATCTTATTGCCCCCCTAGAGTCGAGATCCTTCGCCTG
GAGGAGAGAAATCAGTGGCGCTGCGGCTTTTAGGGTTTCTTTGTTGATGGAATGAGAGTGTAAGC
TCTGCCAGTGCCACTTTATTAGGGTTTTACAAGCCCTTTTCTTCGTAATTGGGCCTGACATTTTG
TGCCACTTGGGCCTTTAGAGATGAAAATGTATATTGGGCTTAAGTTGACTTGAAGGATAAATTAG
TTTAGGATATTACGTTTTTTATGAGAATTGGTGTGTCGGATACATGTATATGATGCATTCAAATA

TATGTATTCTAGATACATTTAAGTTTAGATACAATCTAAAATGTGTCTTTAATTACAGGACTGTA
ACTAAAATACTTAATGTAAGAAGAATATTACTCCTTTAATAGCTTTTGAGTATATCTAGTCTAAC
ATCTTTTAAAAAAGTCTAATTTCTTTCATTTATTTTTCGAGCAATAGCAAAGTGCATAATTATTT
TTTTCTTCTAGAAATTCAGATTTGTTTCTCTAAATTTTGAGATTCTTTTCTCAATTTTGTATGTC
TAGAGAACAATGTGTATTTTTCACTCTAGTTGGTTGTTGCTTTGTTGAATGTTCTGATAAAAGTA
TATTGTTATTTCTGAAGTAGATATAAACCTTCATTTGGAAATTATACATAAATCAAAATCGTTAA
TTATCTAGATCAAGATATATGCCCTTTTCCTAATGTATTTGATACATGCACCTAATTTCACTAGA
TGTATCTTTTCTATTTTTTAAATTATGAATAGTTAATTTTTTCCATATGTGTATTTGATACATAC
TTCATGACTTTAAAAAATTAATTATATACCAGATATATGTATTTAAAATTTGTTATGTATTTAAA
GTATGTATATGATTATTCGATATTAATCTCTTCGATGAAATTTAAATCGATAACTATGTGCTTTG
GATCTGCCCACTATCGAGCTCAAGCTTGCATGCCTGCAGGTCCGATCTGAGACTTTTCAACAAAG
GGTAATATCGGGAAACCTCCTCGGATTCCATTGCCCAGCTATCTGTCACTTCATCAAAAGGACAG
TAGAAAAGGAAGGTGGCACCTACAAATGCCATCATTGCGATAAAGGAAAGGCTATCGTTCAAGAT
GCCTCTGCCGACAGTGGTCCCAAAGATGGACCCCCACCCACGAGGAGCATCGTGGAAAAAGAAGA
CGTTCCAACCACGTCTTCAAAGCAAGTGGATTGATGTGATGGTCCGATTGAGACTTTTCAACAAA
GGGTAATATCGGGAAACCTCCTCGGATTCCATTGCCCAGCTATCTGTCACTTCATCAAAAGGACA
GTAGAAAAGGAAGGTGGCACCTACAAATGCCATCATTGCGATAAAGGAAAGGCTATCGTTCAAGA
TGCCTCTGCCGACAGTGGTCCCAAAGATGGACCCCCACCCACGAGGAGCATCGTGGAAAAAGAAG
ACGTTCCAACCACGTCTTCAAAGCAAGTGGATTGATGTGATATCTCCACTGACGTAAGGGATGAC
GCACAATCCCACTATCCTTCGCAAGACCCTTCCTCTATATAAGGAAGTTCATTTCATTTGGAGAG
GAGATCTTTTTATTTTTAATTTTCTTTCAAATACTTCCACCATGGCTCTAGAGGATCCACCGGTC
GCCACCATGGTGAGCAAGGGCGAGGAGCTGTTCACCGGGGTGGTGCCCATCCTGGTCGAGCTGGA
CGGCGACGTAAACGGCCACAAGTTCAGCGTGTCCGGCGAGGGCGAGGGCGATGCCACCTACGGCA
AGCTGACCCTGAAGTTCATCTGCACCACCGGCAAGCTGCCCGTGCCCTGGCCCACCCTCGTGACC
ACCCTGACCTACGGCGTGCAGTGCTTCAGCCGCTACCCCGACCACATGAAGCAGCACGACTTCTT
CAAGTCCGCCATGCCCGAAGGCTACGTCCAGGAGCGCACCATCTTCTTCAAGGACGACGGCAACT
ACAAGACCCGCGCCGAGGTGAAGTTCGAGGGCGACACCCTGGTGAACCGCATCGAGCTGAAGGGC
ATCGACTTCAAGGAGGACGGCAACATCCTGGGGCACAAGCTGGAGTACAACTACAACAGCCACAA
CGTCTATATCATGGCCGACAAGCAGAAGAACGGCATCAAGGTGAACTTCAAGATCCGCCACAACA
TCGAGGACGGCAGCGTGCAGCTCGCCGACCACTACCAGCAGAACACCCCCATCGGCGACGGCCCC
GTGCTGCTGCCCGACAACCACTACCTGAGCACCCAGTCCGCCCTGAGCAAAGACCCCAACGAGAA
GCGCGATCACATGGTCCTGCTGGAGTTCGTGACCGCCGCCGGGATCACTCTCGGCATGGACGAGC
TGTACAAGTAAAGCGGCCGCATGCTAGCCTAGGATCCGCGGATATCTGCAGAAGCTTCCCATGGT
GACGTCACCGGTTCTAGATACCTAGGTGAGCTCGAATTTCCCCGATCGTTCAAACATTTGGCAAT
AAAGTTTCTTAAGATTGAATCCTGTTGCCGGTCTTGCGATGATTATCATATAATTTCTGTTGAAT
TACGTTAAGCATGTAATAATTAACATGTAATGCATGACGTTATTTATGAGATGGGTTTTTATGAT
TAGAGTCCCGCAATTATACATTTAATACGCGATAGAAAACAAAATATAGCGCGCAAACTAGGATA
AATTATCGCGCGCGGTGTCATCTATGTTACTAGATCGGGAATTGATCCCCGGGTAGGTGAGACTT
TTCAACAAAGGGTAATATCGGGAAACCTCCTCGGATTCCATTGCCCAGCTATCTGTCACTTCATC

AAAAGGACAGTAGAAAAGGAAGGTGGCACCTACAAATGCCATCATTGCGATAAAGGAAAGGCTAT
CGTTCAAGATGCCCCTGCCGACAGTGGTCCCAAAGATGGACCCCCACCCACGAGGAGCATCGTGG
AAAAAGAAGACGTTCCAACCACGTCTTCAAAGCAAGTGGATTGATGTGATATCTCCACTGACGTA
AGGGATGACGCACAATCCCACTATCCTTCGCAAGACCCTTCCTCTATATAAGGAAGTTCATTTCA
TTTGGAGAGGACTCCGGTATTTTTACAACAATTACCACAACAAAACAAACAACAAACAACATTAC
AATTTACTATTCTAGTCGAAATGCCCAAGAAGAAGAGGAAGGTGGACAAGAAGTACTCCATTGGG
CTCGCTATCGGCACAAACAGCGTCGGCTGGGCCGTCATTACGGACGAGTACAAGGTGCCGAGCAA
AAAATTCAAAGTTCTGGGCAATACCGATCGCCACAGCATAAAGAAGAACCTCATTGGCGCCCTCC
TGTTCGACTCCGGGGAAACGGCCGAAGCCACGCGGCTCAAAAGAACAGCACGGCGCAGATATACC
CGCAGAAAGAATCGGATCTGCTACCTGCAGGAGATCTTTAGTAATGAGATGGCTAAGGTGGATGA
CTCTTTCTTCCATAGGCTGGAGGAGTCCTTTTTGGTGGAGGAGGATAAAAAGCACGAGCGCCACC
CAATCTTTGGCAATATCGTGGACGAGGTGGCGTACCATGAAAAGTACCCAACCATATATCATCTG
AGGAAGAAGCTTGTAGACAGTACTGATAAGGCTGACTTGCGGTTGATCTATCTCGCGCTGGCGCA
TATGATCAAATTTCGGGGACACTTCCTCATCGAGGGGGACCTGAACCCAGACAACAGCGATGTCG
ACAAACTCTTTATCCAACTGGTTCAGACTTACAATCAGCTTTTCGAAGAGAACCCGATCAACGCA
TCCGGAGTTGACGCCAAAGCAATCCTGAGCGCTAGGCTGTCCAAATCCCGGCGGCTCGAAAACCT
CATCGCACAGCTCCCTGGGGAGAAGAAGAACGGCCTGTTTGGTAATCTTATCGCCCTGTCACTCG
GGCTGACCCCCAACTTTAAATCTAACTTCGACCTGGCCGAAGATGCCAAGCTTCAACTGAGCAAA
GACACCTACGATGATGATCTCGACAATCTGCTGGCCCAGATCGGCGACCAGTACGCAGACCTTTT
TTTGGCGGCAAAGAACCTGTCAGACGCCATTCTGCTGAGTGATATTCTGCGAGTGAACACGGAGA
TCACCAAAGCTCCGCTGAGCGCTAGTATGATCAAGCGCTATGATGAGCACCACCAAGACTTGACT
TTGCTGAAGGCCCTTGTCAGACAGCAACTGCCTGAGAAGTACAAGGAAATTTTCTTCGATCAGTC
TAAAAATGGCTACGCCGGATACATTGACGGCGGAGCAAGCCAGGAGGAATTTTACAAATTTATTA
AGCCCATCTTGGAAAAAATGGACGGCACCGAGGAGCTGCTGGTAAAGCTTAACAGAGAAGATCTG
TTGCGCAAACAGCGCACTTTCGACAATGGAAGCATCCCCCACCAGATTCACCTGGGCGAACTGCA
CGCTATCCTCAGGCGGCAAGAGGATTTCTACCCCTTTTTGAAAGATAACAGGGAAAAGATTGAGA
AAATCCTCACATTTCGGATACCCTACTATGTAGGCCCCCTCGCCCGGGGAAATTCCAGATTCGCG
TGGATGACTCGCAAATCAGAAGAGACTATCACTCCCTGGAACTTCGAGGAAGTCGTGGATAAGGG
GGCCTCTGCCCAGTCCTTCATCGAAAGGATGACTAACTTTGATAAAAATCTGCCTAACGAAAAGG
TGCTTCCTAAACACTCTCTGCTGTACGAGTACTTCACAGTTTATAACGAGCTCACCAAGGTCAAA
TACGTCACAGAAGGGATGAGAAAGCCAGCATTCCTGTCTGGAGAGCAGAAGAAAGCTATCGTGGA
CCTCCTCTTCAAGACGAACCGGAAAGTTACCGTGAAACAGCTCAAAGAAGATTATTTCAAAAAGA
TTGAATGTTTCGACTCTGTTGAAATCAGCGGAGTGGAGGATCGCTTCAACGCATCCCTGGGAACG
TATCACGATCTCCTGAAAATCATTAAAGACAAGGACTTCCTGGACAATGAGGAGAACGAGGACAT
TCTTGAGGACATTGTCCTCACCCTTACGTTGTTTGAAGATAGGGAGATGATTGAAGAACGCTTGA
AAACTTACGCTCATCTCTTCGACGACAAAGTCATGAAACAGCTCAAGAGGCGCCGATATACAGGA
TGGGGGCGGCTGTCAAGAAAACTGATCAATGGGATCCGAGACAAGCAGAGTGGAAAGACAATCCT
GGATTTTCTTAAGTCCGATGGATTTGCCAACCGGAACTTCATGCAGTTGATCCATGATGACTCTC
TCACCTTTAAGGAGGACATCCAGAAAGCACAAGTTTCTGGCCAGGGGGACAGTCTCCACGAGCAC

ATCGCTAATCTTGCAGGTAGCCCAGCTATCAAAAAGGGAATACTGCAGACCGTTAAGGTCGTGGA
TGAACTCGTCAAAGTAATGGGAAGGCATAAGCCCGAGAATATCGTTATCGAGATGGCCCGAGAGA
ACCAAACTACCCAGAAGGGACAGAAGAACAGTAGGGAAAGGATGAAGAGGATTGAAGAGGGTATA
AAAGAACTGGGGTCCCAAATCCTTAAGGAACACCCAGTTGAAAACACCCAGCTTCAGAATGAGAA
GCTCTACCTGTACTACCTGCAGAACGGCAGGGACATGTACGTGGATCAGGAACTGGACATCAATC
GGCTCTCCGACTACGACGTGGATGCCATCGTGCCCCAGTCTTTTCTCAAAGATGATTCTATTGAT
AATAAAGTGTTGACAAGATCCGATAAAAATAGAGGGAAGAGTGATAACGTCCCCTCAGAAGAAGT
TGTCAAGAAAATGAAAAATTATTGGCGGCAGCTGCTGAACGCCAAACTGATCACACAACGGAAGT
TCGATAATCTGACTAAGGCTGAACGAGGTGGCCTGTCTGAGTTGGATAAAGCCGGCTTCATCAAA
AGGCAGCTTGTTGAGACACGCCAGATCACCAAGCACGTGGCCCAAATTCTCGATTCACGCATGAA
CACCAAGTACGATGAAAATGACAAACTGATTCGAGAGGTGAAAGTTATTACTCTGAAGTCTAAGC
TGGTTTCAGATTTCAGAAAGGACTTTCAGTTTTATAAGGTGAGAGAGATCAACAATTACCACCAT
GCGCATGATGCCTACCTGAATGCAGTGGTAGGCACTGCACTTATCAAAAAATATCCCAAGCTTGA
ATCTGAATTTGTTTACGGAGACTATAAAGTGTACGATGTTAGGAAAATGATCGCAAAGTCTGAGC
AGGAAATAGGCAAGGCCACCGCTAAGTACTTCTTTTACAGCAATATTATGAATTTTTTCAAGACC
GAGATTACACTGGCCAATGGAGAGATTCGGAAGCGACCACTTATCGAAACAAACGGAGAAACAGG
AGAAATCGTGTGGGACAAGGGTAGGGATTTCGCGACAGTCCGGAAGGTCCTGTCCATGCCGCAGG
TGAACATCGTTAAAAAGACCGAAGTACAGACCGGAGGCTTCTCCAAGGAAAGTATCCTCCCGAAA
AGGAACAGCGACAAGCTGATCGCACGCAAAAAAGATTGGGACCCCAAGAAATACGGCGGATTCGA
TTCTCCTACAGTCGCTTACAGTGTACTGGTTGTGGCCAAAGTGGAGAAAGGGAAGTCTAAAAAAC
TCAAAAGCGTCAAGGAACTGCTGGGCATCACAATCATGGAGCGATCAAGCTTCGAAAAAAACCCC
ATCGACTTTCTCGAGGCGAAAGGATATAAAGAGGTCAAAAAAGACCTCATCATTAAGCTTCCCAA
GTACTCTCTCTTTGAGCTTGAAAACGGCCGGAAACGAATGCTCGCTAGTGCGGGCGAGCTGCAGA
AAGGTAACGAGCTGGCACTGCCCTCTAAATACGTTAATTTCTTGTATCTGGCCAGCCACTATGAA
AAGCTCAAAGGATCTCCCGAAGATAATGAGCAGAAGCAGCTGTTCGTGGAACAACACAAACACTA
CCTTGATGAGATCATCGAGCAAATAAGCGAATTCTCCAAAAGAGTGATCCTCGCCGACGCTAACC
TCGATAAGGTGCTTTCTGCTTACAATAAGCACAGGGATAAGCCCATCAGGGAGCAGGCAGAAAAC
ATTATCCACTTGTTTACTCTGACCAACTTGGGCGCGCCTGCAGCCTTCAAGTACTTCGACACCAC
CATAGACAGAAAGCGGTACACCTCTACAAAGGAGGTCCTGGACGCCACACTGATTCATCAGTCAA
TTACGGGGCTCTATGAAACAAGAATCGACCTCTCTCAGCTCGGTGGAGACAGCAGGGCTGATTCG
GACCCGAAGAAAAAGAGGAAGGTAGATCCTAAGAAGAAGAGAAAGGTACTGTTGGATCCGGGAAC
ACCTATGGACGCTGATTTGGTAGCTTCTTCTACAGTAGTGTGGGAACAAGATGCAGATCCTTTTG
CTGGAACAGCCGACGACTTCCCCGCCTTCAATGAAGAGGAGCTTGCATGGTTGATGGAGCTGTTA
CCTCAAGGAGGATCAGGGGGTCTTTTAGATCCAGGTACTCCCATGGATGCAGACCTGGTCGCTTC
AAGTACAGTCGTTTGGGAGCAGGATGCTGATCCATTCGCAGGTACAGCAGATGACTTCCCGGCTT
TCAATGAGGAGGAACTTGCTTGGCTCATGGAATTGCTCCCGCAGGCAAGAGGTGGATCTGGTGGT
CTGCTCGACCCAGGAACGCCGATGGACGCTGATCTGGTAGCATCTAGTACAGTCTGGGAGCAGGA
CGCTGACCCATTCGCTGGTACGGCTGACGATTTTCCTGCCTTTAATGAGGAAGAGTTAGCCTGGC
TGATGGAGTTATTACCCCAGGGCGGTAGTGGCGGATTATTGGACCCTGGTACGCCGATGGATGCA

GACCTTGTGGCTTCATCAACGGTCGTATGGGAGCAGGATGCCGATCCTTTCGCAGGAACCGCAGA
CGACTTCCCAGCCTTTAACGAAGAAGAGCTCGCATGGCTTATGGAACTTTTACCTCAGGCACGTG
GAGGAAGTGGTGGGCTCCTGGATCCAGGCACTCCGATGGACGCAGACTTAGTAGCTAGTTCCACC
GTTGTCTGGGAGCAGGATGCAGATCCTTTTGCTGGTACCGCAGACGACTTCCCTGCTTTCAATGA
AGAAGAATTGGCCTGGCTTATGGAATTACTTCCGCAGGGTGGGTCTGGTGGGCTGCTCGATCCCG
GTACGCCGATGGATGCTGACTTGGTTGCTAGCAGTACTGTGGTCTGGGAACAGGATGCTGACCCC
TTCGCTGGGACTGCCGACGACTTCCCAGCCTTCAACGAAGAGGAACTCGCATGGTTGATGGAACT
GCTTCCTCAAGCACGTGGGGGGTCAGGCGGAGGCGGATCTGGCGGTGACGCACTGGACGACTTTG
ATCTCGACATGCTTGGGTCCGATGCTCTCGACGATTTTGACCTCGATATGCTTGGGTCTGACGCA
TTAGATGACTTTGATTTAGACATGTTGGGGTCAGACGCTTTGGACGATTTCGACTTAGATATGTT
GGCCAGAGGTAGTGATGCCCTTGACGACTTCGATTTAGATATGTTGGGCTCTGACGCCCTCGATG
ACTTCGATCTGGATATGCTCGGAAGTGACGCCTTGGACGATTTCGACTTGGACATGCTTGGGTCG
GACGCCCTCGACGACTTTGATCTGGATATGTTATGAGCTTGGAATGGATCTTCGATCCCGATCGT
TCAAACATTTGGCAATAAAGTTTCTTAAGATTGAATCCTGTTGCCGGTCTTGCGACGATTATCAT
ATAATTTCTGTTGAATTACGTTAAGCATGTAATAATTAACATGTAATGCATGACGTTATTTATGA
GATGGGTTTTTATGATTAGAGTCCCGCAATTATACATTTAATACGCGATAGAAAACAAAATATAG
CGCGCAAACTAGGATAAATTATCGCGCGCGGTGTCATCTATGTTACTAGATCGGGAATTGCCAAG
CTAATTCCTTCGTTGAACAACGGAAACTCGACTTGCCTTCCGCACAATACATCATTTCTTCTTAG
CTTTTTTTCTTCTTCTTCGTTCATACAGTTTTTTTTTGTTTATCAGCTTACATTTTCTTGAACCG
TAGCTTTCGTTTTCTTCTTTTTAACTTTCCATTCGGAGTTTTTGTATCTTGTTTCATAGTTTGTC
CCAGGATTAGAATGATTAGGCATCGAACCTTCAAGAATTTGATTGAATAAAACATCTTCATTCTT
AAGATATGAAGATAATCTTCAAAAGGCCCCTGGGAATCTGAAAGAAGAGAAGCAGGCCCATTTAT
ATGGGAAAGAACAATAGTATTTCTTATATAGGCCCATTTAAGTTGAAAACAATCTTCAAAAGTCC
CACATCGCTTAGATAAGAAAACGAAGCTGAGTTTATATACAGCTAGAGTCGAAGTAGTGATTGAT
AAGGTTTGTGGAAACGCGTTTTAGAGCTAGAAATAGCAAGTTAAAATAAGGCTAGTCCGTTATCA
ACTTGAAAAAGTGGCACCGAGTCGGTGCTTTTTTTTTGAAGACGAAAGGGCCTCGTGATACGCCT
ATTTTTATAGGTTAATGTCATGATAATAATGGTTTCTTAGACGTCAGGTGGCACTTTTCGGGGAA
ATGTGCGCGGAACCCCTATTTGTTTATTTTTCTAAATACATTCAAATATGTATCCGCTCATGAGA
CAATAACCCTGATAAATGCTTCAATAATGGGACCGACTCGCGCTTGAGACGAAGCTCCAAGCTTG
GCACTGGCCGTCGTTTTACAACGTCGTGACTGGGAAAACCCTGGCGTTACCCAACTTAATCGCCT
TGCAGCACATCCCCCTTTCGCCAGCTGGCGTAATAGCGAAGAGGCCCGCACCGATCGCCCTTCCC
AACAGTTGCGCAGCCTGAATGGCGAATGAGCTTGAGCTTGGATCAGATTGTCGTTTCCCGCCTTC
AGTTTAAACTATCAGTGTTTGACAGGATATATTGGCGGGTAAACCTAAGAGAAAAGAGCGTTTAT
TAGAATAACGGATATTTAAAAGGGCGTGAAAAGGTTTATCCGTTCGTCCATTTGTATGTGCATGC
CAACCACAGGGTTCCCCTCGGGATCAA3’

Supplementary File 4. pPZP_CRISPRa sequence.

5’AGTACTTTGATCCAACCCCTCCGCTGCTATAGTGCAGTCGGCTTCTGACGTTCAGTGCAGCCG
TCTTCTGAAAACGACATGTCGCACAAGTCCTAAGTTACGCGACAGGCTGCCGCCCTGCCCTTTTC
CTGGCGTTTTCTTGTCGCGTGTTTTAGTCGCATAAAGTAGAATACTTGCGACTAGAACCGGAGAC
ATTACGCCATGAACAAGAGCGCCGCCGCTGGCCTGCTGGGCTATGCCCGCGTCAGCACCGACGAC
CAGGACTTGACCAACCAACGGGCCGAACTGCACGCGGCCGGCTGCACCAAGCTGTTTTCCGAGAA
GATCACCGGCACCAGGCGCGACCGCCCGGAGCTGGCCAGGATGCTTGACCACCTACGCCCTGGCG
ACGTTGTGACAGTGACCAGGCTAGACCGCCTGGCCCGCAGCACCCGCGACCTACTGGACATTGCC
GAGCGCATCCAGGAGGCCGGCGCGGGCCTGCGTAGCCTGGCAGAGCCGTGGGCCGACACCACCAC
GCCGGCCGGCCGCATGGTGTTGACCGTGTTCGCCGGCATTGCCGAGTTCGAGCGTTCCCTAATCA
TCGACCGCACCCGGAGCGGGCGCGAGGCCGCCAAGGCCCGAGGCGTGAAGTTTGGCCCCCGCCCT
ACCCTCACCCCGGCACAGATCGCGCACGCCCGCGAGCTGATCGACCAGGAAGGCCGCACCGTGAA
AGAGGCGGCTGCACTGCTTGGCGTGCATCGCTCGACCCTGTACCGCGCACTTGAGCGCAGCGAGG
AAGTGACGCCCACCGAGGCCAGGCGGCGCGGTGCCTTCCGTGAGGACGCATTGACCGAGGCCGAC
GCCCTGGCGGCCGCCGAGAATGAACGCCAAGAGGAACAAGCATGAAACCGCACCAGGACGGCCAG
GACGAACCGTTTTTCATTACCGAAGAGATCGAGGCGGAGATGATCGCGGCCGGGTACGTGTTCGA
GCCGCCCGCGCACGTCTCAACCGTGCGGCTGCATGAAATCCTGGCCGGTTTGTCTGATGCCAAGC
TGGCGGCCTGGCCGGCCAGCTTGGCCGCTGAAGAAACCGAGCGCCGCCGTCTAAAAAGGTGATGT
GTATTTGAGTAAAACAGCTTGCGTCATGCGGTCGCTGCGTATATGATGCGATGAGTAAATAAACA
AATACGCAAGGGGAACGCATGAAGGTTATCGCTGTACTTAACCAGAAAGGCGGGTCAGGCAAGAC
GACCATCGCAACCCATCTAGCCCGCGCCCTGCAACTCGCCGGGGCCGATGTTCTGTTAGTCGATT
CCGATCCCCAGGGCAGTGCCCGCGATTGGGCGGCCGTGCGGGAAGATCAACCGCTAACCGTTGTC
GGCATCGACCGCCCGACGATTGACCGCGACGTGAAGGCCATCGGCCGGCGCGACTTCGTAGTGAT
CGACGGAGCGCCCCAGGCGGCGGACTTGGCTGTGTCCGCGATCAAGGCAGCCGACTTCGTGCTGA
TTCCGGTGCAGCCAAGCCCTTACGACATATGGGCCACCGCCGACCTGGTGGAGCTGGTTAAGCAG
CGCATTGAGGTCACGGATGGAAGGCTACAAGCGGCCTTTGTCGTGTCGCGGGCGATCAAAGGCAC
GCGCATCGGCGGTGAGGTTGCCGAGGCGCTGGCCGGGTACGAGCTGCCCATTCTTGAGTCCCGTA
TCACGCAGCGCGTGAGCTACCCAGGCACTGCCGCCGCCGGCACAACCGTTCTTGAATCAGAACCC
GAGGGCGACGCTGCCCGCGAGGTCCAGGCGCTGGCCGCTGAAATTAAATCAAAACTCATTTGAGT
TAATGAGGTAAAGAGAAAATGAGCAAAAGCACAAACACGCTAAGTGCCGGCCGTCCGAGCGCACG
CAGCAGCAAGGCTGCAACGTTGGCCAGCCTGGCAGACACGCCAGCCATGAAGCGGGTCAACTTTC
AGTTGCCGGCGGAGGATCACACCAAGCTGAAGATGTACGCGGTACGCCAAGGCAAGACCATTACC
GAGCTGCTATCTGAATACATCGCGCAGCTACCAGAGTAAATGAGCAAATGAATAAATGAGTAGAT
GAATTTTAGCGGCTAAAGGAGGCGGCATGGAAAATCAAGAACAACCAGGCACCGACGCCGTGGAA
TGCCCCATGTGTGGAGGAACGGGCGGTTGGCCAGGCGTAAGCGGCTGGGTTGTCTGCCGGCCCTG
CAATGGCACTGGAACCCCCAAGCCCGAGGAATCGGCGTGACGGTCGCAAACCATCCGGCCCGGTA
CAAATCGGCGCGGCGCTGGGTGATGACCTGGTGGAGAAGTTGAAGGCCGCGCAGGCCGCCCAGCG
GCAACGCATCGAGGCAGAAGCACGCCCCGGTGAATCGTGGCAAGCGGCCGCTGATCGAATCCGCA
AAGAATCCCGGCAACCGCCGGCAGCCGGTGCGCCGTCGATTAGGAAGCCGCCCAAGGGCGACGAG

CAACCAGATTTTTTCGTTCCGATGCTCTATGACGTGGGCACCCGCGATAGTCGCAGCATCATGGA
CGTGGCCGTTTTCCGTCTGTCGAAGCGTGACCGACGAGCTGGCGAGGTGATCCGCTACGAGCTTC
CAGACGGGCACGTAGAGGTTTCCGCAGGGCCGGCCGGCATGGCCAGTGTGTGGGATTACGACCTG
GTACTGATGGCGGTTTCCCATCTAACCGAATCCATGAACCGATACCGGGAAGGGAAGGGAGACAA
GCCCGGCCGCGTGTTCCGTCCACACGTTGCGGACGTACTCAAGTTCTGCCGGCGAGCCGATGGCG
GAAAGCAGAAAGACGACCTGGTAGAAACCTGCATTCGGTTAAACACCACGCACGTTGCCATGCAG
CGTACGAAGAAGGCCAAGAACGGCCGCCTGGTGACGGTATCCGAGGGTGAAGCCTTGATTAGCCG
CTACAAGATCGTAAAGAGCGAAACCGGGCGGCCGGAGTACATCGAGATCGAGCTAGCTGATTGGA
TGTACCGCGAGATCACAGAAGGCAAGAACCCGGACGTGCTGACGGTTCACCCCGATTACTTTTTG
ATCGATCCCGGCATCGGCCGTTTTCTCTACCGCCTGGCACGCCGCGCCGCAGGCAAGGCAGAAGC
CAGATGGTTGTTCAAGACGATCTACGAACGCAGTGGCAGCGCCGGAGAGTTCAAGAAGTTCTGTT
TCACCGTGCGCAAGCTGATCGGGTCAAATGACCTGCCGGAGTACGATTTGAAGGAGGAGGCGGGG
CAGGCTGGCCCGATCCTAGTCATGCGCTACCGCAACCTGATCGAGGGCGAAGCATCCGCCGGTTC
CTAATGTACGGAGCAGATGCTAGGGCAAATTGCCCTAGCAGGGGAAAAAGGTCGAAAAGGTCTCT
TTCCTGTGGATAGCACGTACATTGGGAACCCAAAGCCGTACATTGGGAACCGGAACCCGTACATT
GGGAACCCAAAGCCGTACATTGGGAACCGGTCACACATGTAAGTGACTGATATAAAAGAGAAAAA
AGGCGATTTTTCCGCCTAAAACTCTTTAAAACTTATTAAAACTCTTAAAACCCGCCTGGCCTGTG
CATAACTGTCTGGCCAGCGCACAGCCGAAGAGCTGCAAAAAGCGCCTACCCTTCGGTCGCTGCGC
TCCCTACGCCCCGCCGCTTCGCGTCGGCCTATCGCGGCCGCTGGCCGCTCAAAAATGGCTGGCCT
ACGGCCAGGCAATCTACCAGGGCGCGGACAAGCCGCGCCGTCGCCACTCGACCGCCGGCGCCCAC
ATCAAGGCACCCTGCCTCGCGCGTTTCGGTGATGACGGTGAAAACCTCTGACACATGCAGCTCCC
GGAGACGGTCACAGCTTGTCTGTAAGCGGATGCCGGGAGCAGACAAGCCCGTCAGGGCGCGTCAG
CGGGTGTTGGCGGGTGTCGGGGCGCAGCCATGACCCAGTCACGTAGCGATAGCGGAGTGTATACT
GGCTTAACTATGCGGCATCAGAGCAGATTGTACTGAGAGTGCACCATATGCGGTGTGAAATACCG
CACAGATGCGTAAGGAGAAAATACCGCATCAGGCGCTCTTCCGCTTCCTCGCTCACTGACTCGCT
GCGCTCGGTCGTTCGGCTGCGGCGAGCGGTATCAGCTCACTCAAAGGCGGTAATACGGTTATCCA
CAGAATCAGGGGATAACGCAGGAAAGAACATGTGAGCAAAAGGCCAGCAAAAGGCCAGGAACCGT
AAAAAGGCCGCGTTGCTGGCGTTTTTCCATAGGCTCCGCCCCCCTGACGAGCATCACAAAAATCG
ACGCTCAAGTCAGAGGTGGCGAAACCCGACAGGACTATAAAGATACCAGGCGTTTCCCCCTGGAA
GCTCCCTCGTGCGCTCTCCTGTTCCGACCCTGCCGCTTACCGGATACCTGTCCGCCTTTCTCCCT
TCGGGAAGCGTGGCGCTTTCTCATAGCTCACGCTGTAGGTATCTCAGTTCGGTGTAGGTCGTTCG
CTCCAAGCTGGGCTGTGTGCACGAACCCCCCGTTCAGCCCGACCGCTGCGCCTTATCCGGTAACT
ATCGTCTTGAGTCCAACCCGGTAAGACACGACTTATCGCCACTGGCAGCAGCCACTGGTAACAGG
ATTAGCAGAGCGAGGTATGTAGGCGGTGCTACAGAGTTCTTGAAGTGGTGGCCTAACTACGGCTA
CACTAGAAGGACAGTATTTGGTATCTGCGCTCTGCTGAAGCCAGTTACCTTCGGAAAAAGAGTTG
GTAGCTCTTGATCCGGCAAACAAACCACCGCTGGTAGCGGTGGTTTTTTTGTTTGCAAGCAGCAG
ATTACGCGCAGAAAAAAAGGATCTCAAGAAGATCCTTTGATCTTTTCTACGGGGTCTGACGCTCA
GTGGAACGAAAACTCACGTTAAGGGATTTTGGTCATGCAGGATCATGAATTAATTCTTAGAAAAA
CTCATCGAGCATCAAATGAAACTGCAATTTATTCATATCAGGATTATCAATACCATATTTTTGAA

AAAGCCGTTTCTGTAATGAAGGAGAAAACTCACCGAGGCAGTTCCATAGGATGGCAAGATCCTGG
TATCGGTCTGCGATTCCGACTCGTCCAACATCAATACAACCTATTAATTTCCCCTCGTCAAAAAT
AAGGTTATCAAGTGAGAAATCACCATGAGTGACGACTGAATCCGGTGAGAATGGCAAAAGTTTAT
GCATTTCTTTCCAGACTTGTTCAACAGGCCAGCCATTACGCTCGTCATCAAAATCACTCGCATCA
ACCAAACCGTTATTCATTCGTGATTGCGCCTGAGCGAGACGAAATACGCGATCGCTGTTAAAAGG
ACAATTACAAACAGGAATCGAATGCAACCGGCGCAGGAACACTGCCAGCGCATCAACAATATTTT
CACCTGAATCAGGATATTCTTCTAATACCTGGAATGCTGTTTTCCCGGGGATCGCAGTGGTGAGT
AACCATGCATCATCAGGAGTACGGATAAAATGCTTGATGGTCGGAAGAGGCATAAATTCCGTCAG
CCAGTTTAGTCTGACCATCTCATCTGTAACATCATTGGCAACGCTACCTTTGCCATGTTTCAGAA
ACAACTCTGGCGCATCGGGCTTCCCATACAATCGATAGATTGTCGCACCTGATTGCCCGACATTA
TCGCGAGCCCATTTATACCCATATAAATCAGCATCCATGTTGGAATTTAATCGCGGCCTAGAGCA
AGACGTTTCCCGTTGAATATGGCTCATAACACCCCTTGTATTACTGTTTATGTAAGCAGACAGTT
TTATTGTTCATGATCTGGATCACAGGCAGCAACGCTCTGTCATCGTTACAATCAACATGCTACCC
TCCGCGAGATCATCCGTGTTTCAAACCCGGCAGCTTAGTTGCCGTTCTTCCGAATAGCATCGGTA
ACATGAGCAAAGTCTGCCGCCTTACAACGGCTCTCCCGCTGACGCCGTCCCGGACTGATGGGCTG
CCTGTATCGAGTGGTGATTTTGTGCCGAGCTGCCGGTCGGGGAGCTGTTGGCTGGCTGGTGGCAG
GATATATTGTGGTGTAAACAAATTGACGCTTAGACAACTTAATAACACATTGCGGACGTTTTTAA
TGTACTGAATTAACGCCGAATTGCTCTAGCCAATACGCAAACCGCCTCTCCCCGCGCGTTGGCCG
ATTCATTAATGCAGCTGGCACGACAGGTTTCCCGACTGGAAAGCGGGCAGTGAGCGCAACGCAAT
TAATGTGAGTTAGCTCACTCATTAGGCACCCCAGGCTTTACACTTTATGCTTCCGGCTCGTATGT
TGTGTGGAATTGTGAGCGGATAACAATTTCACACAGGAAACAGCTATGACATGATTACGAATTCT
TAATTAAGCTACCCGGGGATCAATTCCCGATCTAGTAACATAGATGACACCGCGCGCGATAATTT
ATCCTAGTTTGCGCGCTATATTTTGTTTTCTATCGCGTATTAAATGTATAATTGCGGGACTCTAA
TCATAAAAACCCATCTCATAAATAACGTCATGCATTACATGTTAATTATTACATGCTTAACGTAA
TTCAACAGAAATTATATGATAATCATCGCAAGACCGGCAACAGGATTCAATCTTAAGAAACTTTA
TTGCCAAATGTTTGAACGATCGGGGAAATTCGAGCTTCGTGGATCCCGGTCGGCATCTACTTCAA
ATCTCGGTGACGGGCAGGACCGGACGGGGCGGTACCGGCAGGCTGAAGTCCAGCTGCCAGAAACC
CACGTCATGCCAGTTCCCGTGCTTGAAGCCGGCCGCCCGCAGCATGCCGCGGGGGGCATATCCGA
GCGCCTCGTGCATGCGCACGCTCGGGTCGTTGGGCAGCCCGATGACAGCGACCACGCTCTTGAAG
CCCTGTGCCTCCAGGGACTTCAGCAGGTGGGTGTAGAGCGTGGAGCCCAGTCCCGTCCGCTGGTG
GCGGGGGGAGACGTACACGGTCGACTCGGCCGTCCAGTCGTAGGCGTTGCGTGCCTTCCAGGGGC
CCGCGTAGGCGATGCCGGCGACCTCGCCGTCCACCTCGGCGACGAGCCAGGGATAGCGCTCCCGC
AGACGGACGAGGTCGTCCGTCCACTCCTGCGGTTCCTGCGGCTCGGTACGGAAGTTGACCGTGCT
TGTCTCGATGTAGTGGTTGACGATGGTGCAGACCGCCGGCATGTCCGCCTCGGTGGCACGGCGGA
TGTCGGCCGGGCGTCGTTCTGGGCTCATATCTTATTGCCCCCCTAGAGTCGAGATCCTTCGCCTG
GAGGAGAGAAATCAGTGGCGCTGCGGCTTTTAGGGTTTCTTTGTTGATGGAATGAGAGTGTAAGC
TCTGCCAGTGCCACTTTATTAGGGTTTTACAAGCCCTTTTCTTCGTAATTGGGCCTGACATTTTG
TGCCACTTGGGCCTTTAGAGATGAAAATGTATATTGGGCTTAAGTTGACTTGAAGGATAAATTAG
TTTAGGATATTACGTTTTTTATGAGAATTGGTGTGTCGGATACATGTATATGATGCATTCAAATA

TATGTATTCTAGATACATTTAAGTTTAGATACAATCTAAAATGTGTCTTTAATTACAGGACTGTA
ACTAAAATACTTAATGTAAGAAGAATATTACTCCTTTAATAGCTTTTGAGTATATCTAGTCTAAC
ATCTTTTAAAAAAGTCTAATTTCTTTCATTTATTTTTCGAGCAATAGCAAAGTGCATAATTATTT
TTTTCTTCTAGAAATTCAGATTTGTTTCTCTAAATTTTGAGATTCTTTTCTCAATTTTGTATGTC
TAGAGAACAATGTGTATTTTTCACTCTAGTTGGTTGTTGCTTTGTTGAATGTTCTGATAAAAGTA
TATTGTTATTTCTGAAGTAGATATAAACCTTCATTTGGAAATTATACATAAATCAAAATCGTTAA
TTATCTAGATCAAGATATATGCCCTTTTCCTAATGTATTTGATACATGCACCTAATTTCACTAGA
TGTATCTTTTCTATTTTTTAAATTATGAATAGTTAATTTTTTCCATATGTGTATTTGATACATAC
TTCATGACTTTAAAAAATTAATTATATACCAGATATATGTATTTAAAATTTGTTATGTATTTAAA
GTATGTATATGATTATTCGATATTAATCTCTTCGATGAAATTTAAATCGATAACTATGTGCTTTG
GATCTGCCCACTATCGAGCTCAAGCTTGCATGCCTGCAGGTCCGATCTGAGACTTTTCAACAAAG
GGTAATATCGGGAAACCTCCTCGGATTCCATTGCCCAGCTATCTGTCACTTCATCAAAAGGACAG
TAGAAAAGGAAGGTGGCACCTACAAATGCCATCATTGCGATAAAGGAAAGGCTATCGTTCAAGAT
GCCTCTGCCGACAGTGGTCCCAAAGATGGACCCCCACCCACGAGGAGCATCGTGGAAAAAGAAGA
CGTTCCAACCACGTCTTCAAAGCAAGTGGATTGATGTGATGGTCCGATTGAGACTTTTCAACAAA
GGGTAATATCGGGAAACCTCCTCGGATTCCATTGCCCAGCTATCTGTCACTTCATCAAAAGGACA
GTAGAAAAGGAAGGTGGCACCTACAAATGCCATCATTGCGATAAAGGAAAGGCTATCGTTCAAGA
TGCCTCTGCCGACAGTGGTCCCAAAGATGGACCCCCACCCACGAGGAGCATCGTGGAAAAAGAAG
ACGTTCCAACCACGTCTTCAAAGCAAGTGGATTGATGTGATATCTCCACTGACGTAAGGGATGAC
GCACAATCCCACTATCCTTCGCAAGACCCTTCCTCTATATAAGGAAGTTCATTTCATTTGGAGAG
GAGATCTTTTTATTTTTAATTTTCTTTCAAATACTTCCACCATGGCTCTAGAGGATCCACCGGTC
GCCACCATGGTGAGCAAGGGCGAGGAGCTGTTCACCGGGGTGGTGCCCATCCTGGTCGAGCTGGA
CGGCGACGTAAACGGCCACAAGTTCAGCGTGTCCGGCGAGGGCGAGGGCGATGCCACCTACGGCA
AGCTGACCCTGAAGTTCATCTGCACCACCGGCAAGCTGCCCGTGCCCTGGCCCACCCTCGTGACC
ACCCTGACCTACGGCGTGCAGTGCTTCAGCCGCTACCCCGACCACATGAAGCAGCACGACTTCTT
CAAGTCCGCCATGCCCGAAGGCTACGTCCAGGAGCGCACCATCTTCTTCAAGGACGACGGCAACT
ACAAGACCCGCGCCGAGGTGAAGTTCGAGGGCGACACCCTGGTGAACCGCATCGAGCTGAAGGGC
ATCGACTTCAAGGAGGACGGCAACATCCTGGGGCACAAGCTGGAGTACAACTACAACAGCCACAA
CGTCTATATCATGGCCGACAAGCAGAAGAACGGCATCAAGGTGAACTTCAAGATCCGCCACAACA
TCGAGGACGGCAGCGTGCAGCTCGCCGACCACTACCAGCAGAACACCCCCATCGGCGACGGCCCC
GTGCTGCTGCCCGACAACCACTACCTGAGCACCCAGTCCGCCCTGAGCAAAGACCCCAACGAGAA
GCGCGATCACATGGTCCTGCTGGAGTTCGTGACCGCCGCCGGGATCACTCTCGGCATGGACGAGC
TGTACAAGTAAAGCGGCCGCATGCTAGCCTAGGATCCGCGGATATCTGCAGAAGCTTCCCATGGT
GACGTCACCGGTTCTAGATACCTAGGTGAGCTCGAATTTCCCCGATCGTTCAAACATTTGGCAAT
AAAGTTTCTTAAGATTGAATCCTGTTGCCGGTCTTGCGATGATTATCATATAATTTCTGTTGAAT
TACGTTAAGCATGTAATAATTAACATGTAATGCATGACGTTATTTATGAGATGGGTTTTTATGAT
TAGAGTCCCGCAATTATACATTTAATACGCGATAGAAAACAAAATATAGCGCGCAAACTAGGATA
AATTATCGCGCGCGGTGTCATCTATGTTACTAGATCGGGAATTGATCCCCGGGTAGGCGCGCCTG
AGACTTTTCAACAAAGGGTAATATCGGGAAACCTCCTCGGATTCCATTGCCCAGCTATCTGTCAC

TTCATCAAAAGGACAGTAGAAAAGGAAGGTGGCACCTACAAATGCCATCATTGCGATAAAGGAAA
GGCTATCGTTCAAGATGCCCCTGCCGACAGTGGTCCCAAAGATGGACCCCCACCCACGAGGAGCA
TCGTGGAAAAAGAAGACGTTCCAACCACGTCTTCAAAGCAAGTGGATTGATGTGATATCTCCACT
GACGTAAGGGATGACGCACAATCCCACTATCCTTCGCAAGACCCTTCCTCTATATAAGGAAGTTC
ATTTCATTTGGAGAGGACTCCGGTATTTTTACAACAATTACCACAACAAAACAAACAACAAACAA
CATTACAATTTACTATTCTAGTCGAAATGCCCAAGAAGAAGAGGAAGGTGGACAAGAAGTACTCC
ATTGGGCTCGCTATCGGCACAAACAGCGTCGGCTGGGCCGTCATTACGGACGAGTACAAGGTGCC
GAGCAAAAAATTCAAAGTTCTGGGCAATACCGATCGCCACAGCATAAAGAAGAACCTCATTGGCG
CCCTCCTGTTCGACTCCGGGGAAACGGCCGAAGCCACGCGGCTCAAAAGAACAGCACGGCGCAGA
TATACCCGCAGAAAGAATCGGATCTGCTACCTGCAGGAGATCTTTAGTAATGAGATGGCTAAGGT
GGATGACTCTTTCTTCCATAGGCTGGAGGAGTCCTTTTTGGTGGAGGAGGATAAAAAGCACGAGC
GCCACCCAATCTTTGGCAATATCGTGGACGAGGTGGCGTACCATGAAAAGTACCCAACCATATAT
CATCTGAGGAAGAAGCTTGTAGACAGTACTGATAAGGCTGACTTGCGGTTGATCTATCTCGCGCT
GGCGCATATGATCAAATTTCGGGGACACTTCCTCATCGAGGGGGACCTGAACCCAGACAACAGCG
ATGTCGACAAACTCTTTATCCAACTGGTTCAGACTTACAATCAGCTTTTCGAAGAGAACCCGATC
AACGCATCCGGAGTTGACGCCAAAGCAATCCTGAGCGCTAGGCTGTCCAAATCCCGGCGGCTCGA
AAACCTCATCGCACAGCTCCCTGGGGAGAAGAAGAACGGCCTGTTTGGTAATCTTATCGCCCTGT
CACTCGGGCTGACCCCCAACTTTAAATCTAACTTCGACCTGGCCGAAGATGCCAAGCTTCAACTG
AGCAAAGACACCTACGATGATGATCTCGACAATCTGCTGGCCCAGATCGGCGACCAGTACGCAGA
CCTTTTTTTGGCGGCAAAGAACCTGTCAGACGCCATTCTGCTGAGTGATATTCTGCGAGTGAACA
CGGAGATCACCAAAGCTCCGCTGAGCGCTAGTATGATCAAGCGCTATGATGAGCACCACCAAGAC
TTGACTTTGCTGAAGGCCCTTGTCAGACAGCAACTGCCTGAGAAGTACAAGGAAATTTTCTTCGA
TCAGTCTAAAAATGGCTACGCCGGATACATTGACGGCGGAGCAAGCCAGGAGGAATTTTACAAAT
TTATTAAGCCCATCTTGGAAAAAATGGACGGCACCGAGGAGCTGCTGGTAAAGCTTAACAGAGAA
GATCTGTTGCGCAAACAGCGCACTTTCGACAATGGAAGCATCCCCCACCAGATTCACCTGGGCGA
ACTGCACGCTATCCTCAGGCGGCAAGAGGATTTCTACCCCTTTTTGAAAGATAACAGGGAAAAGA
TTGAGAAAATCCTCACATTTCGGATACCCTACTATGTAGGCCCCCTCGCCCGGGGAAATTCCAGA
TTCGCGTGGATGACTCGCAAATCAGAAGAGACTATCACTCCCTGGAACTTCGAGGAAGTCGTGGA
TAAGGGGGCCTCTGCCCAGTCCTTCATCGAAAGGATGACTAACTTTGATAAAAATCTGCCTAACG
AAAAGGTGCTTCCTAAACACTCTCTGCTGTACGAGTACTTCACAGTTTATAACGAGCTCACCAAG
GTCAAATACGTCACAGAAGGGATGAGAAAGCCAGCATTCCTGTCTGGAGAGCAGAAGAAAGCTAT
CGTGGACCTCCTCTTCAAGACGAACCGGAAAGTTACCGTGAAACAGCTCAAAGAAGATTATTTCA
AAAAGATTGAATGTTTCGACTCTGTTGAAATCAGCGGAGTGGAGGATCGCTTCAACGCATCCCTG
GGAACGTATCACGATCTCCTGAAAATCATTAAAGACAAGGACTTCCTGGACAATGAGGAGAACGA
GGACATTCTTGAGGACATTGTCCTCACCCTTACGTTGTTTGAAGATAGGGAGATGATTGAAGAAC
GCTTGAAAACTTACGCTCATCTCTTCGACGACAAAGTCATGAAACAGCTCAAGAGGCGCCGATAT
ACAGGATGGGGGCGGCTGTCAAGAAAACTGATCAATGGGATCCGAGACAAGCAGAGTGGAAAGAC
AATCCTGGATTTTCTTAAGTCCGATGGATTTGCCAACCGGAACTTCATGCAGTTGATCCATGATG
ACTCTCTCACCTTTAAGGAGGACATCCAGAAAGCACAAGTTTCTGGCCAGGGGGACAGTCTCCAC

GAGCACATCGCTAATCTTGCAGGTAGCCCAGCTATCAAAAAGGGAATACTGCAGACCGTTAAGGT
CGTGGATGAACTCGTCAAAGTAATGGGAAGGCATAAGCCCGAGAATATCGTTATCGAGATGGCCC
GAGAGAACCAAACTACCCAGAAGGGACAGAAGAACAGTAGGGAAAGGATGAAGAGGATTGAAGAG
GGTATAAAAGAACTGGGGTCCCAAATCCTTAAGGAACACCCAGTTGAAAACACCCAGCTTCAGAA
TGAGAAGCTCTACCTGTACTACCTGCAGAACGGCAGGGACATGTACGTGGATCAGGAACTGGACA
TCAATCGGCTCTCCGACTACGACGTGGATGCCATCGTGCCCCAGTCTTTTCTCAAAGATGATTCT
ATTGATAATAAAGTGTTGACAAGATCCGATAAAAATAGAGGGAAGAGTGATAACGTCCCCTCAGA
AGAAGTTGTCAAGAAAATGAAAAATTATTGGCGGCAGCTGCTGAACGCCAAACTGATCACACAAC
GGAAGTTCGATAATCTGACTAAGGCTGAACGAGGTGGCCTGTCTGAGTTGGATAAAGCCGGCTTC
ATCAAAAGGCAGCTTGTTGAGACACGCCAGATCACCAAGCACGTGGCCCAAATTCTCGATTCACG
CATGAACACCAAGTACGATGAAAATGACAAACTGATTCGAGAGGTGAAAGTTATTACTCTGAAGT
CTAAGCTGGTTTCAGATTTCAGAAAGGACTTTCAGTTTTATAAGGTGAGAGAGATCAACAATTAC
CACCATGCGCATGATGCCTACCTGAATGCAGTGGTAGGCACTGCACTTATCAAAAAATATCCCAA
GCTTGAATCTGAATTTGTTTACGGAGACTATAAAGTGTACGATGTTAGGAAAATGATCGCAAAGT
CTGAGCAGGAAATAGGCAAGGCCACCGCTAAGTACTTCTTTTACAGCAATATTATGAATTTTTTC
AAGACCGAGATTACACTGGCCAATGGAGAGATTCGGAAGCGACCACTTATCGAAACAAACGGAGA
AACAGGAGAAATCGTGTGGGACAAGGGTAGGGATTTCGCGACAGTCCGGAAGGTCCTGTCCATGC
CGCAGGTGAACATCGTTAAAAAGACCGAAGTACAGACCGGAGGCTTCTCCAAGGAAAGTATCCTC
CCGAAAAGGAACAGCGACAAGCTGATCGCACGCAAAAAAGATTGGGACCCCAAGAAATACGGCGG
ATTCGATTCTCCTACAGTCGCTTACAGTGTACTGGTTGTGGCCAAAGTGGAGAAAGGGAAGTCTA
AAAAACTCAAAAGCGTCAAGGAACTGCTGGGCATCACAATCATGGAGCGATCAAGCTTCGAAAAA
AACCCCATCGACTTTCTCGAGGCGAAAGGATATAAAGAGGTCAAAAAAGACCTCATCATTAAGCT
TCCCAAGTACTCTCTCTTTGAGCTTGAAAACGGCCGGAAACGAATGCTCGCTAGTGCGGGCGAGC
TGCAGAAAGGTAACGAGCTGGCACTGCCCTCTAAATACGTTAATTTCTTGTATCTGGCCAGCCAC
TATGAAAAGCTCAAAGGATCTCCCGAAGATAATGAGCAGAAGCAGCTGTTCGTGGAACAACACAA
ACACTACCTTGATGAGATCATCGAGCAAATAAGCGAATTCTCCAAAAGAGTGATCCTCGCCGACG
CTAACCTCGATAAGGTGCTTTCTGCTTACAATAAGCACAGGGATAAGCCCATCAGGGAGCAGGCA
GAAAACATTATCCACTTGTTTACTCTGACCAACTTGGGCGCGCCTGCAGCCTTCAAGTACTTCGA
CACCACCATAGACAGAAAGCGGTACACCTCTACAAAGGAGGTCCTGGACGCCACACTGATTCATC
AGTCAATTACGGGGCTCTATGAAACAAGAATCGACCTCTCTCAGCTCGGTGGAGACAGCAGGGCT
GATTCGGACCCGAAGAAAAAGAGGAAGGTAGATCCTAAGAAGAAGAGAAAGGTACTGTTGGATCC
GGGAACACCTATGGACGCTGATTTGGTAGCTTCTTCTACAGTAGTGTGGGAACAAGATGCAGATC
CTTTTGCTGGAACAGCCGACGACTTCCCCGCCTTCAATGAAGAGGAGCTTGCATGGTTGATGGAG
CTGTTACCTCAAGGAGGATCAGGGGGTCTTTTAGATCCAGGTACTCCCATGGATGCAGACCTGGT
CGCTTCAAGTACAGTCGTTTGGGAGCAGGATGCTGATCCATTCGCAGGTACAGCAGATGACTTCC
CGGCTTTCAATGAGGAGGAACTTGCTTGGCTCATGGAATTGCTCCCGCAGGCAAGAGGTGGATCT
GGTGGTCTGCTCGACCCAGGAACGCCGATGGACGCTGATCTGGTAGCATCTAGTACAGTCTGGGA
GCAGGACGCTGACCCATTCGCTGGTACGGCTGACGATTTTCCTGCCTTTAATGAGGAAGAGTTAG
CCTGGCTGATGGAGTTATTACCCCAGGGCGGTAGTGGCGGATTATTGGACCCTGGTACGCCGATG

GATGCAGACCTTGTGGCTTCATCAACGGTCGTATGGGAGCAGGATGCCGATCCTTTCGCAGGAAC
CGCAGACGACTTCCCAGCCTTTAACGAAGAAGAGCTCGCATGGCTTATGGAACTTTTACCTCAGG
CACGTGGAGGAAGTGGTGGGCTCCTGGATCCAGGCACTCCGATGGACGCAGACTTAGTAGCTAGT
TCCACCGTTGTCTGGGAGCAGGATGCAGATCCTTTTGCTGGTACCGCAGACGACTTCCCTGCTTT
CAATGAAGAAGAATTGGCCTGGCTTATGGAATTACTTCCGCAGGGTGGGTCTGGTGGGCTGCTCG
ATCCCGGTACGCCGATGGATGCTGACTTGGTTGCTAGCAGTACTGTGGTCTGGGAACAGGATGCT
GACCCCTTCGCTGGGACTGCCGACGACTTCCCAGCCTTCAACGAAGAGGAACTCGCATGGTTGAT
GGAACTGCTTCCTCAAGCACGTGGGGGGTCAGGCGGAGGCGGATCTGGCGGTGACGCACTGGACG
ACTTTGATCTCGACATGCTTGGGTCCGATGCTCTCGACGATTTTGACCTCGATATGCTTGGGTCT
GACGCATTAGATGACTTTGATTTAGACATGTTGGGGTCAGACGCTTTGGACGATTTCGACTTAGA
TATGTTGGCCAGAGGTAGTGATGCCCTTGACGACTTCGATTTAGATATGTTGGGCTCTGACGCCC
TCGATGACTTCGATCTGGATATGCTCGGAAGTGACGCCTTGGACGATTTCGACTTGGACATGCTT
GGGTCGGACGCCCTCGACGACTTTGATCTGGATATGTTATGAGCTTGGAATGGATCTTCGATCCC
GATCGTTCAAACATTTGGCAATAAAGTTTCTTAAGATTGAATCCTGTTGCCGGTCTTGCGACGAT
TATCATATAATTTCTGTTGAATTACGTTAAGCATGTAATAATTAACATGTAATGCATGACGTTAT
TTATGAGATGGGTTTTTATGATTAGAGTCCCGCAATTATACATTTAATACGCGATAGAAAACAAA
ATATAGCGCGCAAACTAGGATAAATTATCGCGCGCGGTGTCATCTATGTTACTAGATCGGGAATT
GCCAAGCTAATTCCTTCGTTGAACAACGGAAACTCGACTTGCCTTCCGCACAATACATCATTTCT
TCTTAGCTTTTTTTCTTCTTCTTCGTTCATACAGTTTTTTTTTGTTTATCAGCTTACATTTTCTT
GAACCGTAGCTTTCGTTTTCTTCTTTTTAACTTTCCATTCGGAGTTTTTGTATCTTGTTTCATAG
TTTGTCCCAGGATTAGAATGATTAGGCATCGAACCTTCAAGAATTTGATTGAATAAAACATCTTC
ATTCTTAAGATATGAAGATAATCTTCAAAAGGCCCCTGGGAATCTGAAAGAAGAGAAGCAGGCCC
ATTTATATGGGAAAGAACAATAGTATTTCTTATATAGGCCCATTTAAGTTGAAAACAATCTTCAA
AAGTCCCACATCGCTTAGATAAGAAAACGAAGCTGAGTTTATATACAGCTAGAGTCGAAGTAGTG
ATTGAGACGTTCGAAGCTTCTGTCGTCTCAGTTTTAGAGCTAGAAATAGCAAGTTAAAATAAGGC
TAGTCCGTTATCAACTTGAAAAAGTGGCACCGAGTCGGTGCTTTTTTTTTGAAGACGAAAGGGCC
TCGTGATACGCCTATTTTTATAGGTTAATGTCATGATAATAATGGTTTCTTAGACGTCAGGTGGC
ACTTTTCGGGGAAATGTGCGCGGAACCCCTATTTGTTTATTTTTCTAAATACATTCAAATATGTA
TCCGCTCATGAGACAATAACCCTGATAAATGCTTCAATAATGGGACCGACTCGCGCTTGAGACGA
AGCTGGCGCGCCAAGCTTGGCACTGGCCGTCGTTTTACAACGTCGTGACTGGGAAAACCCTGGCG
TTACCCAACTTAATCGCCTTGCAGCACATCCCCCTTTCGCCAGCTGGCGTAATAGCGAAGAGGCC
CGCACCGATCGCCCTTCCCAACAGTTGCGCAGCCTGAATGGCGAATGAGCTTGAGCTTGGATCAG
ATTGTCGTTTCCCGCCTTCAGTTTAAACTATCAGTGTTTGACAGGATATATTGGCGGGTAAACCT
AAGAGAAAAGAGCGTTTATTAGAATAACGGATATTTAAAAGGGCGTGAAAAGGTTTATCCGTTCG
TCCATTTGTATGTGCATGCCAACCACAGGGTTCCCCTCGGGATCAA3’
